# A novel HIF-2α targeted inhibitor suppresses hypoxia-induced breast cancer stemness via SOD2-mtROS-PDI/GPR78-UPR^ER^ axis

**DOI:** 10.1038/s41418-022-00963-8

**Published:** 2022-03-17

**Authors:** Yuanyuan Yan, Miao He, Lin Zhao, Huizhe Wu, Yanyun Zhao, Li Han, Binbin Wei, Dongman Ye, Xuemei Lv, Yan Wang, Weifan Yao, Haishan Zhao, Bo Chen, Zining Jin, Jian Wen, Yan Zhu, Tao Yu, Feng Jin, Minjie Wei

**Affiliations:** 1 Department of Pharmacology, School of Pharmacy, China Medical University, Shenyang, Liaoning Province China; 2 Liaoning Key Laboratory of molecular targeted anti-tumor drug development and evaluation; Liaoning Cancer immune peptide drug Engineering Technology Research Center; Key Laboratory of Precision Diagnosis and Treatment of Gastrointestinal Tumors, Ministry of Education, Shenyang, Liaoning Province China; 3 Department of Medical Imaging, Cancer Hospital of China Medical University, Liaoning Cancer Hospital & Institute, Shenyang, Liaoning Province China; 4 Department of Breast Surgery, The First Affiliated Hospital of China Medical University, 155 Nanjing North Street, Heping District, Shenyang, Liaoning Province China; 5 Department of Breast Surgery, The Fourth Affiliated Hospital of China Medical University, No.4 Chongshan East Road, Shenyang, Liaoning China; 6 Liaoning Medical Diagnosis and Treatment Center, Shenyang, Liaoning Province China

**Keywords:** Cancer microenvironment, Cancer stem cells, Protein aggregation

## Abstract

Hypoxic tumor microenvironment (TME) plays critical roles in induction of cancer stem cell-like phenotype in breast cancer and contribute to chemoresistance. However, the mechanism underlying stemness reprogramming of breast cancer cells (BCs) by hypoxic TME remains largely unknown. In the present study, we illustrated that HIF-2α, but not HIF-1α, induces stemness in BCs under hypoxia through SOD2-mtROS-PDI/GRP78-UPR^ER^ pathway, linking mitochondrial metabolic state to endoplasmic reticulum (ER) response via mitochondrial reactive oxygen species (mtROS) level. HIF-2α activates endoplasmic reticulum unfolded protein response (UPR^ER^) in drug-sensitive MCF7 and T47D cells to induce drug-resistant stem-like phenotype. Genetic depletion or pharmacological inhibition (YQ-0629) of HIF-2α abolished hypoxia-induced stem-like phenotype in vitro and in vivo. Mechanistically, HIF-2α activates transcription of superoxide dismutase 2 (SOD2) under hypoxia and thereby decreases mtROS level. With less mtROS transported to endoplasmic reticulum, the expression and activity of protein disulfide isomerase (PDI) is suppressed, allowing glucose-regulated protein 78 (GRP78) to dissociate from receptor proteins of UPR^ER^ and bind misfolded protein to activate UPR^ER^, which eventually confer chemoresistance and stem-like properties to BCs. Moreover, the increase in mtROS and PDI levels caused by HIF-2α knockdown and the subsequent UPR^ER^ inhibition could be substantially rescued by mitoTEMPOL (a mtROS scavenger), 16F16 (a PDI inhibitor), or GRP78 overexpression. Overall, we reported the critical roles of HIF-2α-SOD2-mtROS-PDI/GRP78-UPR^ER^ axis in mediating hypoxia-induced stemness in BCs, highlighting the interaction between organelles and providing evidence for further development of targeted HIF-2α inhibitor as a promising therapeutic strategy for chemoresistant breast cancer.

## Introduction

Solid tumor microenvironment (TME) is often characterized by hypoxia, which promotes the transformation of breast cancer cells (BCs) to breast cancer stem cells (BCSCs) [1]. Such transformation confers therapeutic resistance to breast cancer and limits clinical treatment benefits [2–5]. Hypoxia-induced transcription factors (HIFs) including HIF-2α (also known as *EPAS1*) play important roles in regulating cancer cell stemness by activating multiple transcriptional programs under hypoxia [6–8]. Our previous study showed that HIF-2α promotes the chemoresistant stem-like phenotype in BCs via activation of Wnt and Notch pathways [9]. These results highlight the plasticity of cancer cells and indicate the complicated composition of cancer stem cells (CSCs), supporting the function of HIF-2α in CSC phenotype conversion and chemoresistance acquisition.

HIF-2α is involved in stemness reprogramming and acquisition of chemoresistance through various pathways, and is often related to poor clinical outcomes [10–12]. For example, HIF-2α enhances ALKBH5-mediated m^6^A-demethylation of NANOG mRNA to induce the BCSC phenotype [6]. HIF-2α also regulates Wnt/β-catenin signaling to maintain pancreatic cancer stemness [13]. Previous studies of the stemness conversion have mainly focused on mitochondria [14, 15], whereas multiple organelles are actually involved in the stemness conversion process and the underlying mechanism remains unclear.

Hypoxia in TME alters protein synthesis by endoplasmic reticulum unfolded protein response (UPR^ER^) [16–19]. Emerging evidence has revealed that HIF-2α regulates UPR^ER^ to promote tumorigenesis and cancer cell survival, and suppresses apoptosis in leukemic cells under hypoxia [20]. Recent work has unveiled a critical role of UPR^ER^ in cell identity remodeling and acquisition of pluripotency, through the regulation of mitochondrial reactive oxygen species (mtROS) generation and protein-folding environment [21–23]. In this study, we found that UPR^ER^ was activated under hypoxia to induce the stemness remodeling via a HIF-2α/mtROS-dependent signaling pathway, which eventually contributes to the chemoresistance of BCs. Mechanistically, HIF-2α decreased mtROS levels by transcriptionally up-regulating SOD2, causing less mtROS to be transported from mitochondria to ER which, in turn, suppressed PDI expression and activity in ER. PDI competes with GRP78 for binding to misfolded protein to allow GRP78 bind with UPR^ER^ sensors and act as an “off” switch for UPR^ER^. The inhibition of PDI enhanced binding of GRP78 to misfolded protein, causing dissociation of GRP78 from UPR^ER^ sensors and ultimately activation of UPR^ER^. Moreover, we found that a novel HIF-2α targeted inhibitor, YQ-0629, sensitized tumor cells to paclitaxel in mouse PDX model, implying the clinical relevance of the reported HIF-2α-SOD2-mtROS-PDI/GRP78-UPR^ER^ axis. This work provides crucial insights into hypoxia-dependent stemness reprogramming and identifies a novel mechanism involving crosstalk between mitochondria and ER, encouraging the development of HIF-2α-targeting strategies for breast cancer patients, especially those who are resistant to chemotherapy.

## Results

### HIF-2α mediates hypoxia-induced breast cancer cell stemness remodeling and chemotherapy resistance

**Fig. 1:**
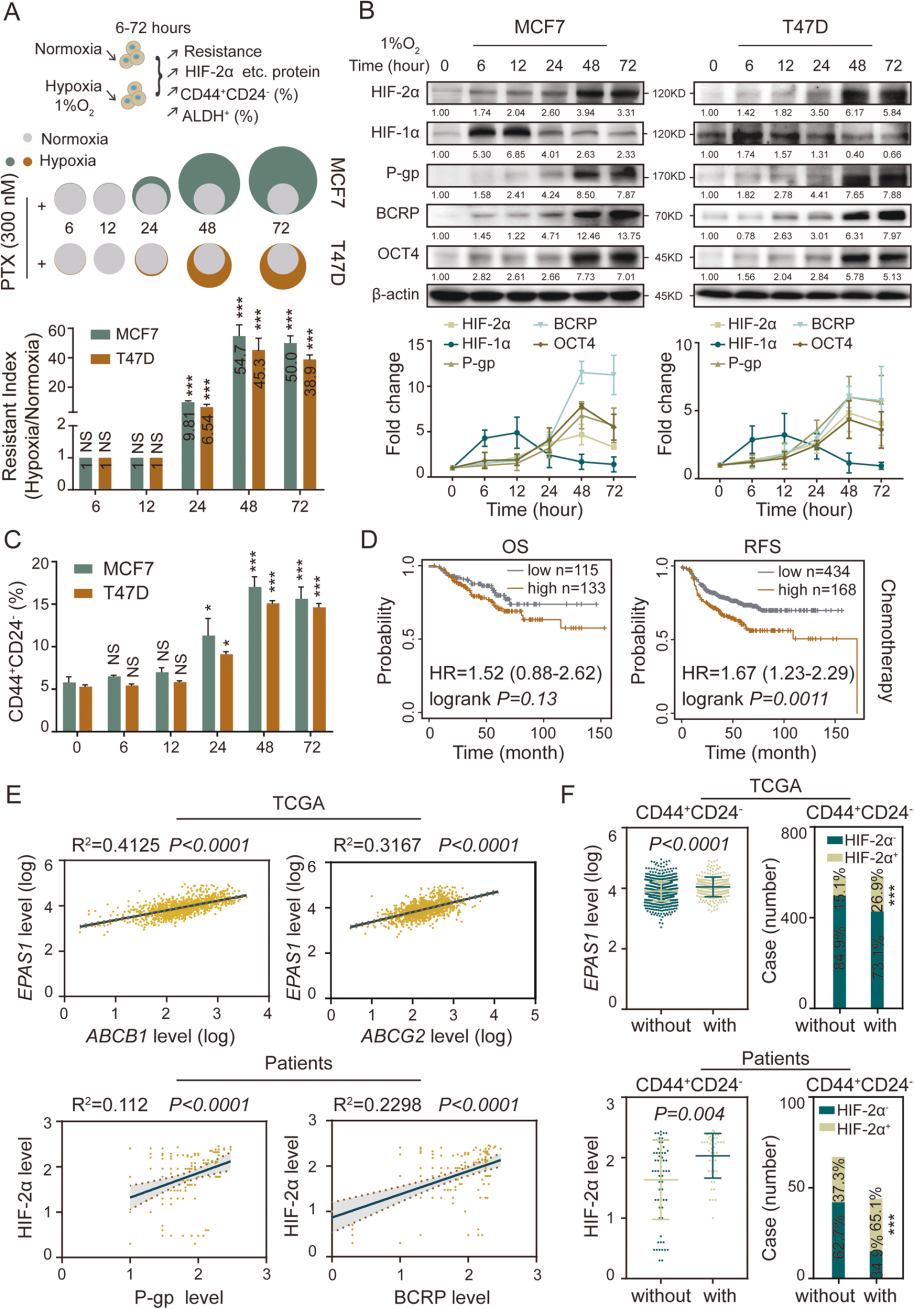
Hypoxia induces stemness reprogramming of BCs by activating HIF-2α. **A** The cell viability rate was detected in MCF7 and T47D cells cultured with different concentrations of paclitaxel (PTX) under hypoxia for 0–72 h by MTT assay. The gray color represents normoxia (20% O_2_), green and brown color represents hypoxia (1% O_2_); the area indicates the cell viability rate. IC_50_ fold change and resistance index (RI) values were compared relative to normoxia. h, hour. **B** The expression levels of HIF-2α, HIF-1α, P-gp, BCRP and OCT4 in MCF7 and T47D cells were detected by western bolt under hypoxia for 0–72 h. **C** The proportion of CD44^+^CD24^−^ subpopulation in MCF7 and T47D cells were detected by flow cytometry under hypoxia for 0–72 h. **D** The survival was analyzed between HIF-2α high expression and low expression cases received chemotherapy in overall survival (OS) and relapse-free survival (RFS) by Kaplan–Meier plot analysis (http://kmplot.com/analysis/index.php?p=service). **E** The correlations were analyzed among HIF-2α, P-gp, and BCRP in mRNA and protein levels from TCGA database (*n* = 1169) and our sample’s bank (*n* = 110). **F** The mRNA and protein levels of HIF-2α (*EPAS1*) was compared in CD44^+^CD24^−^ and non-CD44^+^CD24^−^ patients from TCGA dataset (*n* = 1169) and our sample’s bank (*n* = 110) (left panel). The right panel displays correlations between HIF-2α^+^ and CD44^+^CD24^−^ phenotype in mRNA and protein level. NS non-significant; *P < 0.05, ***P < 0.001, compared to 0 h/normoxia; Student’s *t* test, One-way ANOVA test, Pearson correlation analysis, Mann–Whitney *U* analysis, Pearson χ^2^ test. Error bars, mean ± SD (*n* = 3).

Hypoxia induces stemness in BCs [24]. To explore whether hypoxia is responsible for chemoresistance acquisition in BCs, we exposed MCF7 and T47D cells to paclitaxel (PTX) under hypoxia (1% O_2_) or normoxia (20% O_2_). MCF7 and T47D showed no significant change in IC_50_ values and resistance index (RI) after being cultured under hypoxia for up to 12 h. However, MCF7 and T47D displayed higher IC_50_ values and RI after being cultured under hypoxia for 24, 48, and 72 h (Figs. 1A and  S1A, B). Furthermore, hypoxia also induced significant multi-chemoresistance of MCF7, BT474, and HCC1937 cells to adriamycin (ADR), mitoxantrone (MX) and cisplatin (DDP) (Fig. S1C, data of BT474 and HCC1937 not shown), confirming that chronic hypoxia induced chemoresistance in BCs.

HIF family members play important roles in stemness remodeling and chemoresistance acquisition, but the specific functions of HIF-1α and HIF-2α remains unclear. We observed that the expression of HIF-2α, but not HIF-1α, and resistance-related proteins P-gp (also known as *ABCB1*) and BCRP (also known as *ABCG2*) increased over time in MCF7 and T47D under hypoxia (Fig. 1B). Meanwhile, the expression of CSC marker OCT4 was elevated, and the percentage of CD44^+^CD24^−^ and ALDH1^+^ BCSC populations were increased (Figs. 1B, C and S1D). These results indicated that hypoxia promoted chemoresistance of BCs in a time-dependent manner, and induced their transformation into BCSCs, with a simultaneous and specific increase in HIF-2α expression. Higher HIF-2α expression also predicted shorter overall survival (OS) and relapse-free survival (RFS) in breast cancer patients receiving chemotherapy (Fig. 1D). The Pearson’s correlation analysis of the TCGA dataset containing 1169 breast cancer patients and our in-house dataset containing 110 breast cancer tissues confirmed that HIF-2α, but not HIF-1α, was positively correlated with P-gp and BCRP (Figs. 1E and S1E). Consistently, breast cancer tissues that were CD44^+^CD24^−^ had higher levels of HIF-2α (not HIF-1α), suggesting that patients with higher level of HIF-2α are likely to have a higher percentage of stem-like cells in their cancer tissues (Figs. 1F and S1F). Together, these results indicate that long-term hypoxia drives stemness remodeling and induces chemoresistance in BCs by up-regulating HIF-2α.

### HIF-2α regulates the GRP78-UPR^ER^ pathway to remodel stemness and drive chemoresistance in BCs

**Fig. 2:**
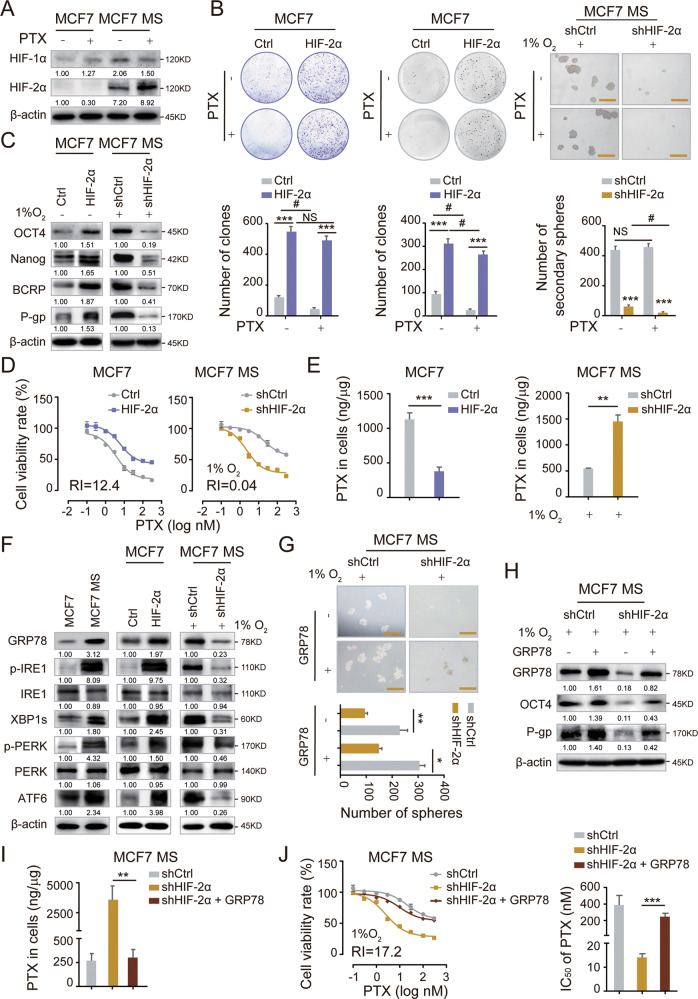
HIF-2α is required for the self-renewal maintenance of BCSCs by activating UPR^ER^. **A** The protein expression of HIF-1α and HIF-2α were measured in MCF7 and MCF7 MS cells cultured with or without PTX (3 nM) for 48 h by western blot. **B** The self-renewal ability was detected in HIF-2α-overexpressed (HIF-2α OE) MCF7 cells and HIF-2α-silencing (HIF-2α KD) MCF7 MS cells cultured with or without PTX (3 nM) for 48 h. Scale bar, 250 μm. **C** The protein expression of OCT4, NANOG, BCRP and P-gp were measured in HIF-2α OE MCF7 cells and HIF-2α KD MCF7 MS cells. **D** The cell viability rate was detected in HIF-2α OE MCF7 cells and HIF-2α KD MCF7 MS cells cultured with different concentrations of PTX for 48 h. Resistance index (RI) value was calculated. **E** The intracellular accumulation of PTX was detected in HIF-2α OE MCF7 cells and HIF-2α KD MCF7 MS cells by HPLC-MS. **F** The expression levels of GRP78, IRE1, XBP1s, PERK, and ATF6; phosphorylation levels of p-IRE1 and p-PERK were measured in MCF7 cells, MCF7 MS cells, HIF-2α OE MCF7 cells and HIF-2α KD MCF7 MS cells. **G** The self-renewal ability rescued in GRP78-overexpressing and HIF-2α-silencing (GRP78 OE + HIF-2α KD) MCF7 MS cells, compared with HIF-2α KD MCF7 MS cells. Scale bar, 250 μm. **H** The expression levels of GRP78, OCT4 and P-gp were measured in GRP78 OE + HIF-2α KD MCF7 MS cells. **I** The intracellular accumulation of PTX was detected in GRP78 OE + HIF-2α KD MCF7 MS cells by HPLC-MS. **J** The cells viability rate was detected in GRP78 OE + HIF-2α KD MCF7 MS cells cultured with different concentrations of PTX (left panel), the related IC_50_ of PTX were calculated (right panel). NS, non-significant; #*P* < 0.05, ##*P* < *0.01*, ###*P* < 0.001, compared to treatment without PTX, *P < 0.05, **P < 0.01, ***P < 0.001, compared to Ctrl/shCtrl/shHIF-2α; Student’s *t* test, two-way ANOVA test. Error bars, mean ± SD (*n* = 3).

We first generated MCF7 and T47D mammospheres (MCF7 MS and T47D MS), confirmed their BCSC-like properties and chemoresistance to PTX (Fig S2A–F, [25]). We observed that HIF-2α was highly expressed in MCF7 MS and T47D MS, PTX treatment further increased the expression of HIF-2α, but not HIF-1α (Figs. 2A and S3A). To further determine the function of HIF-2α, we overexpressed HIF-2α (HIF-2α OE) in MCF7 and T47D, as well as knocked down HIF-2α (HIF-2α KD) in MCF7 MS and T47D MS by lentiviral infection and compared the knockdown efficiency (Fig. S3E–G). HIF-2α overexpression significantly enhanced colony formation capacity of MCF7. In contrast, HIF-2α knockdown dramatically reduced the formation of primary and secondary mammospheres from MCF7 MS (Fig. 2B; data of primary mammosphere formation not shown), indicating that HIF-2α maintains the long-term self-renewal capacity of BCSCs. Similar results were achieved with T47D and T47D MS (Fig. S3H). Importantly, HIF-2α overexpression increased the expression of pluripotent transcription factors OCT4 and Nanog in MCF7 and T47D, and HIF-2α knockdown significantly decreased these expression in MCF7 MS and T47D MS (Figs. 2C and S3I). Moreover, we noticed that HIF-2α overexpression induced PTX resistance in MCF7, whereas HIF-2α knockdown remarkably increased the sensitivity of MCF7 MS to PTX treatment (Fig. 2D). We also achieved similar results T47D with and T47D MS and validated this finding (Fig. S3J). Consistently, HIF-2α overexpression increased the expression of drug resistance-related proteins BCRP and P-gp in MCF7 and T47D, and HIF-2α silencing in MCF7 MS and T47D MS reduced the expression of these proteins (Figs. 2C and S3I). Additionally, HIF-2α overexpression decreased the intracellular accumulation of PTX, which is transported by P-gp, and vice versa (Fig. 2E). Collectively, these data further confirmed that hypoxia-induced stemness remodeling and chemoresistant properties in BSCSs is mainly mediated by HIF-2α.

Cells undergo endoplasmic reticulum stress (ERS) when their ER homeostasis is disturbed by hypoxia [26], and they respond to ERS by activating UPR^ER^. UPR^ER^ activation has been reported to involve in pluripotency acquisition and chemotherapy resistance [22, 27]. Therefore, we examined the activation status of UPR^ER^ in MCF7 and MCF7 MS, as well as in HIF-2α-overexpressing MCF7 and HIF-2α-knockdown MCF7 MS. The expression levels of GRP78, XBP1s, and ATF6 as well as the phosphorylation levels of p-IRE1 and p-PERK were higher in MCF7 MS compared to MCF7 (Fig. 2F), suggesting that UPR^ER^ was activated in BCSCs. Consistently, HIF-2α-overexpression increased the expression and the phosphorylation levels of the aforementioned proteins in MCF7, whereas HIF-2α knockdown achieved opposite phenomenon in MCF7 MS (Fig. 2F), confirming the regulation of UPR^ER^ by HIF-2α. To determine whether HIF-2α maintains stemness and chemoresistance through the UPR^ER^, we tested if forced expression of GRP78 could restore stemness and chemoresistance in HIF-2α-knockdown MCF7 MS. Interestingly, forced expression of GRP78 rescued the oncosphere formation ability in HIF-2α depleted MCF7 MS (Fig. 2G). Additionally, forced expression of GRP78 also abolished HIF-2α-knockdown-induced inhibition of OCT4 and P-gp (Fig. 2H), increasing the intracellular accumulation of PTX and reducing chemoresistance (Fig. 2I, J). Collectively, these data indicate that HIF-2α drives stemness remodeling in BCs and induces chemoresistance by activating the GRP78-UPR^ER^.

### HIF-2α modulates SOD2 and mtROS level to activate UPR^ER^

**Fig. 3:**
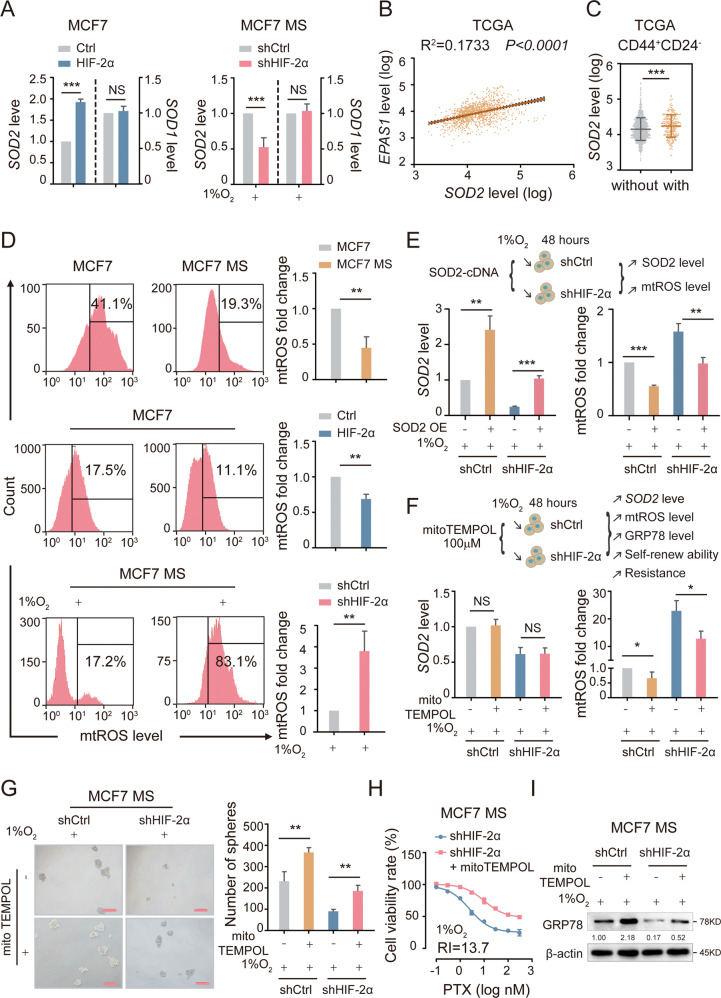
HIF-2α activates UPR^ER^ via regulating SOD2-mtROS axis. **A** The mRNA level of SOD2 and SOD1 were detected in HIF-2α OE MCF7 cells and HIF-2α KD MCF7 MS cells. **B** The correlation between HIF-2α and SOD2 was detected in mRNA level from TCGA database (*n* = 1169). **C** The mRNA expression of SOD2 was compared in CD44^+^CD24^−^ and non CD44^+^CD24^−^ patients from TCGA database (*n* = 1169). **D** The levels of mtROS were detected in MCF7 and MCF7 MS cells, HIF-2α OE MCF7 cells and HIF-2α KD MCF7 MS cells. **E** The mRNA level of SOD2 and mtROS level were measured in SOD2-overexpressing and HIF-2α-silencing (SOD2 OE + HIF-2α KD) MCF7 MS cells, under 1% O_2_. **F** The mRNA level of SOD2 and mtROS level were measured in HIF-2α KD MCF7 MS cells cultured with mitoTEMPOL (100 µM) for 48 h, under 1% O_2_. **G** The self-renewal ability rescued in HIF-2α KD MCF7 MS cells cultured mitoTEMPOL (100 µM) for 48 h, compared with HIF-2α KD MCF7 MS cells, under 1% O_2_. Scale bar, 250 μm. **H** The cells viability rate was detected in HIF-2α KD MCF7 MS cells cultured with mitoTEMPOL (100 µM) for 48 h, under 1% O_2_. **I** The expression level of GRP78 was detected in HIF-2α KD MCF7 MS cells cultured with mitoTEMPOL (100 µM) for 48 h, under 1% O_2_. NS, non-significant; *P < 0.05, **P < 0.01, ***P < 0.001, compared to MCF7 cells/Ctrl/shCtrl/shHIF-2α; Student’s *t* test, two-way ANOVA test, Mann–Whitney *U* analysis, Pearson correlation analysis. Error bars, mean ± SD (*n* = 3).

Next, we sought to investigate the detailed mechanism for the regulation of stemness and GRP78-UPR^ER^ by HIF-2α. The antioxidant gene superoxide dismutase 2 (SOD2) has been found to interact with HIF-2α [10, 28, 29]. SOD2 catalyzes the reduction of mtROS in the mitochondrial matrix, thereby protecting cells from oxidative damage [30–32]. To determine whether HIF-2α affects the SOD2-mtROS level to regulate the GRP78-UPR^ER^, we examined the mRNA expression of SOD2 in HIF-2α-overexpressing MCF7 and HIF-2α-knockdown MCF7 MS. Results showed that HIF-2α overexpression increased the mRNA level of SOD2, whereas HIF-2α knockdown reduced the expression of SOD2 (Fig. 3A). As a control, the mRNA level of SOD1 was not affected by either overexpression or knockdown of HIF-2α (Fig. 3A). Furthermore, we analyzed the TCGA dataset and found that HIF-2α expression was positively correlated with SOD2 expression, and patients with the CD44^+^CD24^−^ phenotype expressed SOD2 at a higher level (Fig. 3B, C). Together with the previous finding that patients with the CD44^+^CD24^−^ phenotype had a higher expression of HIF-2α (Fig. 1F), our results confirmed that HIF-2α activates SOD2 in BCSCs and this axis is clinically relevant. mtROS level was also lower in MCF7 MS than in MCF7 cells, indicating that the intracellular mtROS level of BCSCs was lower than that of BCs. Consistently, the overexpression or knockdown of HIF-2α decreased or increased the level of mtROS respectively (Fig. 3D). These data suggest that HIF-2α is an upstream regulator of SOD2 and mtROS.

To further validate whether HIF-2α regulates mtROS level in BCSCs via SOD2, we overexpressed SOD2 in HIF-2α-knockdown MCF7 MS. As expected, SOD2 overexpression abrogated the HIF-2α-knockdown-induced increase in mtROS level (Figs. 3E and S4A). We also incubated HIF-2α-knockdown MCF7 MS with mitoTEMPOL, a mtROS-specific scavenger, to further verify if mtROS mediate the downstream effects of HIF-2α. MitoTEMPOL treatment reduced the level of mtROS without affecting SOD2 expression (Figs. 3F and S4B), confirming the suitability of mitoTEMPOL for testing the role of mtROS in the function of HIF-2α. We then observed that mitoTEMPOL rescued the oncosphere formation capacity in HIF-2α-knockdown MCF7 MS (Fig. 3G). Moreover, mitoTEMPOL restored the chemoresistance of HIF-2α-knockdown MCF7 MS (Figs. 3H and S4C). Additionally, to explore whether UPR^ER^ is involved, we examined the protein level of GRP78 in the same groups. Our results showed that mitoTEMPOL rescued the protein expression of GRP78 in HIF-2α-knockdown MCF7 MS (Figs. 3I and S4D). In sum, HIF-2α upregulates SOD2 to reduce mtROS level, leading to the downstream biological effects including GRP78-UPR^ER^ activation, stemness remodeling, and chemoresistance acquisition.

### PDI competitively inhibits the binding of GRP78 to misfolded proteins to prevent UPR^ER^ activation

**Fig. 4:**
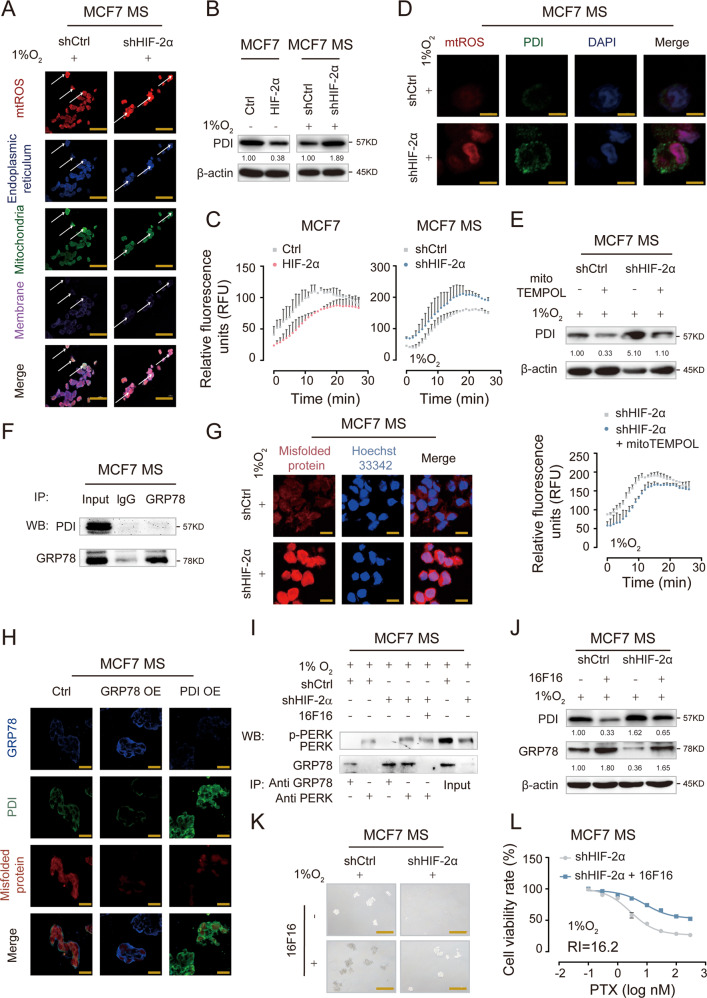
PDI competitively binding to misfolded proteins with GRP78 to activate UPR^ER^. **A** The location of mtROS in mitochondria and ER was observed by confocal microscope. Scale bar, 10 μm. **B** The protein expression of PDI was detected in HIF-2α OE MCF7 cells and HIF-2α KD MCF7 MS cells, under 1% O_2_. **C** The enzyme activities of PDI in HIF-2α OE MCF7 cells and HIF-2α KD MCF7 MS cells were measured, under 1% O_2_. **D** The level and location of mtROS and PDI were measured in HIF-2α KD MCF7 MS cells were detected by immunofluorescence microscopy, under 1% O_2_. Scale bar, 5 μm. **E** The protein expression and enzyme activity of PDI were measured in HIF-2α KD MCF7 MS cells cultured with mitoTEMPOL (100 µM), under 1% O_2_. **F** The direct interaction of GRP78 and PDI in MCF7 MS cells was determined by co-immunoprecipitation (co-IP). **G** The level of misfolded protein was detected in HIF-2α KD MCF7 MS cells by confocal microscope. Scale bar, 10 μm. **H** The expression levels of GRP78, PDI and misfolded proteins were detected in the GRP78-overexpressing (GRP78 OE), PDI-overexpressing (PDI OE) MCF7 MS cells by immunofluorescence microscopy, under 1% O_2_. Scale bar, 20 μm. **I** The combination and dissociation of GRP78 and PERK was confirmed by co-immunoprecipitation (co-IP) in HIF-2α KD MCF7 MS cells cultured with 16F16 (100 µM), under 1% O_2_. **J**–**L** The protein expressions of PDI and GRP78, cell viability rate and self-renewal ability were detected in HIF-2α KD MCF7 MS cells cultured with 16F16 (100 µM), under 1% O_2_. Scale bar, 250 μm.

MtROS are mainly generated during the process of oxidative phosphorylation at the mitochondrial electron transport chain and are transported from mitochondria to multiple intracellular organelles as signaling molecules [31, 32]. In particular, mtROS are transported to ER and are involved in the regulation of ER-related pathways [33]. To elucidate how the modulation of SOD2-mtROS level by HIF-2α is transduced intracellularly to activate UPR^ER^, we detected the subcellular location of mtROS. We observed that mtROS was mainly located in mitochondria in control cells, while the increased mtROS primarily accumulated in ER in HIF-2α-knockdown MCF7 MS (Fig. 4A), indicating that excess mtROS induced by HIF-2α knockdown were mainly transported from mitochondria to ER. Protein disulfide isomerase (PDI) is a critical protein in ER that has two major functions: one is to act as an oxidoreductase, and the other is to function as a chaperone protein to bind misfolded protein for subsequent degradation. The enzyme activity of PDI can be increased when the ER environment becomes more oxidative [34, 35]. Therefore, we hypothesized that HIF-2α might regulate PDI activity in ER through mtROS transport. To test our hypothesis, we first detected the expression and enzymatic activity of PDI after HIF-2α overexpression and knockdown. We found that the expression and enzymatic activity of PDI was reduced by HIF-2α overexpression in MCF7, whereas the opposite was observed upon HIF-2α knockdown in MCF7 MS (Fig. 4B, C). Not only that, HIF-2α silencing increased the levels of mtROS and PDI in MCF7 MS, with their subcellular location overlapping (Fig. 4D), supporting our hypothesis that mtROS might be transported to ER to regulate PDI. We then exposed HIF-2α-knockdown MCF7 MS to mitoTEMPOL and detected the expression and enzymatic activity of PDI (Fig S4E). We found that mitoTEMPOL significantly decreased PDI expression and enzymatic activity (Fig. 4E). These results suggest that HIF-2α regulates the expression and catalytic activity of PDI in BCSCs through the signaling molecules - mtROS.

Then, we explored the underlying mechanism by which PDI regulates the UPR^ER^. GRP78 functions as an “off” switch for UPR^ER^, hence we first mocked docking of PDI to GRP78, and found the two proteins did not match in spatial structure (Fig. S4F). We further confirmed that there was no direct interaction between PDI and GRP78 in MCF7 MS (Figs. 4F and S4G). Therefore, we presumed that a potential indirect mechanism mediates the regulation of UPR^ER^ by PDI. Given that the activation of UPR^ER^ depends on the binding of GRP78 to misfolded protein and the subsequent dissociation of GRP78 from the UPR^ER^ transmembrane protein sensors [36, 37], we detected the intracellular level of misfolded protein in HIF-2α-knockdown MCF7 MS. We found that the level of misfolded protein significantly increased upon HIF-2α knockdown (Figs. 4G and S4H). To search for potential interaction among PDI, GRP78, and misfolded proteins, we constructed stably GRP78 or PDI overexpressed MCF7 MS. We found the protein level of GRP78 was decreased in PDI-overexpressing MCF7 MS, and vice versa. Additionally, the level of misfolded protein was remarkably reduced in both GRP78-overexpressing and PDI-overexpressing MCF7 MS (Fig. 4H and S4I). 16F16 is a specific PDI inhibitor. Strikingly, 16F16 treatment enhanced the binding of GRP78 to misfolded proteins in HIF-2α-knockdown MCF7 MS, allowing the dissociation of GRP78 from PERK. This result confirmed that PDI competes with GRP78 for binding to misfolded proteins to activate UPR^ER^ (Fig. 4I). We also detected the expression of GRP78 to further validate if PDI regulates GRP78 to affect UPR^ER^. We found that 16F16 released the HIF-2α-knockdown-induced inhibition on GRP78 expression, as well as suppressed the expression and activity of PDI in HIF-2α-knockdown MCF7 MS (Figs. 4J and S4K). Consistently, 16F16 restored mammosphere forming ability and chemoresistance in HIF-2α-knockdown MCF7 MS (Figs. 4K, L and S4L, M). These results indicate that PDI competitively inhibits the binding of GRP78 to misfolded proteins to prevent the activation of UPR^ER^ and HIF-2α activates UPR^ER^ by suppressing PDI expression and activity.

### HIF-2α silencing reduces stemness and reverses chemoresistance via SOD2-mtROS-UPR^ER^ pathway in vivo

**Fig. 5:**
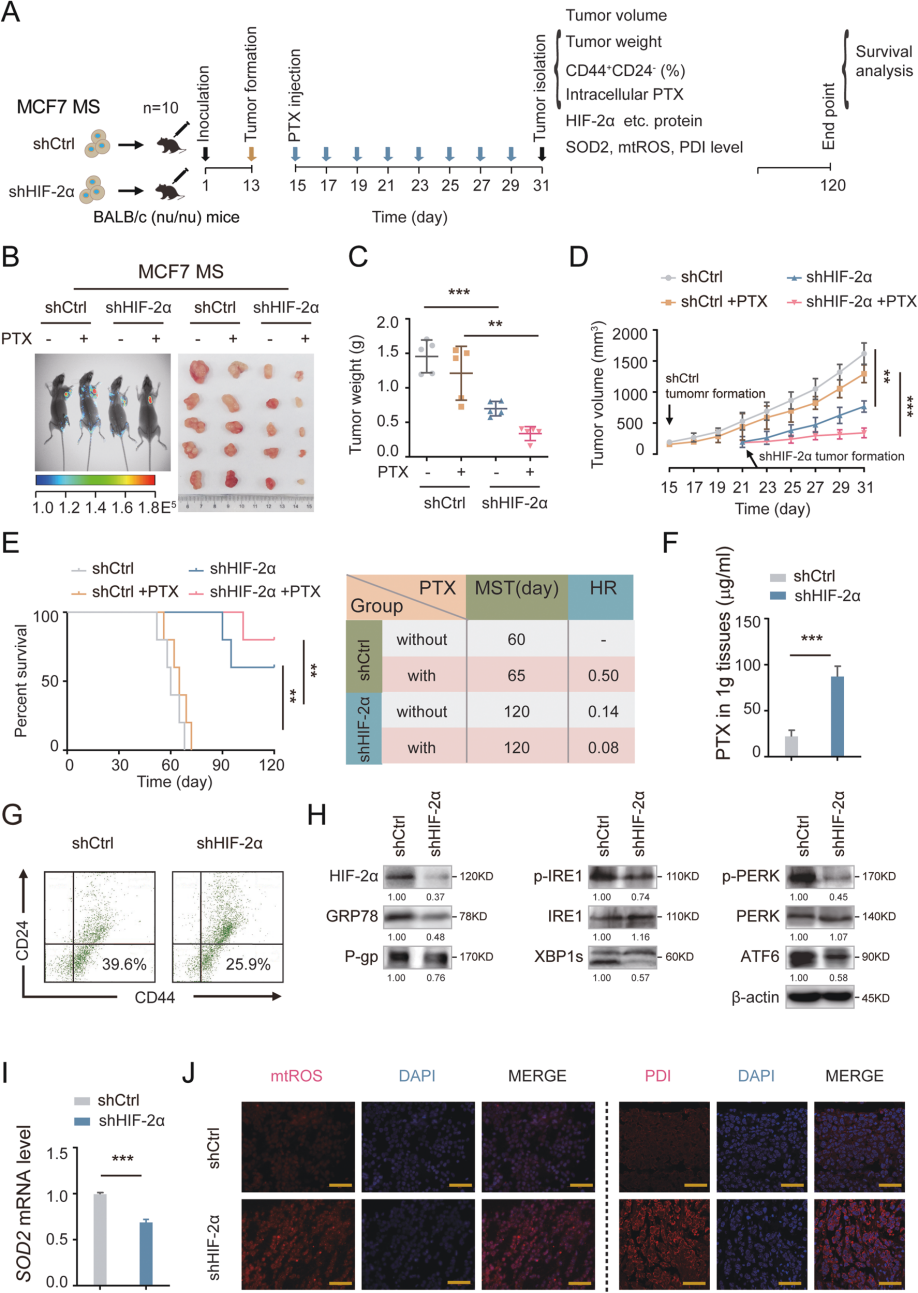
Silencing HIF-2α suppresses the early tumorigenesis and increases the sensitivities of PTX via SOD2-mtROS-PDI/GRP78-UPR^ER^ in vivo. **A** The diagram showed the time of tumor formation in BALB/c (nu/nu) mice transplanted with control or HIF-2α KD MCF7 MS cells (1 × 10^5^). 15 days after the inoculation, the mice were intraperitoneally injected with or without PTX (5 mg/kg) once every other day till to the 31th day (*n* = 5). The other mice (*n* = 5) were observed survival till the 120th day. **B** Small animal imaging showed the expression of green fluorescent protein (GFP) in the xenografted mice of each group. *n* = 5. **C** The tumor weights were measured in each group after sacrifice of xenograft mice at the 31th days. *n* = 5. Two-way ANOVA test. **D** The growth curves of tumor volumes were measured in xenograft mice were measured every other day. *n* = 5. Two-way ANOVA test. **E** The survival of the mice in each group (left) were analyzed by Kaplan Meier-plotter curve. Median survival times (MST). Hazard ratio (HR). *n* = 5. Two-way ANOVA test. **F** The PTX accumulation were measured in the HIF-2α KD xenografted tissue by HPLC-MS. *n* = 3. Student’s *t* test. **G** The proportion of CD44^+^CD24^−^ cell in the HIF-2α KD xenografted tumor were detected by flow cytometry. **H** The protein expressions of HIF-2α, GRP78, P-IRE1, IRE1, XBP1s, p-PERK, PERK, ATF6 and P-gp were detected in the HIF-2α KD xenografted tissue. **I** The mRNA expression of SOD2 was detected in the HIF-2α KD xenografted tissue. *n* = 3. Student’s *t* test. **J** The level of mtROS and PDI were detected in the HIF-2α KD xenografted tissue. Scale bar, 100 μm. *P < 0.05, **P < 0.01, ***P < 0.001, compared to shCtrl.

We next confirmed the role of HIF-2α in tumorigenesis in vivo. We injected BALB/c (nu/nu) mice subcutaneously with control or HIF-2α-knockdown MCF7 MS, and monitored tumor growth as well as measured the percentage of BCSC population and related pathway indicators (Fig. 5A). HIF-2α depletion remarkably suppressed the growth of xenograft tumors, and the inhibitory effect was even stronger when treated in combination with PTX (Fig. 5B, C). CSCs are responsible for early tumorigenesis [24]. We defined the formation of a detectable tumor as when a tumor reaches the of size 125 mm^3^ and observed xenograft tumor formation at about 1 month post injection. HIF-2α depletion resulted in a significant delay in tumor formation (at day 21) compared to the control group (at day 15), suggesting that HIF-2α knockdown reduces the tumor initiating ability of MCF7 MS (Fig. 5D). Additionally, we observed that HIF-2α knockdown significantly improved survival in the xenograft mouse model, especially when treated in combination with PTX (Fig. 5E). The accumulation of PTX in HIF-2α-knockdown xenograft tissues was much higher than that in control tissues (Fig. 5F). Furthermore, we found that the percentage of CD44^+^CD24^−^ cells was also lower in HIF-2α-knockdown xenograft tissues than in control tissues (Fig. 5G). These findings imply that HIF-2α depletion decreases self-renewal capacity and reverses chemoresistance in BCSCs in vivo. Consistently, HIF-2α depletion dramatically reduced the expression of P-gp, GRP78, and other UPR^ER^-related proteins in vivo (Fig. 5H). Similar results were obtained for the mRNA level of SOD2 (Fig. 5I). The mtROS level and PDI expression were also increased in HIF-2α-knockdown xenograft tissues, compared to control tissues (Fig. 5J). Overall, these data indicate that HIF-2α silencing impairs stemness and chemoresistance of BCSCs through SOD2-mtROS-PDI/GRP78-UPR^ER^ pathway in vivo.

### A specific HIF-2α inhibitor YQ-0629 synergizes with PTX in vivo

**Fig. 6:**
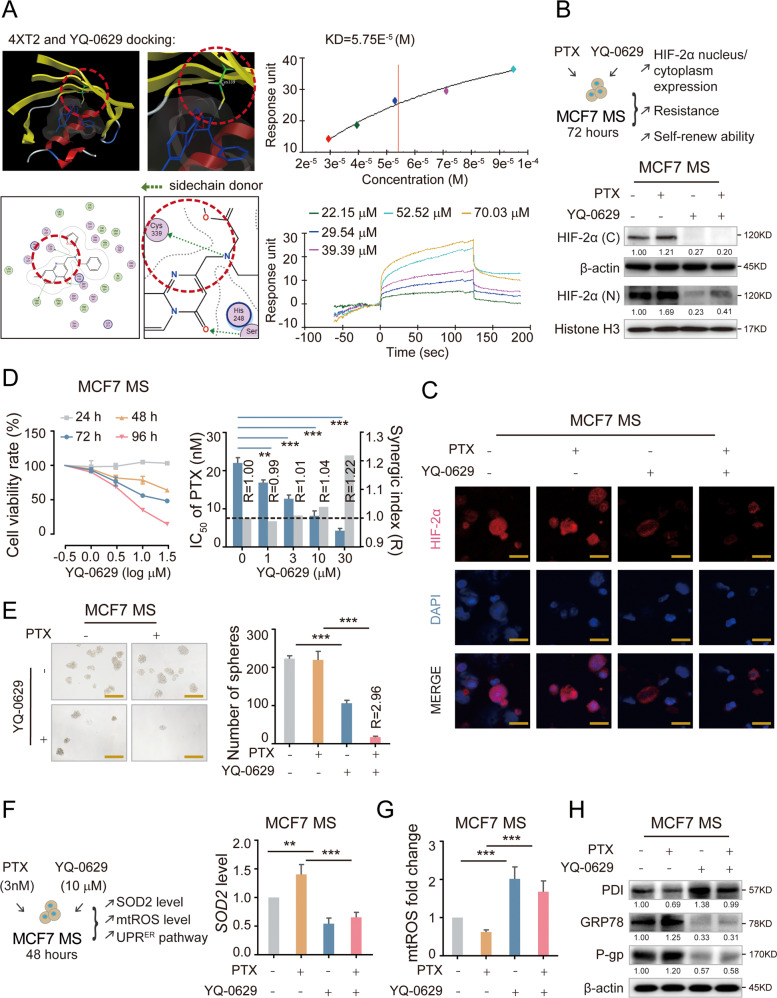
YQ-0629 targets HIF-2α to suppress stem trait of BCSCs and synergy the sensitization to PTX in vitro. **A** The chemical structure of YQ-0629 and docking conformation showed the interaction of the YQ-0629 with the active site of HIF-2α through MOE software (left). YQ-0629 and HIF-2α PAS-B domain was confirmed direct binding by surface plasmon resonance (SPR)-based Biacore assay (right). **B** The expression level of HIF-2α was detected in the nucleus (N) and cytoplasm (C) of MCF7 MS cells cultured with YQ-0629 (10 µM) alone, PTX (3 nM) alone, or YQ-0629 (10 µM) combined with PTX (3 nM) for 72 h. **C** The expression and location of HIF-2α were detected in MCF7 MS cells cultured with YQ-0629 (10 µM) alone, PTX (3 nM) alone, or YQ-0629 (10 µM) combined with PTX (3 nM) for 72 h by immunofluorescence staining. Scale bar, 10 μm. **D** The cell viability rate of MCF7 MS cells cultured with different concentrations of YQ-0629 for 24–96 hours were determined by CCK-8 assay (left). The IC_50_ values of PTX and synergic index (R) were calculated in MCF7 MS cells cultured with indicated dose of YQ-0629 for 72 h (right). *n* = 3. Student’s *t* test. **E** The self-renewal ability was detected in MCF7 MS cells cultured with YQ-0629 (10 µM) alone, PTX (3 nM) alone, or YQ-0629 (10 µM) combined with PTX (3 nM) for 72 h. *n* = 3. Synergic index (R). Two-way ANOVA test. Scale bar, 250 μm. **F**–**H** The mRNA expression of SOD2 and mtROS level, the expressions level of PDI, GRP78, and P-gp were detected in the MCF7 MS cells cultured with indicated dose of PTX and YQ-0629 for 72 h. *n* = 3. Two-way ANOVA test. **P < 0.01, **P < 0.01, ***P < 0.001, compared to MCF7 MS cells/MCF7 MS cells + PTX treatment.

To further explore the clinical relevance of HIF-2α and the reported downstream pathway in breast cancer, we aimed to find a specific HIF-2α inhibitor. The HIF-2α protein contains three domains with known structures: a b-Helix-loop-helix (bHLH) segment, Per-Arnt-Sim (PAS) domains, and terminal transactivation domains (TADs). PAS-B domain is the potential active site for ligand binding [38]. The amino acid sequence targeted by the HIF-2α shRNA we used was located in PAS-B domain (Fig. S5A). We found the crystal structure of the PAS-B region from Protein Data Bank (PDB ID: 4XT2), and it has already been proved that active small molecule compounds binding this active site can be successfully screened out [39]. First, we used two libraries of small molecular compounds containing a total of 1 million compounds for docking into this site. 81 compounds (Table 1) with S values less than −9 were screened out. 17 compounds with high drug-forming property were further screened out based on ADME evaluation. Three compounds including YQ-0629 were demonstrated strong inhibitory effects on cell viability. We further performed a Biacore assay to verify the binding of the three compounds to HIF-2α active sites in vitro (Fig. S5B). Only YQ-0629 showed a strong affinity with HIF-2α PAS-B domain (dissociation constant [Kd] 5.75E^−5^ M) and the potential binding site is predicted to be the residue Cys339 (Fig. 6A). To investigate whether YQ-0629 inhibits HIF-2α in vivo, we detected the protein expression and intracellular location of HIF-2α after MCF7 MS culturing with YQ-0629 (10 µM). We observed that YQ-0629 could inhibit the expression of HIF-2α with or without the presence of PTX (3 nM) (Fig. 6B, C). We also confirmed that YQ-0629 did not bind to HIF-1α or affect the expression of HIF-1α (Fig. S5C, D). These data indicate that YQ-0629 is a specific inhibitor of HIF-2α.

**Table 1:** The reault of docking by MOE.

mol	rseq	mseq	S	rmsd_refine	FP:PLIF	PLIF_ligidx	E_conf	E_place	E_score1	E_refine	E_score2	a_acc	a_don	logP(o/w)	logS	SlogP	TPSA	Weight
Fc1cc(C)c(NC( = O)CN2C( = O)C(N(C( = O)CC)C) = Nc3c2cccc3)cc1	1	194	−10.938254	1.7831233	1 2 6 13	[[10 10 10 10 10] [10 10 10 10 10] 21 21]	73.83429	−65.439285	−9.6604252	−36.246143	−10.938254	4	1	2.046	−4.86901	3.0178199	82.080002	396.422
O = C(N(C)C = 1 C( = O)N(CC( = O)Nc2ccc(OC)cc2)c2c(N = 1)cccc2)CC	1	195	−10.912217	1.6352755	4	14	53.837105	−64.515892	−9.6641216	−30.77556	−10.912217	5	1	1.516	−4.46394	2.5789001	91.309998	394.431
Fc1cc(C)c(NC( = O)CN2C( = O)C(N(C( = O)CC)C) = Nc3c2cccc3)cc1	1	194	−10.709622	1.3912883	1 2 6 13	[[10 10 10 10 10] [10 10 10 10 10] 21 21]	66.496262	−86.975899	−9.3012972	−32.216003	−10.709622	4	1	2.046	−4.86901	3.0178199	82.080002	396.422
Fc1c(NC( = O)CN2C( = O)C(N(C( = O)CC)C) = Nc3c2cccc3)ccc(F)c1	1	191	−10.616098	1.5885445	1 2 66 6 13	[[10 10 10 10 10] [10 10 10 10 10] 26 21 21]	87.036522	−74.541992	−9.252636	−36.326653	−10.616098	4	1	1.901	−5.00352	2.8485	82.080002	400.385
Fc1cc(C)c(NC( = O)CN2C( = O)C(N(C( = O)CC)C) = Nc3c2cccc3)cc1	1	194	−10.552382	1.3751938	1 2 6	[[10 10 10 10 10] [10 10 10 10 10] 21]	80.821815	−90.90596	−9.8919563	−31.125942	−10.552382	4	1	2.046	−4.86901	3.0178199	82.080002	396.422
Clc1ccc(NC( = O)CN2C( = O)C(N(C( = O)CC)C) = Nc3c2cccc3)cc1	1	192	−10.400867	1.2550629	6 13	[17 17]	81.382957	−66.724831	−9.3333635	−29.644501	−10.400867	4	1	2.152	−5.14785	3.2237	82.080002	398.85
Clc1ccc(NC( = O)CN2C( = O)C(N(C( = O)CC)C) = Nc3c2cccc3)cc1	1	192	−10.360132	1.2362972	1 6	[[9 9 9 9 9] 17]	83.137192	−61.30851	−9.4128256	−29.487608	−10.360132	4	1	2.152	−5.14785	3.2237	82.080002	398.85
Fc1c(NC( = O)CN2C( = O)C(N(C( = O)CC)C) = Nc3c2cccc3)ccc(F)c1	1	191	−10.352764	2.0071845	1 2 66 6 13	[[10 10 10 10 10] [10 10 10 10 10] 26 21 21]	79.611908	−48.871712	−9.5374498	−31.998219	−10.352764	4	1	1.901	−5.00352	2.8485	82.080002	400.385
O = C(N(CCOC)Cc1nc(COc2cc(C)ccc2)sc1)CC	1	206	−10.315837	1.373821		[]	53.685596	−63.378716	−10.607458	−36.705509	−10.315837	4	0	2.608	−2.92336	3.9483199	51.66	348.467
O = C(N(CCOC)Cc1nc(COc2c(C)c(C)cc(C)c2)sc1)CC	1	202	−10.18891	1.1696758		[]	49.33707	−72.034058	−11.159831	−23.807611	−10.18891	4	0	3.237	−3.55775	4.5651598	51.66	376.521
O = C(N(CCOC)Cc1nc(COc2c(C)c(C)cc(C)c2)sc1)CC	1	202	−10.165962	1.6515702		[]	44.253685	−54.726841	−9.7377605	−20.87141	−10.165962	4	0	3.237	−3.55775	4.5651598	51.66	376.521
O = C(N(CCOC)Cc1nc(COc2cc(C)ccc2)sc1)CC	1	206	−10.154015	1.8054231		[]	47.665268	−73.104424	−9.5320988	−32.237152	−10.154015	4	0	2.608	−2.92336	3.9483199	51.66	348.467
O = C(N(CCOC)Cc1nc(COc2cc(C)ccc2)sc1)CC	1	206	−10.148293	1.1232054	10	6	41.12479	−74.544441	−10.236934	−33.357601	−10.148293	4	0	2.608	−2.92336	3.9483199	51.66	348.467
O = C(N(CCOC)Cc1nc(COc2c(C)c(C)cc(C)c2)sc1)CC	1	202	−10.147232	1.6236573	37 26 29 3	[22 6 6 22]	46.595928	−68.276367	−9.9389849	−22.807604	−10.147232	4	0	3.237	−3.55775	4.5651598	51.66	376.521
Fc1cc(C)c(NC( = O)CN2C( = O)C(N(C( = O)CC)C) = Nc3c2cccc3)cc1	1	194	−10.083389	1.8171558	45	21	82.07254	−79.647301	−9.9758043	−26.444155	−10.083389	4	1	2.046	−4.86901	3.0178199	82.080002	396.422
O = C(N(CCOC)Cc1nc(COc2cc(C)c(C)cc2)sc1)CC	1	205	−10.046181	1.2799406		[]	46.098122	−87.489403	−9.7152643	−27.573259	−10.046181	4	0	2.904	−3.39728	4.2567401	51.66	362.494
O = C(N(CCOC)Cc1nc(COc2c(C)c(C)cc(C)c2)sc1)CC	1	202	−10.021337	1.0244079		[]	47.566261	−94.499832	−11.015378	−21.993591	−10.021337	4	0	3.237	−3.55775	4.5651598	51.66	376.521
O = C(N(CCOC)Cc1nc(COc2c(C)c(C)cc(C)c2)sc1)CC	1	202	−10.013167	2.2391009	51	6	47.740715	−43.478626	−9.8501482	-17.340368	−10.013167	4	0	3.237	−3.55775	4.5651598	51.66	376.521
O = C(N(CCOC)Cc1nc(COc2cc(C)c(C)cc2)sc1)CC	1	205	−9.8725662	1.3905082	10	6	47.896034	−45.029846	−10.007674	−24.523638	−9.8725662	4	0	2.904	−3.39728	4.2567401	51.66	362.494
Fc1ccc(NC( = O)CN2C( = O)C(N(C( = O)CC)C) = Nc3c2cccc3)cc1	1	200	−9.81569	1.0334991		[]	83.840248	−78.816048	−9.863452	−26.088251	−9.81569	4	1	1.713	−4.70854	2.7093999	82.080002	382.395
Fc1ccc(NC( = O)CN2C( = O)C(N(C( = O)CC)C) = Nc3c2cccc3)cc1	1	200	−9.8077593	1.3528427		[]	95.229691	−60.510231	−9.8809338	−26.250235	−9.8077593	4	1	1.713	−4.70854	2.7093999	82.080002	382.395
Fc1ccc(NC( = O)CN2C( = O)C(N(C( = O)CC)C) = Nc3c2cccc3)cc1	1	200	−9.7154455	1.441017	1 2 6	[[9 9 9 9 9] [9 9 9 9 9] 17]	72.269409	−82.974785	−10.168523	−25.800505	−9.7154455	4	1	1.713	−4.70854	2.7093999	82.080002	382.395
Brc1ccc(NC( = O)CN2C( = O)C(N(C( = O)CC)C) = Nc3c2cccc3)cc1	1	150	−9.6700525	1.3923471	6	17	90.105148	−40.248928	−9.6419649	−18.469656	−9.6700525	4	1	2.358	−5.50395	3.3327999	82.080002	443.301
O = C(N(CCOC)Cc1nc(COc2cc(C)ccc2)sc1)CC	1	206	−9.5716019	1.651099	39	22	51.783993	−68.938942	−10.407656	−27.115789	−9.5716019	4	0	2.608	−2.92336	3.9483199	51.66	348.467
Fc1cc(C)c(NC( = O)CN2C( = O)C(N(C( = O)CC)C) = Nc3c2cccc3)cc1	1	194	−9.5423374	1.8602611		[]	90.812431	−76.622543	−10.010983	−16.224051	−9.5423374	4	1	2.046	−4.86901	3.0178199	82.080002	396.422
O = C(N(C)C = 1 C( = O)N(CC( = O)Nc2ccc(C)cc2)c2c(N = 1)cccc2)CC	1	201	−9.5378523	1.4987394	1 6	[[9 9 9 9 9] 17]	83.412437	−68.649963	−8.6353951	−17.296104	−9.5378523	4	1	1.858	−4.88748	2.87872	82.080002	378.432
Fc1ccc(NC( = O)CN2C( = O)C(N(C( = O)CC)C) = Nc3c2cccc3)cc1	1	200	−9.5346565	1.4158621	1 6	[[9 9 9 9 9] 17]	85.169342	−68.123856	−10.437311	−23.037125	−9.5346565	4	1	1.713	−4.70854	2.7093999	82.080002	382.395
O = C(N(C)C = 1 C( = O)N(CC( = O)Nc2cc(C)c(C)cc2)c2c(N = 1)cccc2)CC	1	198	−9.5189009	1.6912936		[]	62.66856	−33.40675	−8.6570959	−12.775396	−9.5189009	4	1	2.191	−5.3614	3.18714	82.080002	392.459
Clc1ccc(NC( = O)CN2C( = O)C(N(C( = O)CC)C) = Nc3c2cccc3)cc1	1	192	−9.4825964	1.6797754	6	17	84.100311	−80.925598	−9.1933918	−22.722916	−9.4825964	4	1	2.152	−5.14785	3.2237	82.080002	398.85
O = C(N(CCOC)Cc1nc(COc2cc(C)ccc2)sc1)CC	1	206	−9.4407234	1.1775275		[]	46.461788	−60.646614	−9.5731077	−24.327597	−9.4407234	4	0	2.608	−2.92336	3.9483199	51.66	348.467
O = C(N(C)C = 1 C( = O)N(CC( = O)Nc2ccc(OC)cc2)c2c(N = 1)cccc2)CC	1	195	−9.4121208	1.8469723	6	17	104.67824	−77.250793	−9.8469458	−17.24333	−9.4121208	5	1	1.516	−4.46394	2.5789001	91.309998	394.431
Brc1ccc(NC( = O)CN2C( = O)C(N(C( = O)CC)C) = Nc3c2cccc3)cc1	1	150	−9.3616257	1.5611087	1 2 6 13	[[9 9 9 9 9] [9 9 9 9 9] 17 17]	85.092247	−91.566315	−10.05342	−16.262087	−9.3616257	4	1	2.358	−5.50395	3.3327999	82.080002	443.301
O = C(N(CCOC)Cc1nc(COc2cc(C)c(C)cc2)sc1)CC	1	205	−9.3223333	1.5094248	28 60	[[22 44 43 42 23] [22 44 43 42 23]]	74.316414	−51.635536	−12.669622	−20.374947	−9.3223333	4	0	2.904	−3.39728	4.2567401	51.66	362.494
Brc1ccc(NC( = O)CN2C( = O)C(N(C( = O)CC)C) = Nc3c2cccc3)cc1	1	150	−9.2992764	1.781745	1 2 6	[[9 9 9 9 9] [9 9 9 9 9] 17]	82.239342	−42.763515	−9.2397318	−16.375626	−9.2992764	4	1	2.358	−5.50395	3.3327999	82.080002	443.301
O = C(N(C)C = 1 C( = O)N(CC( = O)Nc2ccc(C)cc2)c2c(N = 1)cccc2)CC	1	201	−9.2312002	1.4929682		[]	118.03013	−46.209911	−8.457756	−16.017235	−9.2312002	4	1	1.858	−4.88748	2.87872	82.080002	378.432
O = C(N(C)C = 1 C( = O)N(CC( = O)Nc2c(C)cc(C)cc2)c2c(N = 1)cccc2)CC	1	197	−9.200736	1.8307776		[]	68.142166	−68.19297	−9.8695173	−11.049742	−9.200736	4	1	2.191	−5.04795	3.18714	82.080002	392.459
O = C(N(CCOC)Cc1nc(COc2cc(C)c(C)cc2)sc1)CC	1	205	−9.1963415	1.5497842	39	22	59.502342	−64.809776	−12.160249	−20.262173	−9.1963415	4	0	2.904	−3.39728	4.2567401	51.66	362.494
O = C(N(C)C = 1 C( = O)N(CC( = O)Nc2ccc(OC)cc2)c2c(N = 1)cccc2)CC	1	195	−9.1187716	1.6638592	3 14	[14 14]	84.711357	−53.852581	−8.5630455	−14.621253	−9.1187716	5	1	1.516	−4.46394	2.5789001	91.309998	394.431
Fc1c(NC( = O)CN2C( = O)C(N(C( = O)CC)C) = Nc3c2cccc3)ccc(F)c1	1	191	−9.1121922	1.4418428	1 2	[[10 10 10 10 10] [10 10 10 10 10]]	92.921974	−67.421486	−10.055465	−19.784554	−9.1121922	4	1	1.901	−5.00352	2.8485	82.080002	400.385
FC(F)(F)c1c(OC)ccc(C( = O)Nc2cc(C(F)(F)F)cc(-c3onc4c3CCCC4)c2)c1	1	104	−9.1094694	1.8124239	16	[[5 9 8 7 6]]	141.37689	−59.811256	−7.6863132	−1.4543294	−9.1094694	2	1	6.00752	−7.64869	7.14184	64.360001	484.396
Clc1c(OC)ccc(NC( = O)CN2C( = O)C(N(C( = O)CC)C) = Nc3c2cccc3)c1	1	167	−9.0432253	1.5727638	1 6	[[33 33 33 33 33] 22]	111.14684	−94.654655	−10.549702	−10.234877	−9.0432253	5	1	2.143	−5.19823	3.2323	91.309998	428.876
Fc1ccc(NC( = O)CN2C( = O)C(N(C( = O)CC)C) = Nc3c2cccc3)cc1	1	200	−9.0261774	1.1828657	6 13	[17 17]	97.467995	−78.931458	−10.31346	−18.311417	−9.0261774	4	1	1.713	−4.70854	2.7093999	82.080002	382.395
O = C(NCc1occc1)CCCc1sc(C( = O)Nc2ccc(C)cc2)nn1	1	204	−10.460769	1.9126331	4 16	[12 12]	32.185101	−54.236809	−8.3161516	−33.550163	−10.460769	4	2	2.356	−4.63966	3.59729	97.120003	384.46
O = C1N2C( = NC(CN(Cc3occc3)Cc3ccccc3)=C1)C = C(C)C = C2	1	45	−10.189792	0.8146282	12 13 1 14	[4 4 [34 41 43 47 45 39] 13]	62.182995	−86.637306	−10.502284	−29.542992	−10.189792	3	0	2.653	−5.3023	4.4127998	49.049999	359.429
Clc1ccc(CSC = 2n3nc(-c4sccc4)cc3C(=O)NN = 2)cc1	1	127	−10.149225	0.8502005	4	15	122.89852	−85.182693	−11.545432	−41.840046	−10.149225	3	1	4.503	−6.32458	4.3273001	59.279999	374.876
S(Cc1ccc(C)cc1)C = 1n2nc(-c3sccc3)cc2C(=O)NN = 1	1	31	−10.102728	1.0717729	4	15	118.09198	−76.843628	−12.053961	−38.377987	−10.102728	3	1	4.209	−6.06421	3.9823201	59.279999	354.458
Clc1ccc(CSC = 2n3nc(-c4occc4)cc3C(=O)NN = 2)cc1	1	43	−9.9872217	0.9513763	3 4	[[12 16 20 18 14] 15]	142.22092	−78.559776	−11.87368	−43.721352	−9.9872217	3	1	3.688	−6.26948	3.8587999	72.419998	358.809
S(Cc1ccc(C)cc1)C = 1n2nc(-c3occc3)cc2C(=O)NN = 1	1	7	−9.9405518	0.6945121	3 4	[[12 16 20 18 14] 15]	137.4003	−98.104568	−12.835557	−39.99435	−9.9405518	3	1	3.394	−6.00911	3.5138199	72.419998	338.391
Fc1ccc(C(NC( = O)c2cc(OC)ccc2)c2oc(-c3ccccc3)nn2)cc1	1	385	−9.8510714	2.1505692	12 13 10	[14 14 9]	76.445915	−26.643658	−8.9426355	−23.171175	−9.8510714	4	1	4.972	−7.21102	4.4991999	77.25	403.413
O = C(Nc1cc(C( = O)C)ccc1)c1c2c(nc(-c3cnccc3)c1)cccc2	1	94	−9.8059988	1.1290568	12 13 10	[14 14 9]	3.94593	−62.999607	−12.244148	−26.206341	−9.8059988	4	1	3.674	−5.39272	4.7516999	71.949997	367.408
O = C(Nc1cc(OC)c(OC)c(OC)c1)c1c2c(nc(-c3cnccc3)c1)cccc2	1	552	−9.8008347	1.0850925	12 3 10	[20 [19 31 47 33 21 25] 8]	58.65123	−60.098019	−11.729462	−14.375378	−9.8008347	6	1	3.22548	−5.23159	4.5749002	82.57	415.449
Clc1ccc(CSC2 = NC = Cn3nc(-c4sccc4)cc23)cc1	1	41	−9.7324276	0.6792789		[]	9.6543798	−81.243019	−11.77093	−37.725327	−9.7324276	2	0	3.792	−6.02345	5.6532001	30.18	357.889
S(Cc1ccc(OC)cc1)C1 = NC = Cn2nc(-c3ccccc3)cc12	1	23	−9.5824881	1.1068726	2	13	7.6172404	−61.247097	−10.116899	−27.129368	−9.5824881	3	0	3.626	−5.71699	4.9468999	39.41	347.442
O(CCn1c(Cc2ccccc2)nc2c1cccc2)c1cc(C)ccc1	1	10	−9.5771179	0.8715386		[]	39.945278	−66.531174	−11.243786	−22.246656	−9.5771179	2	0	5.667	−5.65198	5.28089	27.049999	342.442
Clc1ccc(CSC2 = NC = Cn3nc(-c4ccccc4)cc23)cc1	1	29	−9.5222225	0.9016182	2	13	3.7078457	−74.063538	−11.286135	−32.970608	−9.5222225	2	0	4.262	−6.4009	5.5917001	30.18	351.861
S(Cc1ccc(C)cc1)C1 = NC = Cn2nc(-c3sccc3)cc12	1	6	−9.5210714	0.7866034		[]	4.9888377	−83.027557	−10.850096	−33.233433	−9.5210714	2	0	3.498	−5.76308	5.3082199	30.18	337.471
S(Cc1ccc(C)cc1)C1 = NC = Cn2nc(-c3ccccc3)cc12	1	3	−9.4389629	1.1168524	2	13	−0.938683	−84.930847	−10.968632	−29.40621	−9.4389629	2	0	3.968	−6.14053	5.2467198	30.18	331.443
O = C(Nc1cc2N(C( = O)c3occc3)CCCc2cc1)c1cc(OC)ccc1	1	139	−9.4326887	1.2540237	10 11	[17 17]	90.964813	−49.745277	−11.406919	−24.559771	−9.4326887	3	1	3.102	−5.40646	4.1334701	71.779999	376.412
Brc1ccc(CSC2 = NC = Cn3nc(-c4ccccc4)cc23)cc1	1	302	−9.3804808	1.0461311	2	13	7.9948578	−61.308563	−11.300909	−25.608639	−9.3804808	2	0	4.468	−6.757	5.7007999	30.18	396.312
S(Cc1ccc(C)cc1)C1 = NC = Cn2nc(-c3c(C)ccc(C)c3)cc12	1	55	−9.3762856	1.2313714		[]	9.3400259	−71.844727	−10.716241	−16.636683	−9.3762856	2	0	4.599	−7.08837	5.8635602	30.18	359.497
S(Cc1ccc(OC)cc1)C1 = NC = Cn2nc(-c3ccc(F)cc3)cc12	1	75	−9.3619413	1.3067884	2	13	18.820042	−57.730362	−9.95998	−25.407757	−9.3619413	3	0	3.779	−6.01197	5.086	39.41	365.432
Clc1ccc(CSC2 = NC = Cn3nc(-c4c(C)cccc4)cc23)cc1	1	88	−9.3481798	0.9533808	1	[[11 30 38 40 36 15]]	5.2840195	−74.416542	−10.528507	−27.199154	−9.3481798	2	0	4.558	−6.87482	5.9001198	30.18	365.888
N(Cc1ccc(C)cc1)C = 1n2nc(-c3occc3)nc2N = C(C)C = 1	1	1	−9.3377171	1.1166085		[]	124.29549	−63.576096	−11.017919	−31.1987	−9.3377171	3	1	1.921	−5.98017	3.8071201	68.239998	319.368
S( = O)( = O)(N(C)c1ccc(C)cc1)c1sc(NC( = O)/C = C/c2ccccc2)nn1	1	542	−9.3339987	2.4967248	10 4 16	[[3 11] 18 18]	30.843954	−44.116062	−9.7825909	−13.754408	−9.3339987	5	1	3.646	−6.61198	3.3235199	92.260002	414.51
O = C(Nc1cc(OC)c(OC)cc1)c1c2c(nc(-c3cnccc3)c1)cccc2	1	210	−9.3253307	1.1258233	12 3	[16 [14 31 43 33 18 24]]	17.510517	−74.765373	−13.496837	−13.778906	−9.3253307	5	1	3.48274	−5.18121	4.5662999	73.339996	385.423
O(C)c1ccc(CNC = 2n3nc(-c4cnccc4)nc3N = C(C)C = 2)cc1	1	21	−9.2783651	1.013528	1	[[11 30 43 37 14 18]]	95.112495	−80.941689	−12.355953	−23.961651	−9.2783651	5	1	1.631	−4.73104	3.3092999	77.220001	346.394
Fc1c(C( = O)Nc2cc(CCc3n(C)c4c(n3)cccc4)ccc2)cccc1	1	115	−9.2757092	1.3320879		[]	34.493172	−59.985386	−10.428094	−17.55064	−9.2757092	2	1	4.989	−5.65978	5.1090398	46.919998	373.431
S(CC( = O)N(C)c1ccccc1)C1 = NC(c2ccc(OC)cc2)=NC = 2N1NC( = O)C = 2	1	643	−9.2612324	1.3560218	10 11 4	[[9 12] 9 19]	36.53323	−63.482971	−11.788753	−13.550608	−9.2612324	5	1	3.668	−6.20003	2.3958001	86.599998	421.481
O = C(NCc1occc1)c1c(C)nc(COc2ccc(C)cc2)s1	1	9	−9.2602644	1.686085	5 6	[[1 4 5 3 2] 4]	27.167377	−58.323898	−12.913135	−27.493792	−9.2602644	3	1	2.534	−4.51044	4.3947401	64.360001	342.419
O(C)c1ccc(CNC = 2n3nc(-c4cnccc4)nc3N = C(CCC)C = 2)cc1	1	125	−9.2581339	1.5960585	20 21 1	[[8 8 8 8 8] [8 8 8 8 8] [11 30 43 34 14 18]]	168.3857	−49.01144	−10.639342	−14.098823	−9.2581339	5	1	2.548	−5.44803	4.0895	77.220001	374.448
Clc1c(CNC = 2n3c(nnc3)N = C(c3ccc(OC)cc3)C = 2)cccc1	1	81	−9.2345181	1.6097397	15 1	[8 [15 31 39 41 33 19]]	84.841797	−67.491676	−12.882171	−27.361973	−9.2345181	4	1	4.317	−5.79835	3.9291	64.330002	365.824
S(Cc1ccc(C)cc1)C1 = NC = Cn2nc(-c3c(C)cccc3)cc12	1	19	−9.2313328	1.0720456	1 2	[[11 29 41 43 35 15] 14]	0.95992428	−80.215492	−11.268885	−24.245464	−9.2313328	2	0	4.264	−6.61445	5.55514	30.18	345.47
Clc1ccc(CSC2 = NC = Cn3nc(-c4ccc(F)cc4)cc23)cc1	1	97	−9.2252741	1.1121234	2	13	13.107337	−69.086746	−10.447957	−28.599001	−9.2252741	2	0	4.415	−6.69588	5.7308002	30.18	369.851
O = C(Nc1ccc(C)cc1)c1sc(C2N(C( = O)c3ccccc3)CCC2)nn1	1	281	−9.1642857	1.8345664	5 24	[[1 5 6 2 3] [1 5 6 2 3]]	64.915665	−66.42865	−10.25529	−14.549685	−9.1642857	4	1	3.873	−5.18932	4.1716199	75.190002	392.483
O = C(Nc1ccccc1)CC1N(Cc2occc2)C( = O)N(c2cc(OC)ccc2)C1 = O	1	607	−9.1215267	1.2033887	10 11	[19 19]	−31.805082	−33.053543	−10.671512	−5.9882545	−9.1215267	4	1	2.22	−5.20056	3.9208	92.089996	419.437
O = C(Nc1c(OC)cc(OC)cc1)c1c2c(nc(-c3cnccc3)c1)cccc2	1	211	−9.0648451	1.6063622	3	[[15 31 43 33 17 24]]	25.543804	−70.667473	−11.988891	−9.2061634	−9.0648451	5	1	3.731	−5.18121	4.5662999	73.339996	385.423
Clc1ccc(CSC = 2n3nc(-c4ccc(C)cc4)cc3C(=O)NN = 2)cc1	1	194	−9.0527763	1.4399766	15 1 2 4	[14 [13 17 24 28 26 15] 12 14]	138.23361	−91.044739	−10.411779	−18.228752	−9.0527763	3	1	5.271	−7.17595	4.5742202	59.279999	382.875
O = C(Nc1cc2N(C( = O)c3occc3)CCCc2cc1)c1cc(OC)c(OC)cc1	1	430	−9.039814	1.4730424	10 11	[21 21]	112.04241	−70.091591	−11.564325	−11.970048	−9.039814	4	1	2.80774	−5.45684	4.1420698	81.010002	406.438
Clc1ccc(CN(C)c2c(-c3onc(-c4c(C)cccc4)n3)cccn2)cc1	1	273	−9.0395203	1.1121174	19	19	78.985283	−76.667534	−11.215223	−10.100689	−9.0395203	3	0	5.036	−7.99421	5.6632199	55.049999	390.874
O = C(Nc1c(CCc2n(C)c3c(n2)cccc3)cccc1)Cc1cc(OC)ccc1	1	337	−9.0243988	1.3242083	8 26	[7 7]	7.9607992	−86.649765	−12.920856	−5.0245686	−9.0243988	3	1	4.88	−5.47665	4.9074101	56.150002	399.494
S(Cc1ccc(C = C)cc1)C1 = NC = Cn2nc(-c3c(C)cccc3)cc12	1	40	−9.0214014	0.8013189	1 2	[[11 34 42 44 40 15] 14]	15.069262	−78.46283	−10.606567	−16.755856	−9.0214014	2	0	4.8	−7.23722	5.88972	30.18	357.481

YQ-0629 significantly inhibited cell viability of MCF7 MS in a time- and dose-dependent manner (Fig. 6D, left panel). We treated MCF7 MS with YQ-0629 and PTX to test if a synergistic effect exists between the two drugs. We observed that the IC_50_ values of PTX at 72 h gradually decreased with the increase of YQ-0629 dose. Importantly, the synergistic indices (R values) were all greater than 1, with a greatest R value of 1.22 when PTX is used in combination with 30 μM YQ-0629, implying the presence of a synergistic effect between YQ-0629 and PTX on tumor suppression (Fig. 6D, right panel). Notably, YQ-0629 treatment either used alone or in combination with PTX (R = 2.96), impaired the self-renewal ability of MCF7 MS (Fig. 6E). Additionally, the effect of YQ-0629 treatment in MCF7 MS was consistent with that of HIF-2α depletion on downstream pathway related parameters (Fig. 6F, G). Collectively, these data suggested that YQ-0629, as a HIF-2α targeted inhibitor, suppresses self-renewal capacity and synergizes with PTX to reverse the chemoresistance of MCF7 MS.

**Fig. 7:**
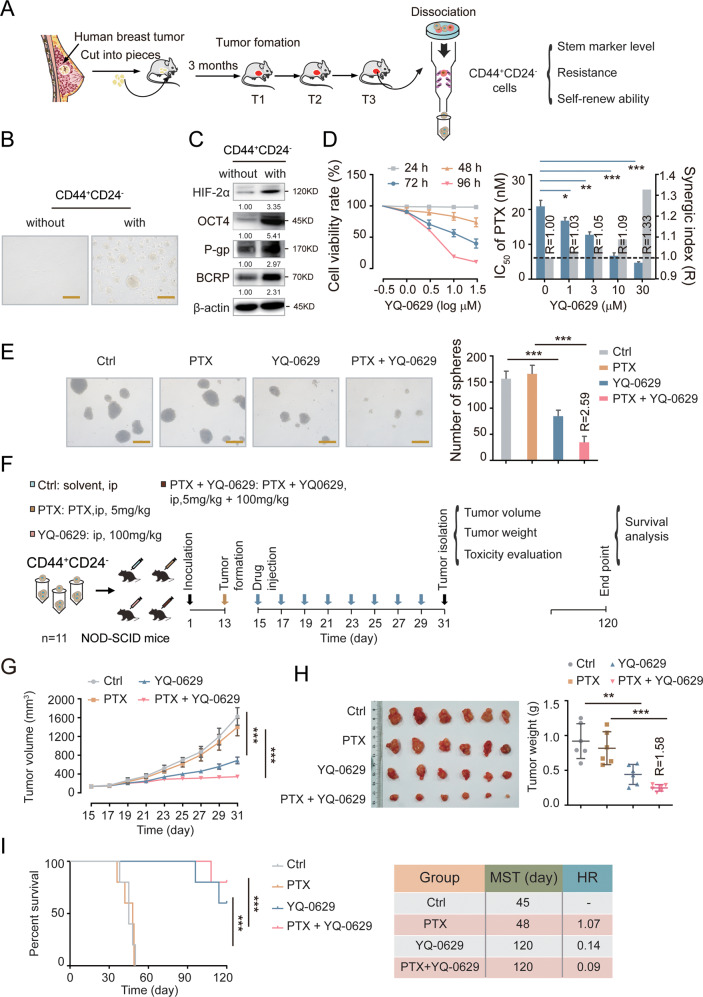
YQ-0629 inhibits growth of BCSCs derived from breast cancer patients and synergistically increases anti-BCSCs activity of PTX in vivo. **A** The diagram showed the process of sorting out CD44^+^CD24^−^ phenotype cells from primary human BCs by MACS. **B** The mammosphere’s morphology of CD44^+^CD24^−^ cells were examined. Scale bar, 250 μm. **C** The expression of HIF-2α, OCT4, P-gp, and BCRP were detected in CD44^+^CD24^−^ cells. **D** The cell viability rate of CD44^+^CD24^−^ cells cultured with different concentrations of YQ-0629 for 24–72 h were determined by CCK-8 assay (left). The IC_50_ values of PTX and synergic index (R) were calculated in the MCF7 MS cells cultured with indicated dose of YQ-0629 for 72 h. *n* = 3. Student’s *t* test. **E** The self-renewal ability was measured in CD44^+^CD24^−^ cells cultured with YQ-0629 (10 µM) alone, PTX (3 nM) alone, or YQ-0629 (10 µM) combined with PTX (3 nM) for 72 h. *n* = 3. Synergic index (R). Two-way ANOVA test. Scale bar, 250 μm. **F** The diagram showed the time of tumor formation in NOD-SCID mice transplanted with CD44^+^CD24^−^ cells (1 × 10^5^). 15 days after the inoculation, the mice were intraperitoneally injected with PTX (5 mg/kg), or YQ-0629 (100 mg/kg), or PTX (5 mg/kg) plus YQ-0629 (100 mg/kg), or PEG-35 castor oil as control, once every other day till to the 31th day for test (*n* = 6). And the other mice (*n* = 5) were observed survival till the 120th day. **G** The growth curves of tumor volumes were measured in xenograft mice of each group every other day (*n* = 6). Two-way ANOVA test. **H** The tumor weights in each group were measured (*n* = 6). Synergic index (R). Two-way ANOVA test. **I** The survival of the mice was analyzed in each group by Kaplan Meier-plotter curves. Median survival times (MST). Hazard ratio (HR). (*n* = 5). Two-way ANOVA test. *P < 0.05, **P < 0.01, ***P < 0.001, compared to non CD44^+^CD24^−^ cells/Ctrl/Ctrl + PTX treatment.

Next, we conducted a more stringent preclinical assessment on the efficacy of YQ-0629. First, we sorted out the CD44^+^CD24^−^ BCSC population from the clinical samples of breast cancer patients and detected the expression levels of stemness-related, chemoresistance-related, and self-renewal-related markers (Fig. 7A). The CD44^+^CD24^−^ cells displayed BCSC properties (Fig. 7B, C). Next, we treated the CD44^+^CD24^−^ cells with YQ-0629 and found that YQ-0629 inhibited cell viability in a time- and dose-dependent manner (Fig. 7D, left panel). Similarly, a synergistic effect was observed between PTX and YQ-0629 (R = 1.33, Fig. 7D, right panel). Consistently, YQ-0629 alone or in combination with PTX (R = 2.59) decreased mammosphere forming ability (Fig. 7E). We further explored the effect of YQ-0629 in vivo (Fig. 7F). YQ-0629 remarkably suppressed xenograft tumor growth and prolonged survival in the transplanted mice, especially when combined with PTX (Fig. 7G, I), demonstrating that YQ-0629 synergizes with PTX not only for the inhibition of tumor growth (R = 1.58) but also for the prolongation of survival. Finally, we evaluated the toxicity of YQ-0629 in vital organs. No noticeable change to the bladder, heart, kidney, liver and lung was observed, indicating that YQ-0629 was safe at the dosage we used (Fig. S5E). Overall, our data demonstrated that YQ-0629 specifically targets HIF-2α to inhibit BCSC self-renewal capacity via the SOD2-mtROS-PDI/GRP78-UPR^ER^ pathway and synergizes with PTX to overcome chemoresistance in BCSCs.

## Discussion

Here, we provided compelling in vitro and in vivo evidences that HIF-2α, induced by hypoxic TME, promotes the transition of chemosensitive BCs to chemoresistant BCSCs. Mechanistically, HIF-2α suppresses PDI, which competes with GRP78 for binding to misfolded proteins, to allow GRP78 dissociation from sensors of UPR^ER^ to turn on “the switch” of UPR^ER^ and thereby promote stemness in BCs. More specifically, HIF-2α decreased mtROS production and transportation from mitochondria to ER by transcriptionally upregulating SOD2, which consequently inhibited the expression and activity of PDI and promoted the binding of GRP78 with misfolded proteins. More importantly, we screened out a small molecule HIF-2α inhibitor that synergizes with PTX for inhibition of tumor growth in vitro and in vivo, and represents a promising candidate for targeted therapy of breast cancer.

In the adaptive response to hypoxia, HIF-2α acts as a crucial regulator of cancer stemness [13, 40–42]. Consistent with previous studies, we confirmed that HIF-2α was responsible for the elevation of OCT4 [43], P-gp [44], and BCRP [45] under hypoxia. Furthermore, we showed that HIF-2α, but not HIF-1α, was highly expressed in CD44^+^CD24^−^ breast cancer tissues. Emerging evidence demonstrates that hypoxia induces cellular reprogramming to reshape cancer cell stemness [46]. However, current studies mainly focus on changes occurring inside mitochondria, such as impairment of oxidative phosphorylation, promotion of glycolytic activity, and increase in mtROS production [47]. Our study added another layer to the mechanism by which HIF-2α regulates the signal transduction between mitochondria and ER in breast cancer. We showed that HIF-2α promoted breast cancer stemness via its regulation of UPR^ER^. These findings highlight the significance of UPR^ER^ activation in HIF-2α-induced stemness and chemoresistance acquisition.

UPR^ER^ is crucial for cancer stemness acquisition and maintenance. Although previous report revealed a potential interaction between HIF-2α and UPR^ER^ in hematopoietic stem and progenitor cells (HSPCs) [20], we found that HIF-2α regulates UPR^ER^ differently in BC. First, all three downstream pathways of UPR^ER^ (IRE1, PERK, and ATF6) were inhibited in HIF-2α-knockdown BCSCs. Second, in our study, although an increase in mtROS transportation from mitochondria to ER was also observed in HIF-2α-knockdown BCSCs, this event led to dysregulation of protein synthesis and impairment of stemness in BCSCs rather than induction of apoptosis. We speculate that the extent of hypoxia in TME and the HIF-2α expression level vary tremendously among different tissues and might contribute to such discrepancy, but the underlying reason need to be further investigated.

Aggregation of misfolded proteins in ER triggers ERS, and UPR^ER^ is involved in this event to control cell fate [36]. GRP78 is a sentinel marker of ERS and is widely used as a “switch” for UPR^ER^ activation [48]. In addition to the mechanism that GRP78 disassociates from ER transmembrane protein sensors and activates UPR^ER^ via binding to misfolded proteins, misfolded proteins are also regulated by GRP78 via other mechanisms [49]. The complete mechanism of this complicated process needs to be further clarified by more studies. Here we found that PDI [34], a multifunctional redox chaperone, was repressed for its activity and expression when the transportation of mtROS from mitochondria to ER was decreased. Like GRP78, PDI was also capable of binding to and disassociating from misfolded proteins. The inhibition of PDI allowed more misfolded protein to be bound with GRP78, which in turn caused GRP78 dissociation from UPR^ER^ sensors and eventually activated UPR^ER^. This finding revealed the critical function of PDI in controlling the binding between GRP78 and misfolded proteins and in fine-tuning UPR^ER^ activation in response to ERS.

ROS level regulates cancer stemness in a wide variety of cancers [50, 51]. Although previous studies have shown that lower ROS level regulated by a branch of UPR^ER^ positive feedback loop played an essential role in stemness maintenance, the specific mechanism for crosstalk between ROS level and UPR^ER^ remains poorly understood [33, 52]. Our study revealed that mtROS was generated in mitochondria and then transported to ER to modulate PDI expression and activity. In the ER lumen, the homeostatic redox environment is required for proper protein folding and disulfide bond formation. PDI is an ER oxidoreductase that catalyzes disulfide bond formation and oxidative protein folding. The regulation of PDI by mtROS functions as a “bridge” linking mitochondrial oxidative phosphorylation to UPR^ER^ activation.

We also screened out a novel specific HIF-2α inhibitor YQ-0629. In hypoxic TME, HIF-2α proteins are stabilized and heterodimerize with ARNT to regulate related gene profile [38]. We found that YQ-0629 could interact with the PAS-B pocket of HIF-2α protein, preventing the dimerization of HIF-2α with ARNT and promoting its degradation. YQ-0629 is a highly specific inhibitor for HIF-2α which does not affect HIF-1α. Although several small molecules have been identified to selectively bind to the PAS-B domain, no study have shown the clinical value of HIF-2α inhibitor for breast cancer treatment [53–56]. In this study, we verified that YQ-0629 not only suppressed breast cancer stemness in vitro, but also synergized with PTX to inhibit tumor growth and prolong survival in vivo, highlighting the great potential of YQ-0629 for the targeted treatment of breast cancer.

In summary, we elucidated that HIF-2α remodeled stemness in breast cancer and conferred chemoresistance to BCs via SOD2-mtROS-PDI/GRP78-UPR^ER^ axis. We also developed a novel HIF-2α targeted inhibitor YQ-0629, which synergizes with PTX to suppress tumor growth in vitro and in vivo and represents a promising small molecule compound for breast cancer targeted therapy.

## Material and methods

### Reagents

Recombinant human SOD2 (Abcam, ab93946), Paclitaxel (Sigma, T7402), Doxorubicin, (Sigma, D1515), Mitoxantrone (Sigma, M2305000), Cisplatin (Sigma, P4394), Docetaxel (Sigma, 01885), Mito TEMPO (Sigma, SML0737), PDI inhibitor 16F16 (Sigma, SML0021), YQ-0629 (Topscience, chemdiv diversity discovery library), (3-Aminopropyl) triethoxysilane (sigma, A3648), Glutaraldehyde solution (sigma, G7651).

### Cell culture and lentiviral transfection

Human breast cancer cell lines MCF7 and T47D were purchased from ATCC (MCF7: ATCC cat.HTB-22, RRID: CVCL_0031; T47D: ATCC cat.HTB-133, RRID: CVCL_0553). The two cell lines have been authenticated. MCF7, MCF7 MS and T47D, T47D MS cells were cultured as reported previously by He et al. [25, 57]. For hypoxia culture, cells were incubated in the hypoxia incubator (Thermo Fisher Scientific, HERACELL 150i) at 1% O_2_. Mammosphere cells derived from breast cancer patients were also cultured in DMEM-F12 medium supplemented with 2% B27, 10 μg/l b-FGF, and 20 μg/l EGF.

Lentiviral vectors were purchased from Shanghai Genechem Co., Ltd. HIF-2α-RNAi sequence is seen in Supplementary Table 1. HIF-2α cDNA (HIF-2α-OE) was cloned into a Ubi-MCS-3FLAG-SV40-EGFP-IRES-puromycin vector. GRP78, PDI and SOD2 cDNAs (GRP78-OE, PDI-OE, SOD2-OE) were cloned into a Ubi-MCS-SV40- puromycin. MCF7, T47D, MCF7 MS, and T47D MS cells were seeded into 6-well adhesion plates or ultra-low adhesion plates (Corning) at a density of 2 × 10^5^ cells per well. The next day, lentiviral vectors and polybrene were mixed with medium (NC, MOI = 10; MOI = 20, HIF-2α-OE, GRP78-OE, PDI-OE, SOD2-OE; shCtrl, MOI = 20; shHIF-2α, MOI = 25). After transfection for 12 h, fresh culture medium was added, and culturing for another 48 h. Puromycin (2 μg/ml) was added to select stably transfected cells. This process was repeated 2 to 3 times until all cells expressed green fluorescent protein (GFP).

### RNA isolation and real-time PCR

RNA isolation and real-time PCR were performed as described previously by Ma et al. [58]. Primer sequences are shown in Supplementary Table 2.

### Immunofluorescence and misfolded protein detection

Paraffin-embedded and OCT-embedded samples were sectioned at 4 μm thickness. Antigen retrieval was performed by a pressure cooker for 10 min in 0.01 citrate buffer (pH 6.0) to remove aldehyde links formed during initial fixation of tissues (this step is just for paraffin-embedded samples, skip for OCT-embedded fresh tissue samples). Cells for immunofluorescence were fixed with 4% paraformaldehyde for 20 min at room temperature, washed with PBS and permeabilized with 0.2% Triton X-100 in PBS for 15 min. Thereafter, cells were blocked in PBS with the Normal Goat Serum (Solarbio) for 30 min at 37 °C. Then, samples were incubated with primary antibodies HIF-2α, HIF-1α, PDI, GRP78 (Seen in Supplementary Table 3) overnight at 4 °C. Incubation of Alexa Fluor-conjugated secondary antibodies (EarthOx Life Sciences) were carried out for 30 min at 37 °C protected from light. Misfolded protein was detected with the PROTEOSTAT^®^ Aggresome Detection Kit (ENZ-51035; Enzo Life Sciences, USA) according to the manufacturer’s instructions. DAPI was used counterstaining the nuclei, ER-tracker^TM^ was used for staining the ER (ThermoFisher, E12353), CellMask^TM^ was used for staining the plasma membranes (ThermoFisher, C10046), MitoGreen was used for staining the mitochondria (KeyGEN BioTECH, KGMP0072) and MitoSOXTM (Thermo Fisher Scientific, m36008) was used for staining mtROS in cells. Images were obtained by laser scanning confocal microscopy (Nikon ECLIPSE Ti).

### Western blot analysis

Cells were harvested in RIPA lysis buffer (50 mM Tris-HCI; pH 7.5, 120 mM NaCl, 0,5% NP-40, 200 mM Na3VO4, 1 mM EDTA, 0.5% sodium deoxycholate, 1% SDS) containing 1 × protease inhibitor cocktail (Sigma) for 10 min on ice. An equal amount of protein from the cell lysates was resuspended in the gel sample buffer, resolved by SDS-PAGE, and transferred to PVDF membrane (Millipore). After blocking with 5% BSA, membranes were incubated with specific primary antibodies (Supplementary Table 3) overnight at 4 °C. After washing with TBST, corresponding secondary antibodies were incubated at room temperature for 1 h. The immunoreactivity of the signals was visualized by using ECL system (DNR MicroChemi system). The uncropped western blots can be seen in the Supplementary Material.

### Cell viability assay

Cell viability assay was performed as reported previously [25] by first seeding MCF7, T47D cells (2000 cells/well), or MCF7 MS, T47D MS cells (5000 cells/well) into a 96-well cell culture plate for overnight. Indicated concentrations of Paclitaxel (PTX), adriamycin (ADR), mitoxantrone (MX) and cisplatin (DDP), YQ-0629 or vehicle (DMSO) alone were then added to the wells in 100 μl DMEM or DMEM-F12 medium. The calculation formula of synergistic index (R) is R = (S-exp)/(S-obs). S-exp referred to the product of cell viability rate after the two single drugs respectively acted on cells, and it’s expressed by the formula S-exp = (S-EA) × (S-EB). S-obs referred to the cell survival rate after the two drugs acted on cells in combination. R > 1.0 indicated that the two drugs had a synergistic effect, and R < 1.0 indicated that the two drugs had no synergistic effect.

### Colony formation assay

For soft agar colony formation assay, 6-well plates were coated with a bottom layer of 1.2% SeaPlaque low melting temperature agarose (Lonza Rockland, ME USA) in phenol red-free medium supplemented with 20% FBS. 2000 cells were mixed in 0.6% agarose and the same medium, and applied as the top agarose layer. The top agarose layer was overlaid with 600 μl medium. Plates were incubated at 37 °C in 5% CO_2_ for 3 weeks until colonies formed. Colonies were stained with 100 μl MTT (5 mg/ml) in each well and incubated for 30 min at 37 °C. Colonies were calculated using the analysis software Quantity One (BioRad, Herculed, California, USA).

For plate colony formation assay, 4000 cells were plated into each well of six well plates and grown for 14 days. At the end of the experiment, cell colonies were fixed with 4% paraformaldehyde, and stained with 0.5% crystal violet (Sigma–Aldrich 46364). The number of colonies was counted under a Nikon eclipse TE2000-U microscope with pictures taken. The assay was performed three times in triplicate.

### Sphere formation assay

After treated with indicated concentrations of PTX, Mito TEMPO, PDI inhibitor 16F16, or YQ-0629 for 48 h, MCF7 MS, T47D MS or Patient MS cells (2000 cells/ml) were cultured in ultra-low adhesion plates (Corning) in DMEM-F12 medium containing 2% B27, 10 μg/l b-FGF, 20 μg/l EGF. After culturing for 14 days, spheres with diameter >150 μm were counted. At least three independent experiments were conducted.

### Flow cytometry

For cell surface markers analysis, cells were resuspended in PBS and stained with fluorescent-conjugated antibodies against CD44 (Biolegend, 103011) and CD24 (BD, 311106) for 30 min at 37 °C in darkness. For ALDH1^+^ detection, the treatment of cells was followed the manual protocol (STEMCELL, #01700). For the mtROS detection, resuspended cells were stained with 5 μM MitoSOXTM (Thermo Fisher Scientific, m36008) and reagent working solution at 37 °C for 30 min in darkness. Specimens were subsequently analyzed by MACSQuatTM Flow cytometer (Becton-Dickinson, USA).

### Coimmunoprecipitation assay

Cells were harvested and lysed in the lysis buffer on ice for 30 min. After centrifugation at 4 °C at 12,000 × *g* for 10 min, GRP78 (Supplementary Table 3) antibodies were added to the supernatant with rolling at 4 °C overnight. Protein G or A agarose (Beyotime Biotechnology) was then added to samples, and samples were rolled at 4 °C for 2 h. After beads were washed three times with lysis buffer, pellets were dissolved into 2 × SDS loading buffer. After centrifugation and boiled at 100 °C for 10 min, proteins were analyzed by immunoblotting with the PDI, PERK and GRP78 antibodies (Supplementary Table 3).

### Patients and tissue samples

Tumor samples were taken from 110 patients with breast carcinoma who underwent neither radiation nor chemotherapy prior to surgery at the First Affiliated Hospital of China Medical University, Liaoning Province, China between 2006 and 2008. According to the pathological staining, cancer was diagnosed. The patient age, menopausal status, tumor type, tumor size and lymph node metastasis were acquired from clinical records. All samples were collected from patients with informed consent, and all related procedures were performed with the approval of the internal review and ethics boards of the indicated hospitals.

### Immunohistochemistry (IHC)

Immunohistochemistry was carried out as previously described [25] Paraffin-embedded sections (4 μm) from breast cancer were dewaxed and dehydrated, and antigen retrieval was performed by high pressure sections in Citrate Antigen Unmasking Solution (Beyotime, P0081) for 10 min. After blocked with the Normal Goat Serum (Solarbio), sections were incubated overnight at 4 °C using primary antibodies (shown in Supplementary Table 3). Following incubation with a biotinylated secondary antibody (anti-rabbit, 1:200; Vector Laboratories) for 30 min at 37 °C, antigens were revealed with 3,3’-diaminobenzidine (Solarbio).

### Mouse xenografts

BALB/c (nu/nu) mice (Hua Fukang Biological Technologies Inc, Beijing) at 4–6 weeks of age were bred in pathogen-free conditions at the Animal Center of China Medical University. The mice were grouped according to their body weight to snake-shaped grouping method. The investigator was not blinded to the group.

For the study of tumorigenic abilities of MCF7 and MCF7 MS cells, different quantity of cells was suspended in PBS and Matrigel (200 μl, 1:1, BD Biosciences). Then the cell mixture was subcutaneously injected into the right flank of nude mice (*n* = 5/group). Tumor volume (V) was monitored with digital calipers using the following formula: Width^2^ × Length/2. After 31 days, the mice were euthanized, and the xenograft tumors were excised for the study.

For the study of effects of HIF-2α knockdown on sensitivities of xenograft tumors to PTX, equal numbers (1 × 10^5^) of suspended stably sh-Ctrl-transfected MCF7 MS, or sh-HIF-2α-transfected MCF7 MS cells were injected subcutaneously into the mammary fat pad of nude mice. When tumor volumes reached over 125 mm^3^, xenograft mice were randomly divided into four groups: sh-Ctrl alone, sh-Ctrl + PTX, sh-HIF-2α alone, sh-HIF-2α + PTX groups (*n* = 10/group). The mice in sh-Ctrl + PTX and sh-HIF-2α + PTX groups were intraperitoneally injected with PTX (5 mg/kg), and mice in sh-Ctrl and sh-HIF-2α groups were intraperitoneally injected with PEG-35 castor oil as control once every other day. After 31 days inoculation, some of them (*n* = 5/group) were sacrificed and tumors were weighted and harvested for further test. Before sacrificed, mice were anesthetized with chloral hydrate then photographed using Living Image software (Perkin-Elmer). And the survival of other mice (*n* = 5/group) was observed till to the 120th day.

### Patient derived xenogtaft (PDX) experiments

To establish patient-derived xenografts (PDX), primary tumor specimens were collected from breast cancer patients who underwent tumor resection at the First Affiliated Hospital of China Medical University (Shenyang, China) between 2016 and 2019. Clinical features of patients were provided in Supplementary Table 4. Eight-week-old NOD-SCID mice under pathogen-free conditions were used for PDX transplantation. Briefly, a small incision was made on the abdomen of anaesthetized NOD-SCID mice to reveal the mammary gland, and primary breast tumor samples were minced into 1–2 mm^3^ sized fragments and injected directly into the fourth pair of mammary fat pads of the mice. The incision was then closed with sutures. The time from cancer samples collection to mice implantation ranges from 1–3 h. The tumor formation was monitored in the next 3–4 months since implantation. After PDX transplant succeeded, xenografted tumors were minced into 1–2 mm^3^ sized fragments. Some fragments were transplanted again to generate secondary PDX, and some were digested with Collagenase/Hyaluronidase (Stem Cell Technology, 07912) overnight then added 3 ml pre-warmed 0.25% Trypsin-EDTA (Gibco, Thermo Fisher Scientific, 25200056) to digest fragments into single cell suspension. 1 × 10^7^ cell suspension was incubated with CD44 Microbeads (Miltenyi Biotec, 130–095–194) and CD24 MicroBeads Kit (Miltenyi Biotec, 130–0950951) to separate CD44^+^CD24^−^ BCs. Amplified CD44^+^CD24^−^ BC cells in MammoCultTM medium (Stem Cell Technology, 05620) with 10% FBS (Stem Cell Technology, 05620).

For the study of effects of YQ-0629 on sensitivities of the PDX to PTX, equal numbers (1 × 10^5^) of suspended patient MS cells were injected subcutaneously into the mammary fat pad of NOD-SCID mice. The mice were grouped according to their body weight to snake-shaped grouping method. The investigator was nor blinded to the group. When tumor volumes reached over 125 mm^3^, the PDX mice were randomly divided into four groups: Control, PTX, YQ-0629, PTX + YQ-0629 groups (*n* = 11/group). The mice in PTX group were intraperitoneally injected with PTX (5 mg/kg), the mice in YQ-0629 group were intraperitoneally injected with YQ-0629 (100 mg/kg), the mice in PTX + YQ-0629 group were intraperitoneally injected with PTX (5 mg/kg) plus YQ-0629 (100 mg/kg), and mice in Control group were intraperitoneally injected with PEG-35 castor oil once every other day. After 31 days inoculation, some of them (*n* = 6/group) were sacrificed and tumors were weighted and harvested for further test. And the survival of other mice (*n* = 5/group) was observed till to 120th day. All above animal studies were approved by Animal Research Committee at China Medical University.

### High-performance liquid chromatography–tandem mass spectrometry (HPLC-MS)

The procedures were performed by our previous reports [45]. Cells and tissues were prepared by removing proteins through a liquid-liquid extraction method. Cells (1 × 10^7^) or xenografts tissues (0.1–0.2 g) were added 500 μl methanol and fully homogenized. After centrifuging at 1000 rpm for 10 min, keeping the supernatant, 10 μl docetaxel (1 μg/ml, Sigma 1224562), as an internal standard (IS), 50 μl NaHCO3 saturated solution, and 1 ml tertbutyl methyl ether mixture was added and fully mixed. Then the mixture solution was centrifugated at 1 × 10^4 ^rpm for 10 min, and the upper organic phase was kept and evaporated to dryness at 4 °C. The dry residue was dissolved in 100 ul mobile phase with vortex-mixing for 1 min, then the reconstituted extract was taken and centrifugated at 13,000 rpm for 10 min at 4 °C, and 10 ul of supernatant fluid was kept and injected into the HPLC-MS/MS system for analysis. Controls and samples were analyzed on a 3500 MS/MS system from Applied AB Sciex (Ontario, Canada) coupled to an Agilent HPLC 1290 system (Agilent, Santa Clara, CA, USA). Separations were accomplished on an Agilent ZORBAX Eclipse Plus C 18 (2.1 mm × 100 mm, 1.8 µm) with an Agilent guard cartridge at temperature of 30 °C. The mobile phase consisted of methanol and 0.1% formic acid water (70:30) which was delivered at a flow rate of 0.3 ml/min. The injection volume was 5 µl. The mass spectrometer was operated in a positive ion mode with a TurboIonSpray source using ESI ionization in MRM mode.

### Computation of protein–protein docking

The structural models of HIF-2α-PAS domain (4XT2), HIF-1α-PAS domain (4H6J), GRP78 polypeptide-binding pocket (6ASY), reduced PDI (4EKZ), oxidized PDI (4EL1) were downloaded from the Protein Data Bank (http://www.rcsb.org/pdb/home/home.do). The chemical library was from chemdiv diversity discovery library (Topscience Co., Ltd). The docking model between GRP78 and PDI was performed by MOE2016 software (Chemical Computing Group Inc).

### PDI assay

PDI activity was assayed by measuring the PDI-catalyzed reduction of insulin in the presence of DTT, thus measuring the aggregation of reduced insulin B chains at 620 nm. Briefly, total protein extracts of different samples were treated according to present manual [59].

### Biacore assay

HIF-2α was immobilized on an NTA Sensor Chip in Biacore T200 (GE Healthcare). Binding of HIF-2α to YQ-0629 was analyzed at 25 °C in PBS-P or PBS-P with 2% DMSO at pH7.4 buffer with a flow rate of 30 µg/ml min or 20 µg/ml min. The kinetics and dissociation constant (KD) were calculated with Biacore T200 Evaluation Software (GE Healthcare).

### TCGA data processing

Gene’s expression analyses were based on the The Cancer Genome Altas (TCGA) Reasearch Nerwork: http://cancergenome.nih.gov/. In TCGA, total breast cancer patients were 1169 cases. The positive criteria of HIF-2α, HIF1α, P-gp, and BCRP, CD44^+^CD24^-^ were based on the ROC curve.

### Statistical analysis

Statistical analyses were conducted in GraphPad Prism 7 (RRID: SCR_002798). Results were p34resented as the mean ± standard deviation (SD) for at least three experiments. Student’s *t* test was used to compare differences between two groups. When the variance of each group is not uniform, Wilcoxon or Welch“s T-est can be used for analysis. One-way ANOVA or two-way ANOVA was used to compare differences among three or more groups. Mann–Whitney *U* analysis was used to compare mRNA expressions of HIF-2α or HIF1α in CD44^+^CD24^−^ and non-CD44^+^CD24^−^ breast cancer patients from TCGA database, and protein levels of HIF-2α or HIF-1α in CD44^+^CD24^−^ and non- CD44^+^CD24^−^ breast cancer patients from our tissue bank. Pearson χ2 test was used to analyze correlations between HIF-2α or HIF1α and CD44^+^CD24^−^ mRNA level, or between HIF-2α or HIF-1α and CD44^+^CD24^−^ protein level. A *P* < 0.05 was considered statistically significant.

## Supplementary information

Supplemantary material

Original data

Pre-authorship

aj-checklist

## Data Availability

The authors declare that all data supporting the findings of this study are available within the paper in the main text or the Supplementary Materials.

## Materials availability

All data on which the conclusions of the paper rely are available to readers.
