# Supplementary material for: A novel HIF-2α targeted inhibitor suppresses hypoxia-induced breast cancer stemness via SOD2-mtROS-PDI/GPR78-UPRER axis
Source: Cell Death Differ. 2022 Mar 17;29(9):1769–89. doi: 10.1038/s41418-022-00963-8 (PMC9433403; doi:10.1038/s41418-022-00963-8)
Supplement: Supplementary file 1 — Supplemantary material [file 41418_2022_963_MOESM1_ESM.docx]

**Supplementary material**

**
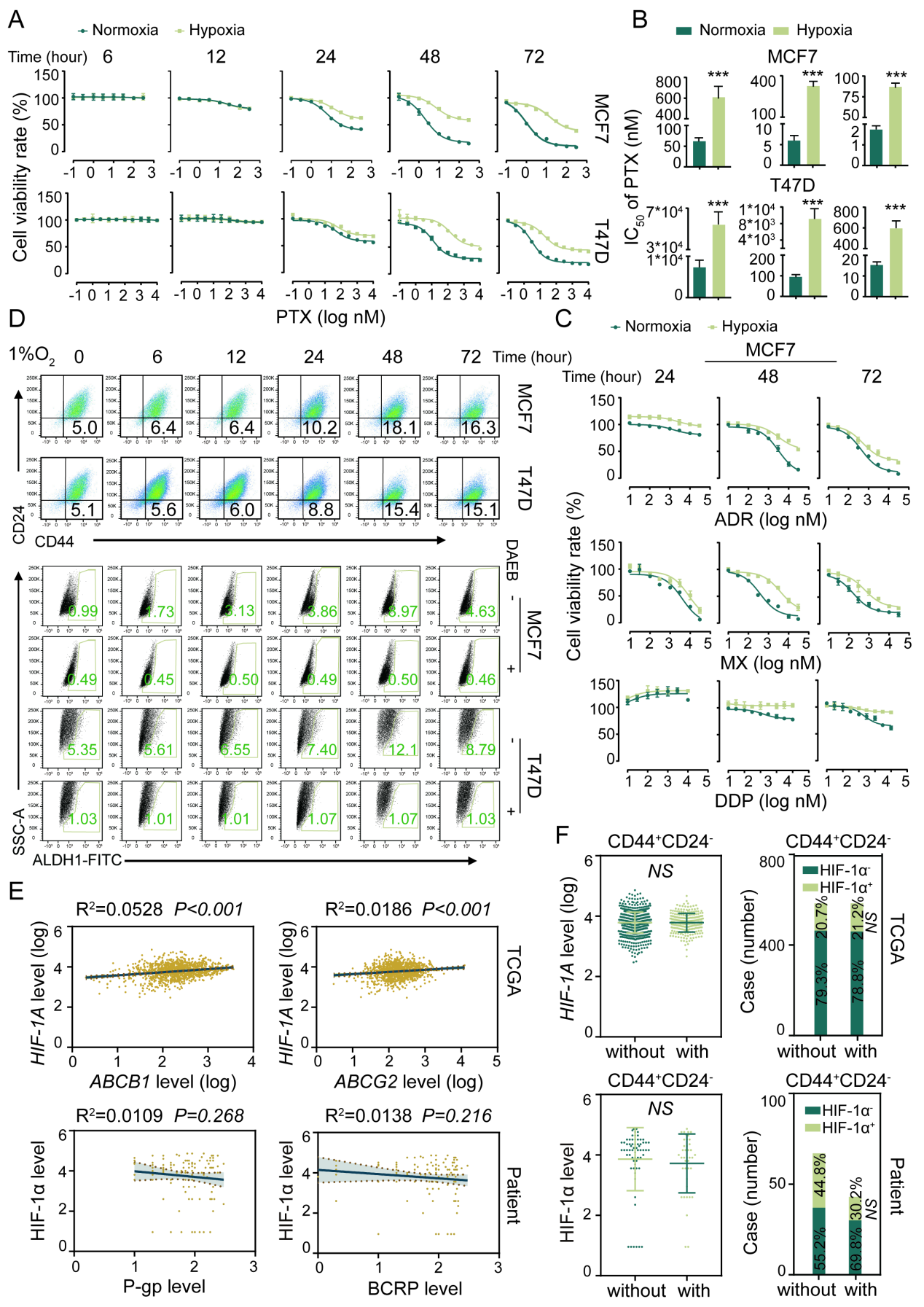
**

**Figure S1, related to Figure 1. Hypoxia-induced HIF-2α upregulation promotes stemness and chemoresistance acquisition**

1. The cell viability rate was detected in MCF7 (up panel) and T47D (down panel) cells cultured with different concentrations of paclitaxel (PTX) under hypoxia for 0-72 hours by MTT assay. The green color represents normoxia (20% O_2_), the light green color represents hypoxia (1% O_2_).
2. The IC_50_ values of PTX were calculated cultured with MCF7 and T47D cells for 0-72 hours.
3. The cell viability rate was detected in MCF7 cells cultured with different concentrations of adriamycin (ADR), mitoxantrone (MX) and cisplatin (DDP) for 24-72 hours by MTT assay.
4. The proportion of CD44^+^CD24^-^ and ALDH1^+^ subpopulation in MCF7 and T47D cells were detected by flow cytometry under hypoxia for 0-72 hours.
5. The correlations were analyzed among HIF-1α, P-gp, and BCRP in mRNA and protein levels from TCGA database (n=1169) and our sample’s bank (n=110).
6. The mRNA and protein levels of HIF-1α (HIF*1A*) was compared in CD44^+^CD24^-^ and non-CD44^+^CD24^-^ patients from TCGA dataset (n=1169) and our sample’s bank (n=110) (left panel). The right panel displays correlations between HIF-1α^+^ and CD44^+^CD24^-^ phenotype in mRNA and protein level.

NS, non-significant; ****P<0.001*, compared to normoxia; Student's t test, One-way ANOVA test, Pearson correlation analysis, Mann-Whitney U analysis, Pearson χ^2^ test. Error bars, mean ± SD (n=3).

**
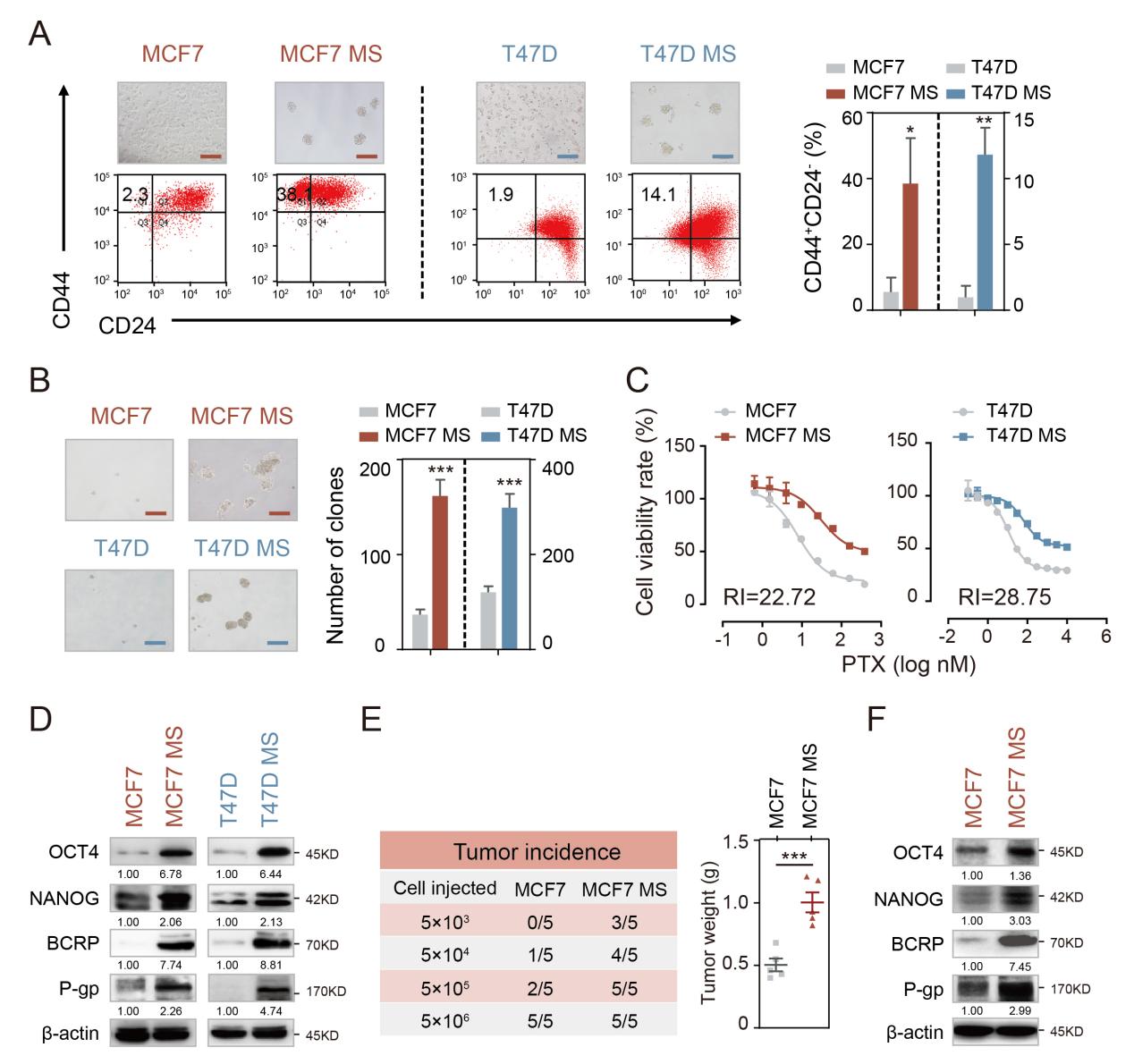
**

**Figure S2, related to Figure 2. Validation of BCSC stemness**

1. MCF7 and T47D cells cultured in sphere culture medium formed tight sphere-like mammospheres (left up panel). The percentage of CD44^+^CD24^-^ subset in sensitive cells (MCF7, T47D) and resistant cells (MCF7 MS, T47D MS) was detected by flow cytometry (left down and right panel). Scale bar, 250 μm.
2. The self-renewal ability of sensitive cells (MCF7, T47D) and resistant cells (MCF7 MS, T47D MS) was detected by soft agar colony formation assays.
3. The cell viability rate was detected in sensitive cells (MCF7, T47D) and resistant cells (MCF7 MS, T47D MS) cultured with different concentrations of PTX for 48 hours. Resistance index (RI) values were calculated relative to sensitive cells (MCF7, T47D).
4. The protein expressions of OCT4, NANOG, BCRP and P-gp were measured in MCF7, T47D, MCF7 MS, T47D MS cells.
5. The tumor weights were measured after inoculation with 5×10^6^ MCF7 or MCF7 MS cells for 4 weeks (n=5, right panel); as well as the tumor incidence (n=5, left panel).
6. The expression levels of OCT4, NANOG, BCRP and P-gp were detected in xenografts tissue.

NS, non-significant; **P<0.05*, ***P<0.01*, compared to MCF7/T47D cells; Student's t test. Error bars, mean ± SD (n=3).

**
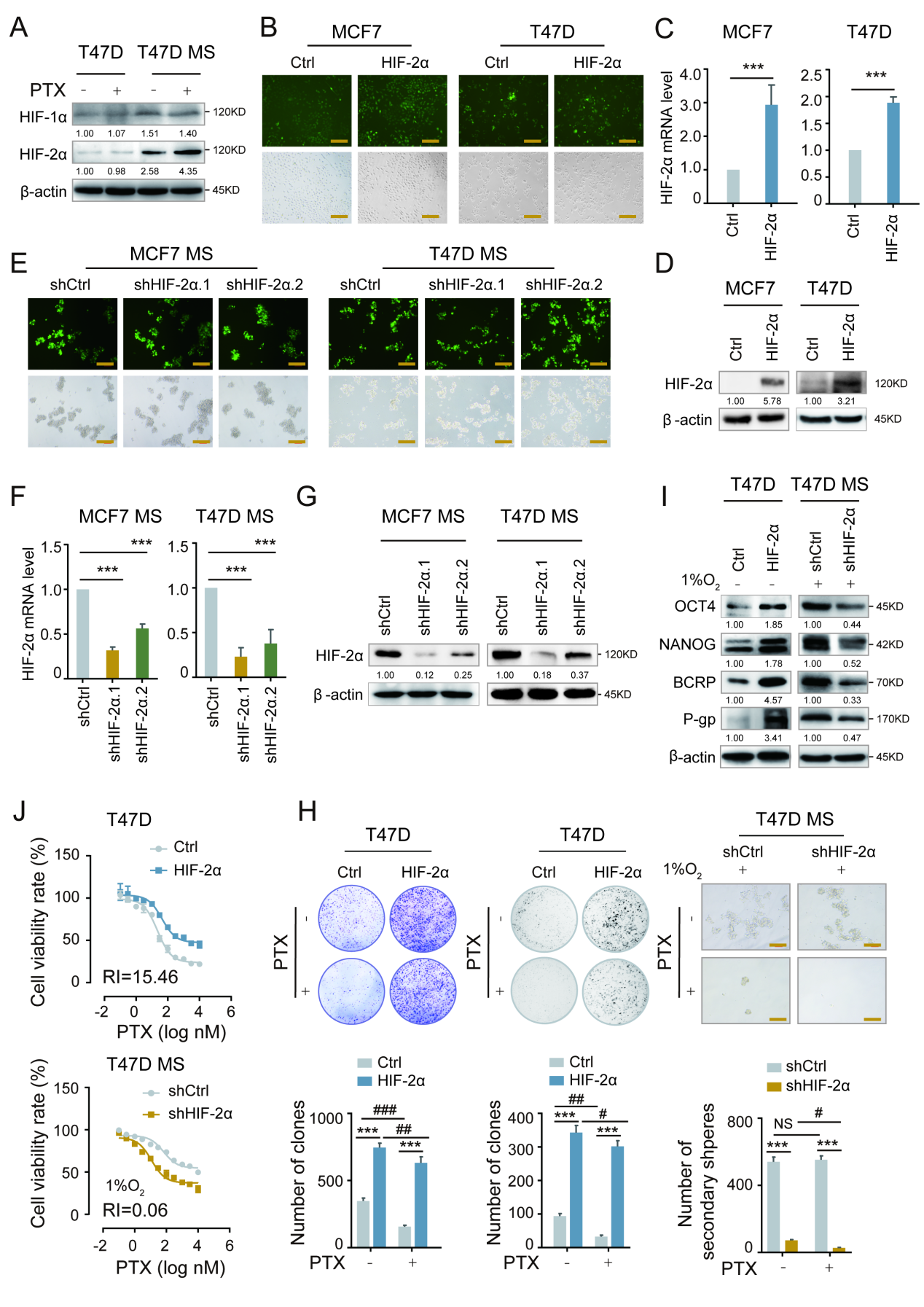
**

**Figure S3, related to Figure 2. HIF-2α maintains BCSC stemness**

1. The expression levels of HIF-1α and HIF-2α were measured in T47D and T47D MS cells cultured with or without PTX (15 nM) for 48 hours.
2. The green fluorescent protein was observed in HIF-2α-stably-overexpressing MCF7 cells under optical microscope. Scale bar, 250 μm.
3. The mRNA level of HIF-2α was detected in HIF-2α OE MCF7 and T47D cells by q-PCR.
4. The protein level of HIF-2α was detected in HIF-2α OE MCF7 and T47D cells by western blot.
5. The green fluorescent protein was observed in HIF-2α-stably-silenced MCF7 MS and T47D MS cells under optical microscope. Scale bar, 250 μm.
6. The mRNA level of HIF-2α was detected in HIF-2α KD MCF7 MS and T47D MS cells by q-PCR.
7. The protein level of HIF-2α was detected in HIF-2α KD MCF7 MS and T47D MS cells by western blot.
8. The self-renewal ability was detected in HIF-2α OE T47D cells and HIF-2α KD T47D MS cells cultured with or without PTX (15 nM) for 48 hours. Scale bar, 250 μm.
9. The expression levels of OCT4, NANOG, BCRP and P-gp were measured in HIF-2α OE T47D cells and HIF-2α KD T47D MS cells.
10. The cell viability rate was detected in HIF-2α OE T47D cells and HIF-2α KD T47D MS cells cultured with different concentrations of PTX for 48 hours. Resistance index (RI) value was calculated.

NS, non-significant; #*P<0.05*, ##*P<0.01*, ###*P<0.001*, compared to treatment without PTX **P<0.05*, ***P<0.01*, ****P<0.001*, compared to Ctrl/shCtrl/shHIF-2α; Student's t test, two-way ANOVA test. Error bars, mean ± SD (n=3).

**
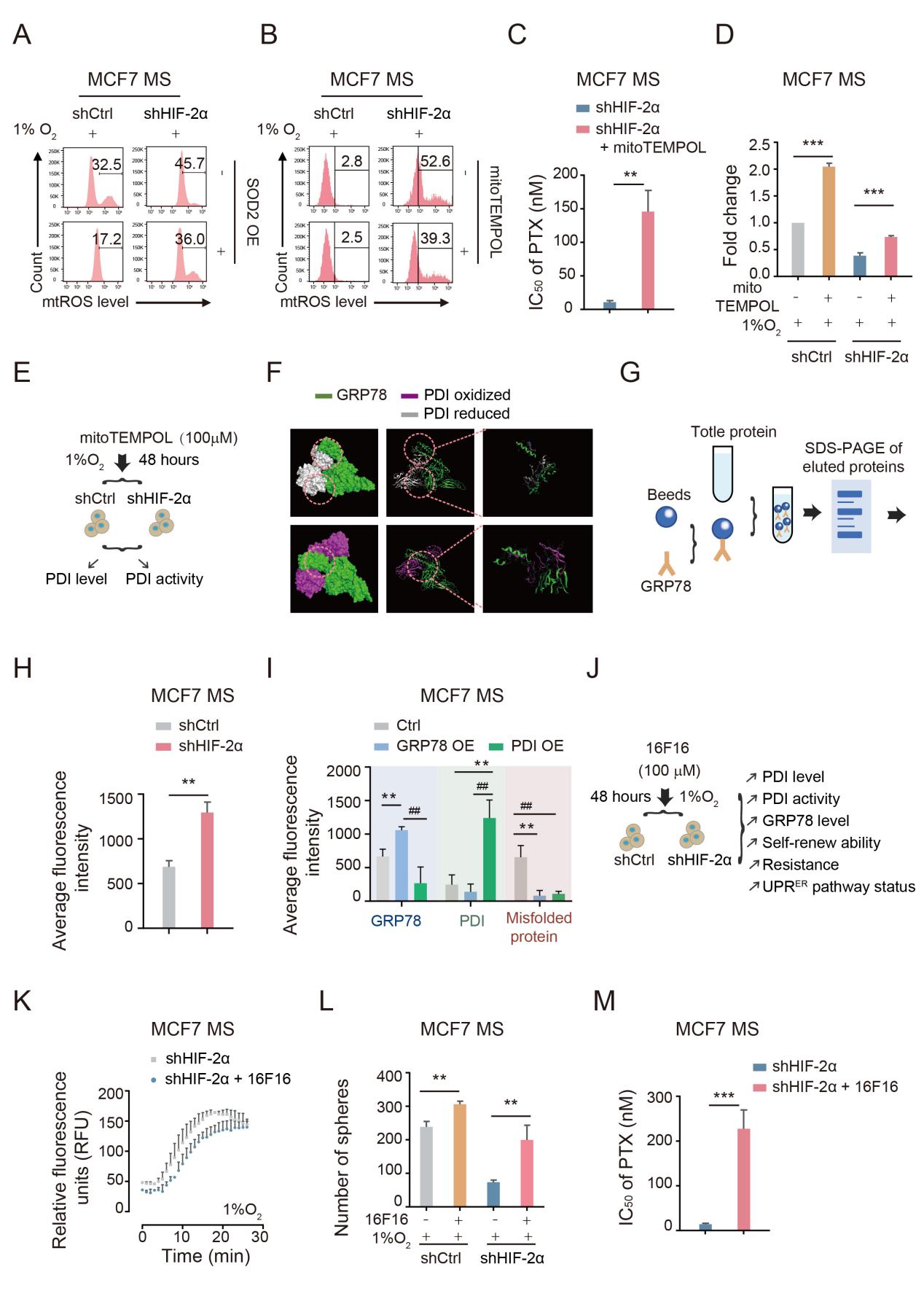
**

**Figure S4, related to Figure 3 and 4. HIF-2α promotes stemness and chemoresistance via SOD2-mtROS-PDI/GRP78-UPR^ER^ pathway**

1. The levels of mtROS were detected in SOD2-overexpressing and HIF-2α-silencing (SOD2 OE + HIF-2α KD) MCF7 MS cells, under 1% O_2_.
2. The levels of mtROS were detected in HIF-2α KD MCF7 MS cells cultured with mitoTEMPOL (100µM) for 48 hours, under 1% O_2_.
3. The IC_50_ of PTX was calculated in HIF-2α KD MCF7 MS cells cultured with mitoTEMPOL (100µM) for 48 hours, under 1% O_2_.
4. The expression level of GRP78 was detected in HIF-2α KD MCF7 MS cells cultured with mitoTEMPOL (100µM) for 48 hours by ELISA, under 1% O_2_.
5. Schematic diagram showed the mitoTEMPOL cultured process and PDI detection indicator.
6. The computational molecular docking models of active domain of GRP78 (green), reduced PDI (white) and active domain of oxidized PDI (pink, sequence: 418-633) were analyzed by MOE software.
7. Schematic diagram displayed CO-IP detection process.
8. The average fluorescence intensity of misfolded protein was measured in HIF-2α KD MCF7 MS cells.
9. The average fluorescence intensity of GRP78, PDI and misfolded protein were measured in GRP78-overexpressing MCF7 MS cells and PDI-overexpressing MCF7 MS cells.
10. Schematic diagram displayed the 16F16 (100 µM) cultured process and related detection indicator.
11. The activity of PDI was measured in HIF-2α KD MCF7 MS cells cultured with 16F16 (100 µM).
12. The number of mammospheres was counted in HIF-2α KD MCF7 MS cells cultured with 16F16.
13. The IC_50_ of PTX was calculated in HIF-2α KD MCF7 MS cells cultured with 16F16 (100 µM) for 48 hours, under 1% O_2_.

NS, non-significant; **P<0.05*, ****P<0.001*, ****P<0.001*, compared to shCtrl/shHIF-2α; Student's t test, two-way ANOVA test. Error bars, mean ± SD (n=3).

**
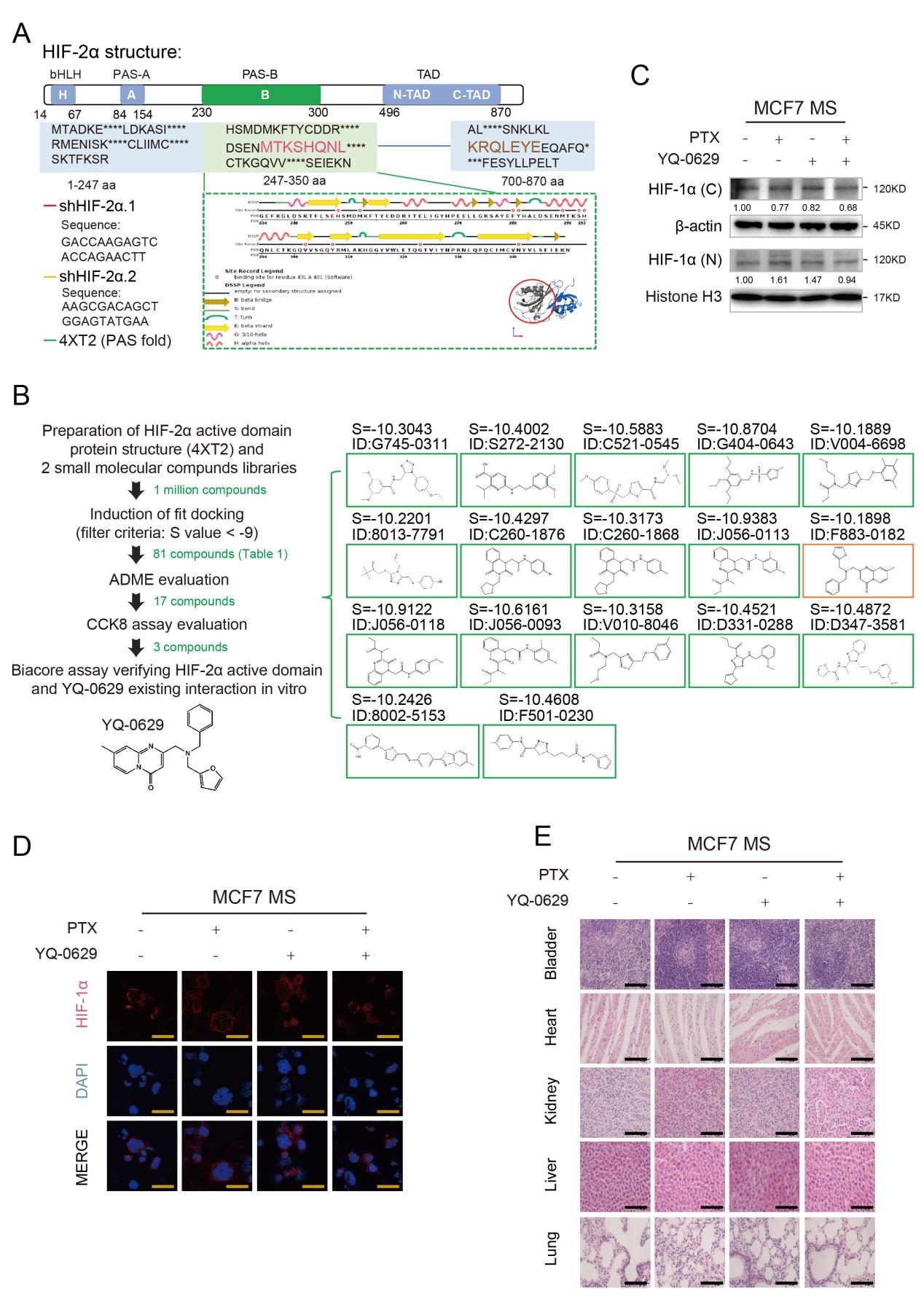
**

**Figure S5, related to Figure 6 and Figure 7. Screening strategy of YQ-0629 and its specificity/toxicity**

1. The amino acid sequence of HIF-2α. The amino acid sequence of shHIF-2α.1 is MTKSHQNL in PAS-B domains. 4XT2 is a PAS-B domain of HIF-2α including the amino acid sequences from 239 to 350.
2. The screening procedure of YQ-0629 *in vitro* and *in vivo*.
3. The protein expression of HIF-1α was detected in the nucleus (N) and cytoplasm (C) of MCF7 MS cells cultured with YQ-0629 (10 µM) alone, PTX (3 nM) alone, or YQ-0629 (10 µM) combined with PTX (3 nM) for 72 hours.
4. The expression and location of HIF-1α were detected in MCF7 MS cells cultured with YQ-0629 (10 µM) alone, PTX (3 nM) alone, or YQ-0629 (10 µM) combined with PTX (3 nM) for 72 hours by immunofluorescence staining. Scale bar, 10 μm.
5. HE staining of vital organs, including bladder, heart, kidney, liver, lung. Scale bar, 50 μm.

**Supplementary Table 1. The HIF-2α RNAi sequence**

| **HIF-2α-RNAi ID** | **RNAi sequence** | **Target protein sequence** |
| --- | --- | --- |
| sh-HIF-2α.1 | GTTCTGGTGACTCTTGGTC | MTKSHQNL |
| sh-HIF-2α.2 | GCGACAGCTGGAGTATGAA | KRQLEYE |

**Supplemental Table 2. The sequences of qRT-PCR primers**

| **Target gene** | **Primer forward sequence (5’-3’)** | **Primer reverse sequence (5’-3’)** |
| --- | --- | --- |
| HIF-2α | CTACGCCACCCAGTACCAGG | GACACCTTGTGGGCTGACG |
| HES-1 | TGAAGGATTCCAAAAATAAAATTCTCTGGG | CGCCTCTTCTCCATGATAGGCTTTGATGAC |
| SOD1 | TACTGATGGACGTGGAACCC | GAACCATCCACTTCGAGCA |
| SOD2 | GTTCAATGGTGGTGGTCATATCA | GCAACTCCCCTTTGGGTTCT |
| β-actin | TCCTCCCTGGAGAAGAGCTA | TCCTGCTTGCTGATCCACAT |

**Supplement Table 3. Antibodies used in WB, IF, IHC**

| **Target protein** | **Antibody** | **RRID** | **Application in analysis** |
| --- | --- | --- | --- |
| GRP78 | CST, #3177 | AB_10865764 | WB (1:1000); IHC (1:200); |
| HIF-1α | CST, #3716 | AB_2116962 | WB (1:500); IF (1:200) |
| HIF-2α | CST, #7096 | AB_10898028 | WB (1:500); IHC (1:200); IF (1:200) |
| p-PERK | Santa, sc-32577 | AB_2293243 | WB (1:200); |
| PERK | CST, #3192 | AB_2095847 | WB (1:200); |
| p-IRE1 | Abcam, ab48187 | AB_873899 | WB (1:500); |
| IRE1 | Abcam, ab37073 | AB_775780 | WB (1:500); |
| XBP1s | CST, #12782 | AB_2687943 | WB (1:1000); |
| AFT6 | Santa, sc-14250 | AB_671739 | WB (1:200); |
| PDI | CST, #3501 | AB_2156433 | WB (1:1000); IF (1:200) |
| BCRP | CST, #42078 | AB_2799211 | WB (1:500); |
| P-gp | CST, #13978 | AB_2798357 | WB (1:500); IHC (1:200); |
| OCT4 | CST, #2750 | AB_823583 | WB (1:1000); |
| Nanog | CST, #4903 | AB_10559205 | WB (1:1000); |
| β-actin | CST, #3700 | AB_2242334 | WB (1:8000); |
| Histone H3 | CST, #4499 | AB_90755 | WB (1:8000); |
| GRP78 | Thermo, PA5-85169 | AB_2792315 | CO-IP (1:50) |
| PERK | CST, #5683S | None | CO-IP (1:50) |

CST: Cell Signal technology; Santa: Satna Cruz Biotechnology.

**Supplement Table 4. Information of patients whose tumor tissue were used for PDX mice.**

| **Patients ID** | **Age** | **Histology grade** | **ER**  **(%)** | **PR**  **(%)** | **HER2** | **Tumor size (mm)** | **Axillary LN** | **Successfully engrafted** |
| --- | --- | --- | --- | --- | --- | --- | --- | --- |
| LA01 | 44 | II | 55 | 25 | - | 21*16.5 | Y | N |
| LA02 | 56 | III | 45 | 60 | - | 33*24 | Y | N |
| LA03 | 63 | II | 80 | 30 | - | 47*33 | Y | N |
| LA04 | 39 | II | 45 | 25 | - | 28.5*22 | N | N |
| T01 | 42 | III | 0 | 0 | - | 45*36.5 | Y | N |
| T02 | 46 | III | 0 | 0 | - | 33.5*23.5 | N | N |
| T03 | 38 | III | 0 | 0 | - | 30*30 | N | N |
| LA05 | 57 | III | 90 | 0 | - | 50*50 | Y | Y |
| H01 | 61 | III | 0 | 0 | + | 24*12 | N | N |
| LB01 | 48 | III | 90 | 20 | + | 45*32 | Y | Y |
| H03 | 55 | III | 0 | 0 | + | 26*11.5 | Y | N |
| LA06 | 47 | II | 80 | 0 | - | 35*23.5 | N | N |
| H04 | 57 | II | 0 | 0 | + | 50*50 | N | N |
| H05 | 66 | III | 0 | 0 | + | 43*38.5 | N | N |
| T04 | 51 | III | 0 | 0 | - | 28*22 | Y | N |
| T: Triple negative breast cancer; H: Her2 positive breast cancer; LA: Luminal A breast cancer; Luminal B breast cancer; Y: YES; N: NO. | | | | | | | | |
