# Supplementary material for: A novel HIF-2α targeted inhibitor suppresses hypoxia-induced breast cancer stemness via SOD2-mtROS-PDI/GPR78-UPRER axis
Source: Cell Death Differ. 2022 Mar 17;29(9):1769–89. doi: 10.1038/s41418-022-00963-8 (PMC9433403; doi:10.1038/s41418-022-00963-8)

Original data

Figure 1B_MCF7

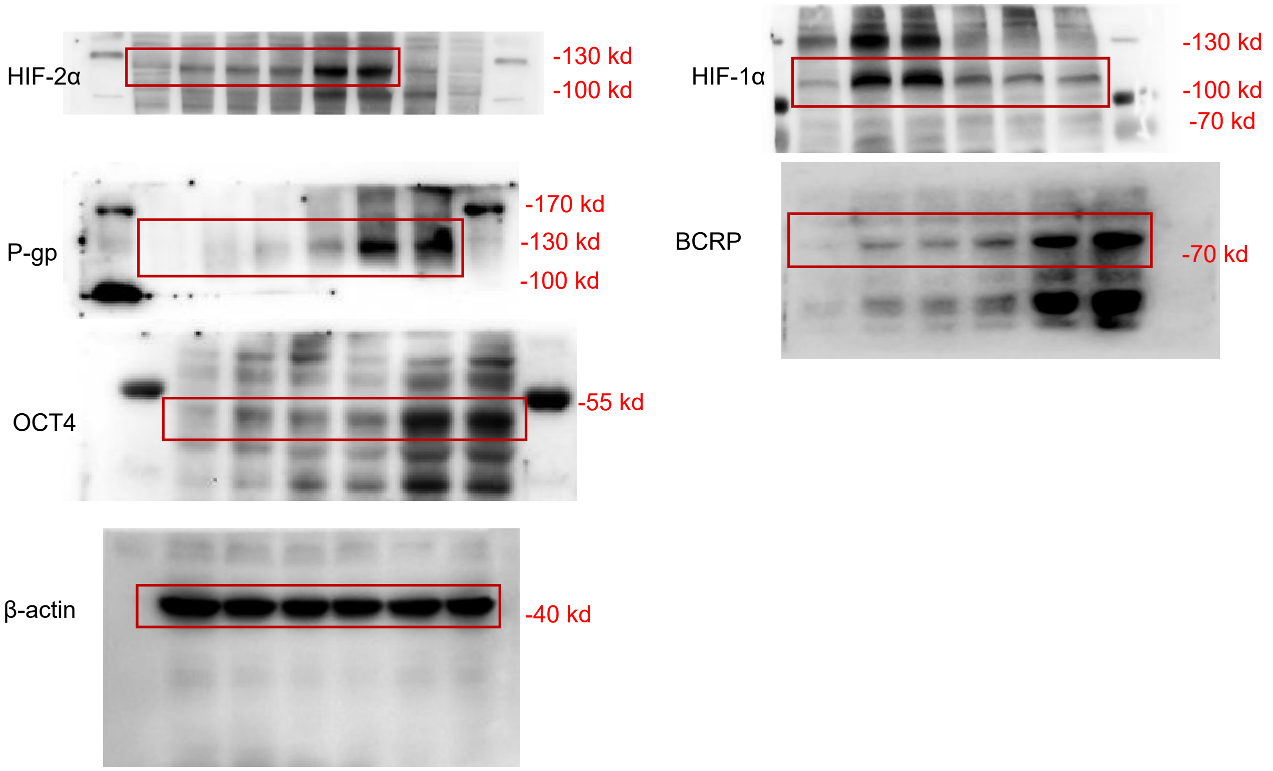

Figure 1B_T47D

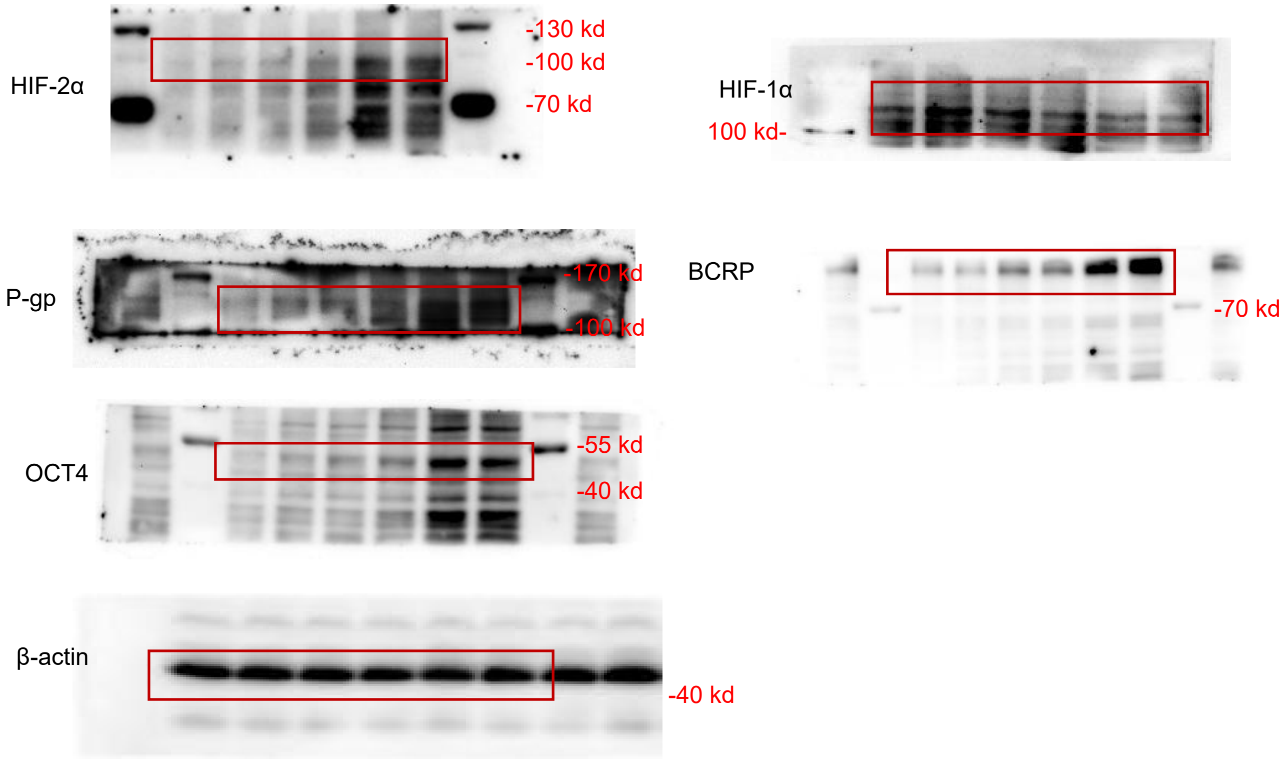

Figure 2A_MCF7 VS MCF7 MS

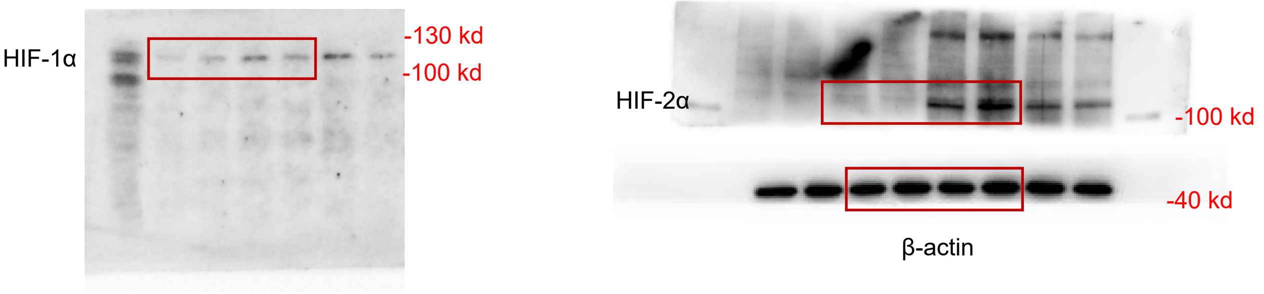

Figure 2C_MCF7_Ctrl vs HIF-2α

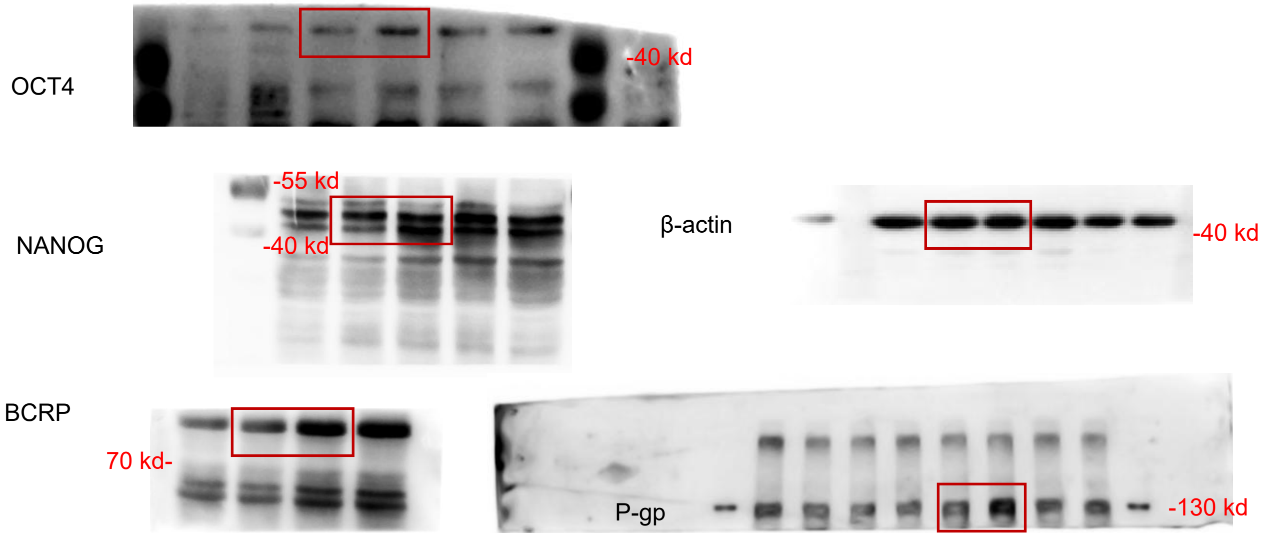

Figure 2C_MCF7 MS_shCtrl vs shHIF-2α

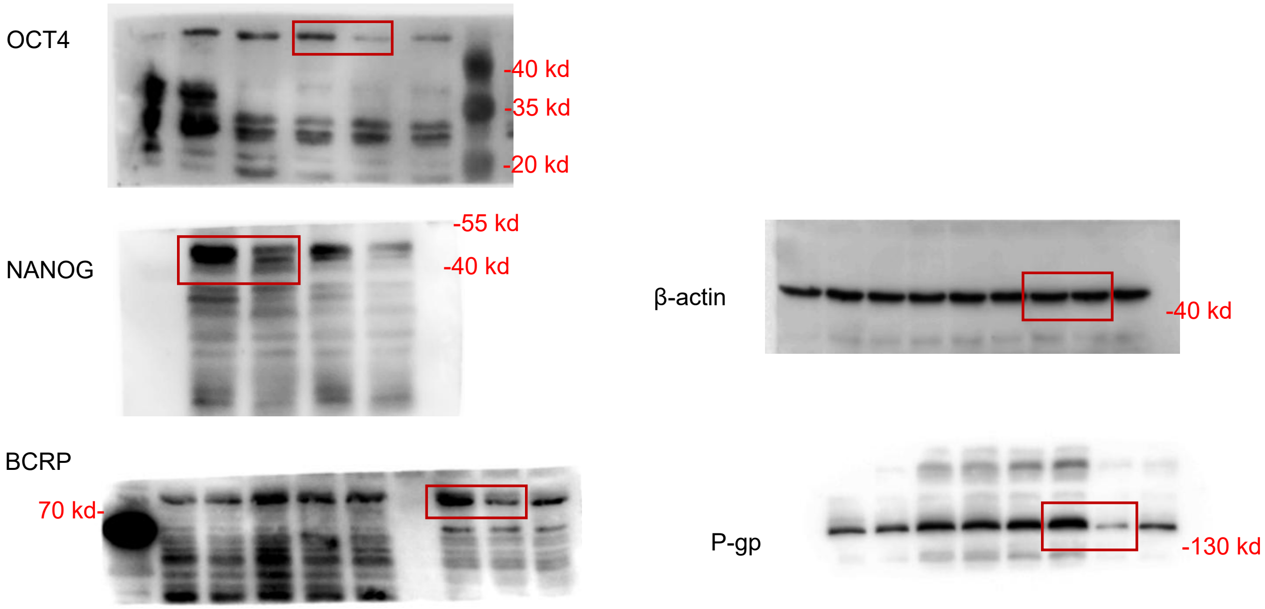

Figure 2F_MCF7 vs MCF MS

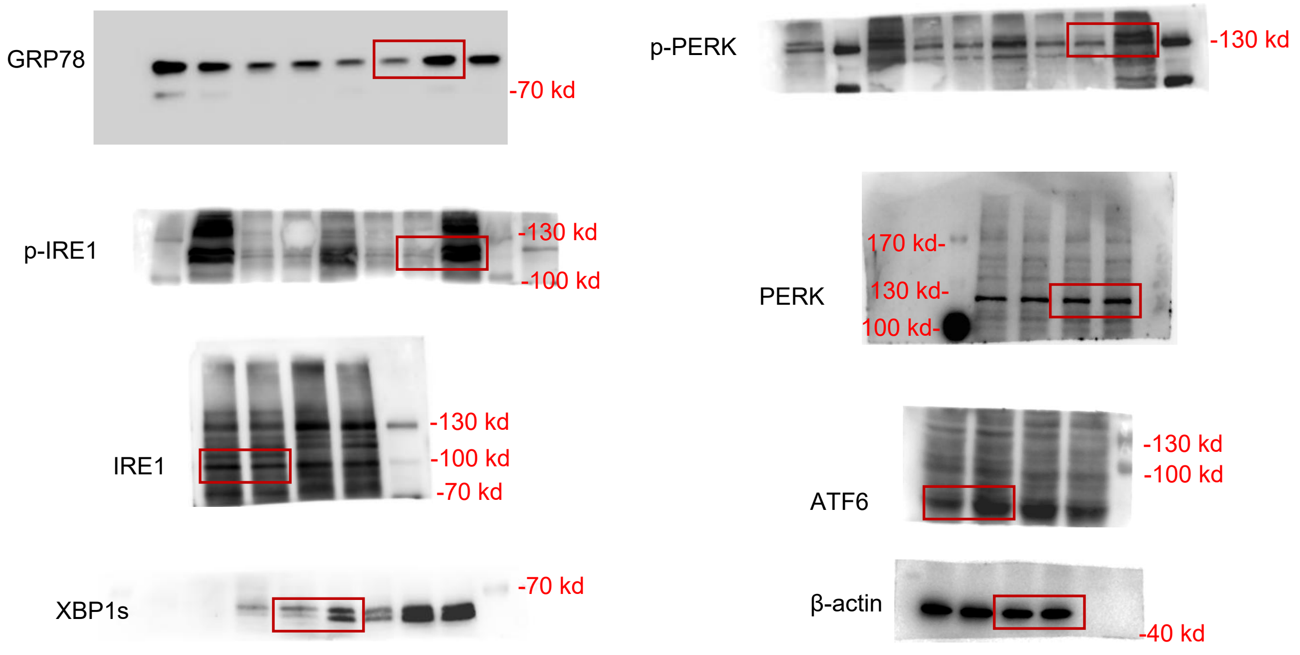

Figure 2F_MC7_Ctrl vs HIF-2α

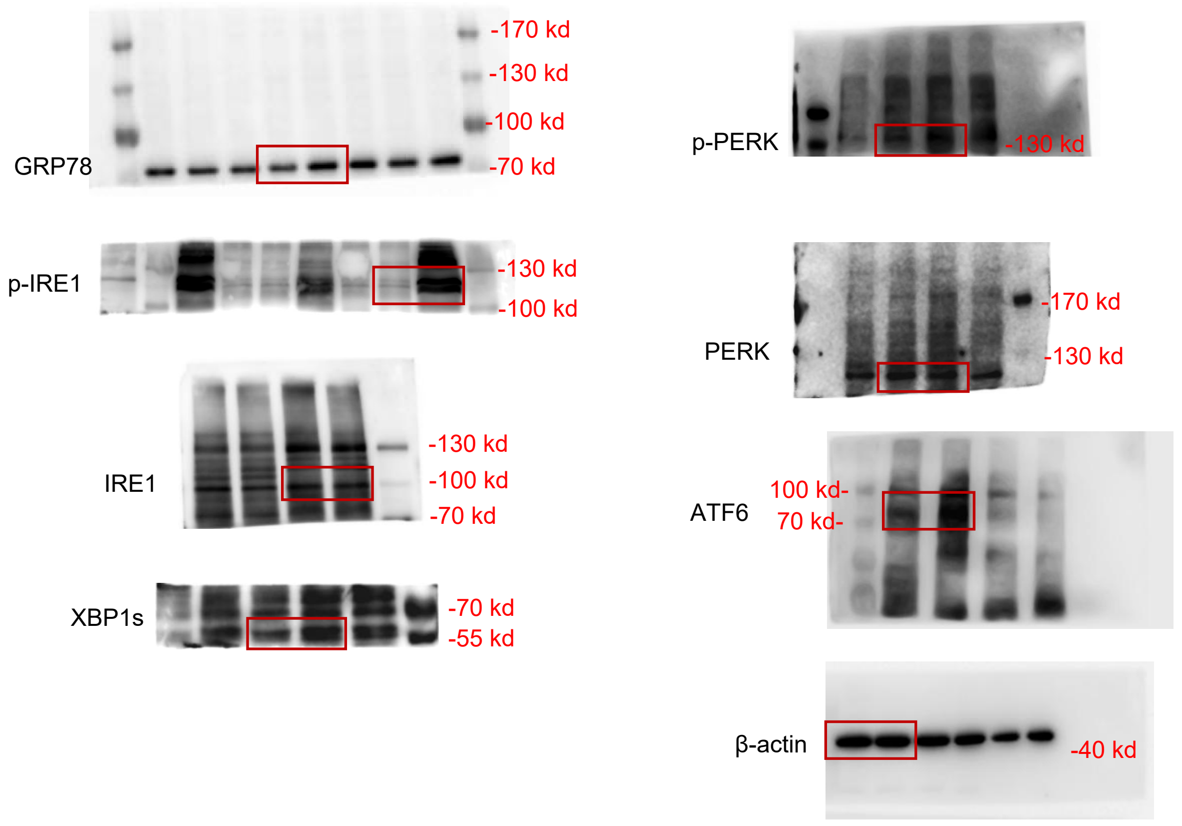

Figure 2F_MCF7 MS_shCtrl vs shHIF-2α

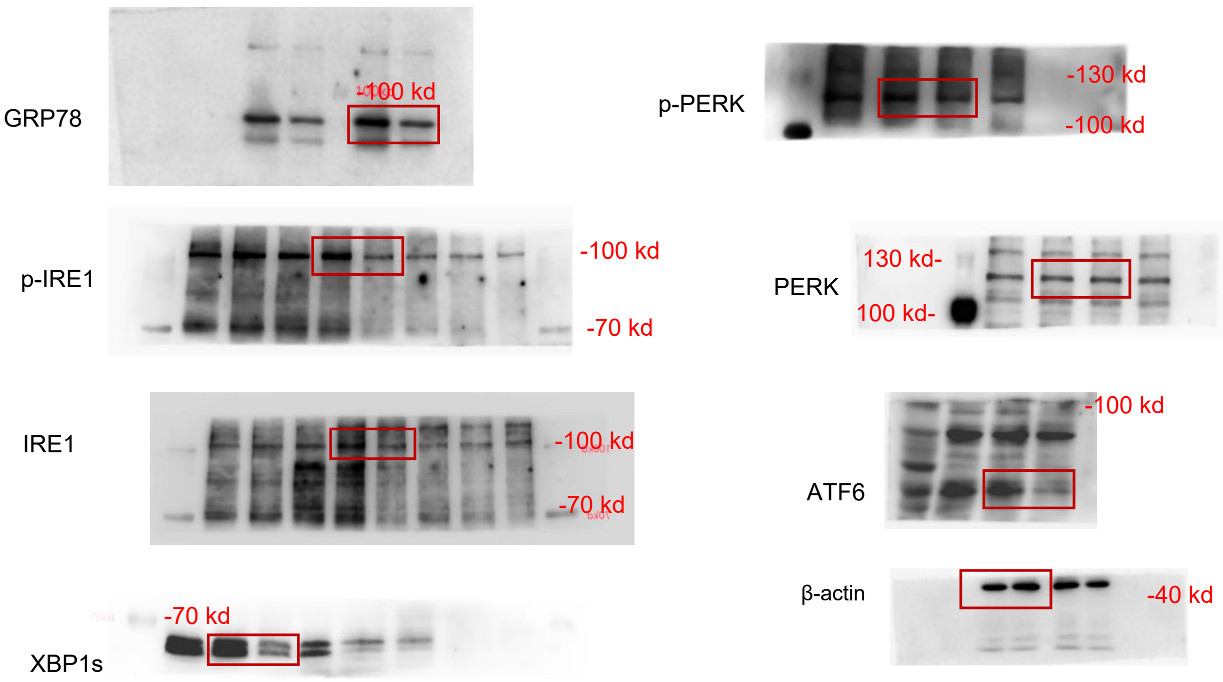

Figure 2H_MCF7 MS_shCtrl vs shHIF-2α

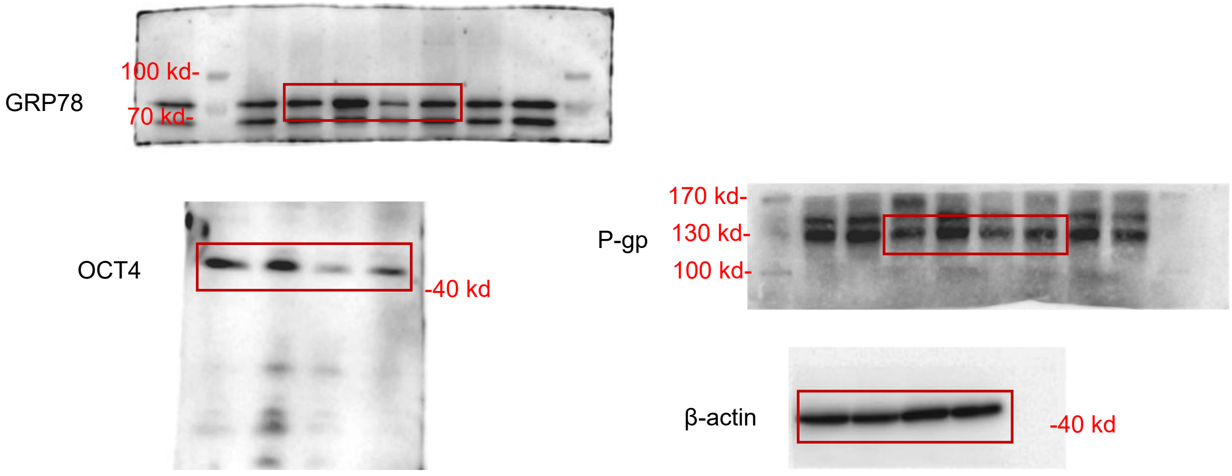

Figure 3I_MCF7 MS_shCtrl vs shHIF-2α

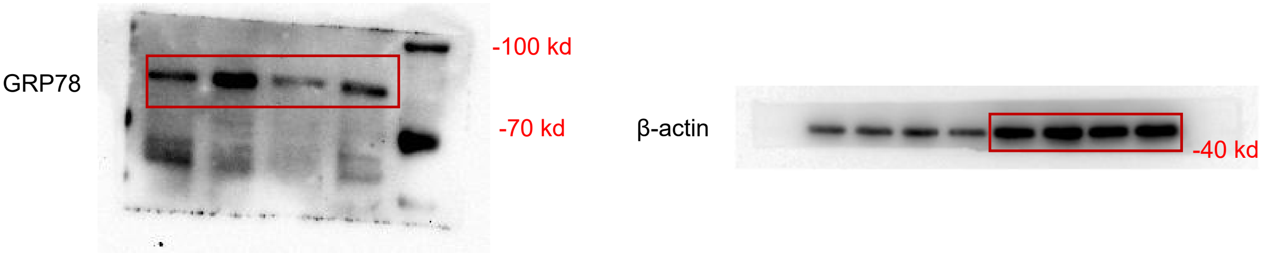

Figure 4B _MCF 7_Ctrl vs HIF-2α

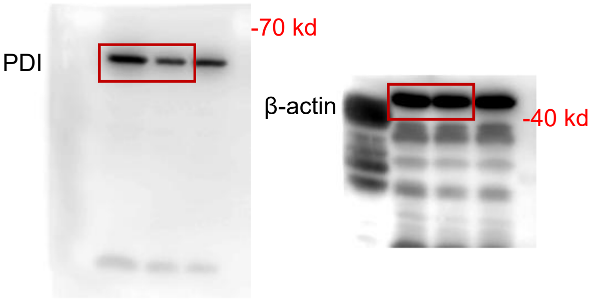

Figure 4B_MCF7 MS_sh-Ctrl vs sh-HIF-2α

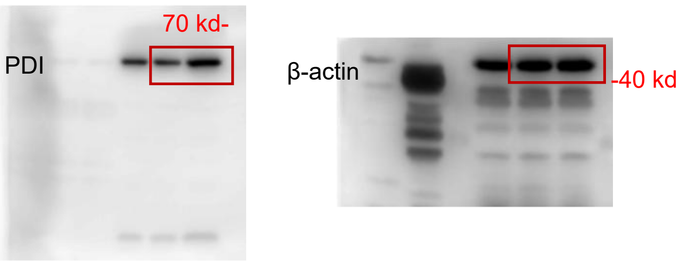

Figure 4E_MCF7 MS_sh-Ctrl vs sh-HIF-2α

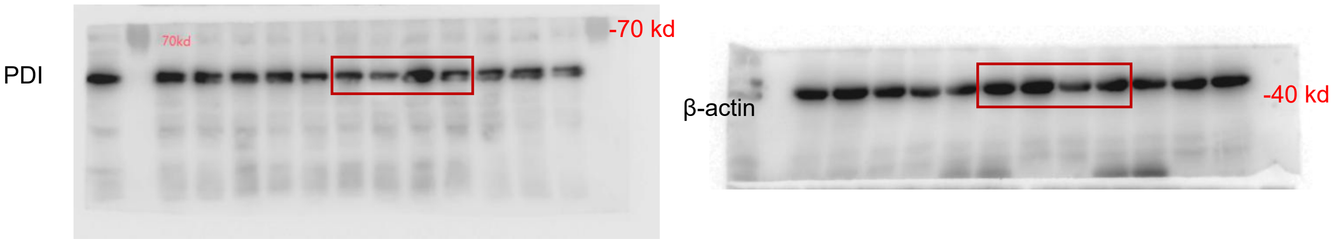

Figure 4F_MCF7 MS

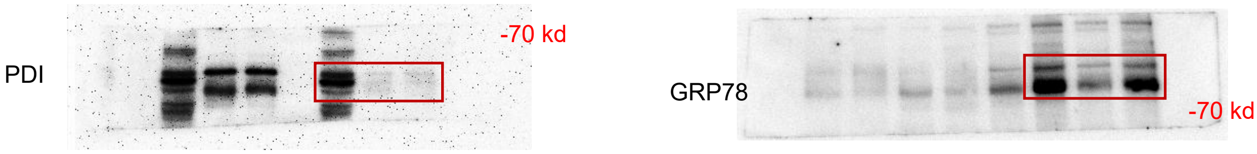

Figure 4I_MCF7 MS

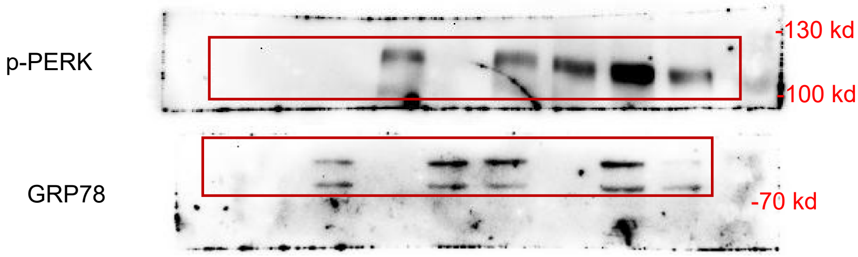

Figure 4J_MCF7 MS_sh-Ctrl vs sh-HIF-2α

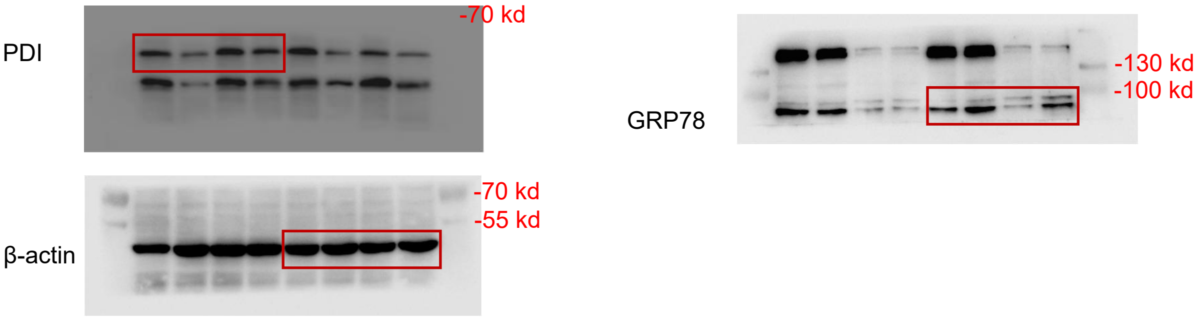

Figure 5H_MCF7 MS_xenograft tumors

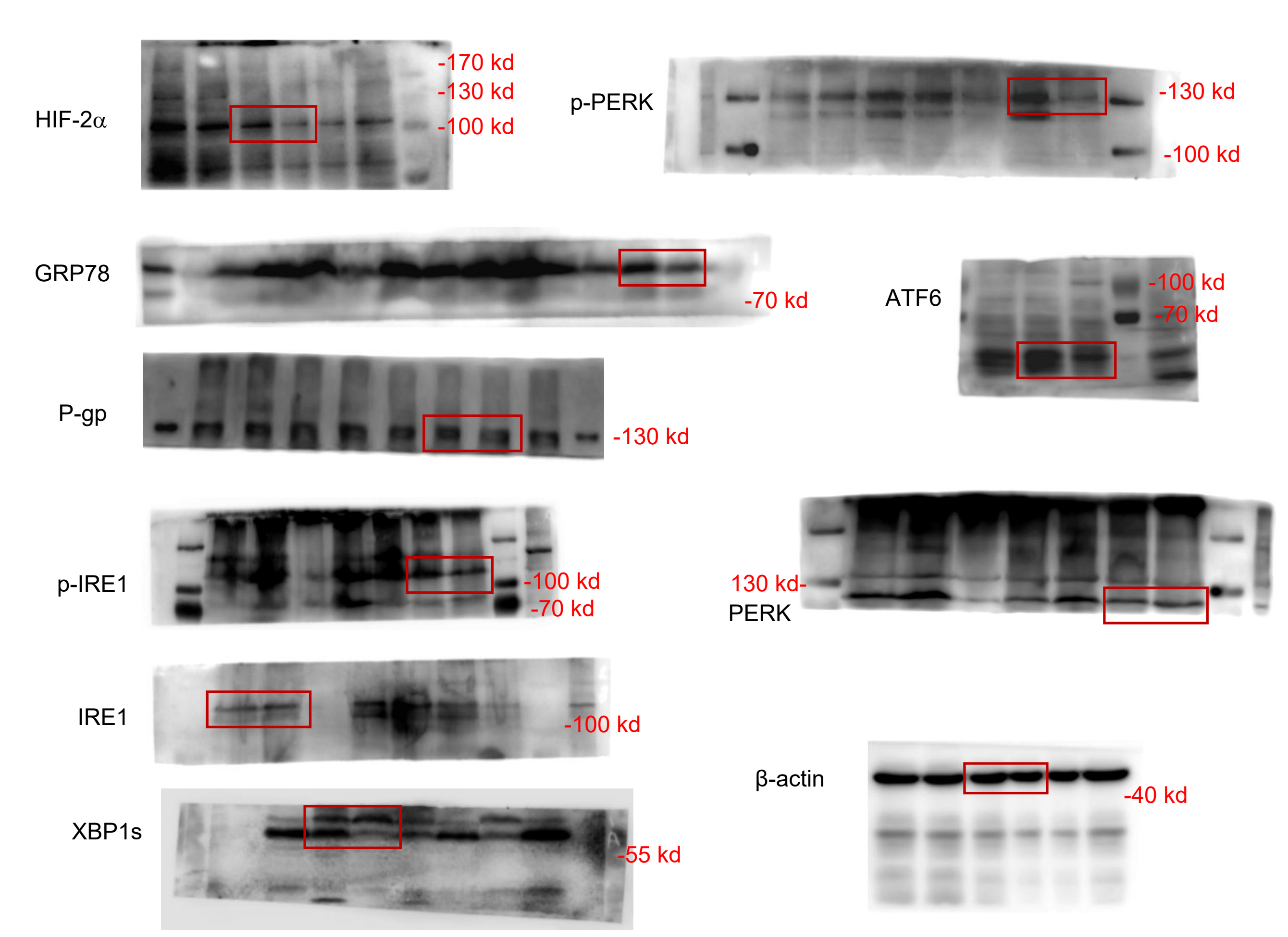

Figure 6B_MCF7 MS

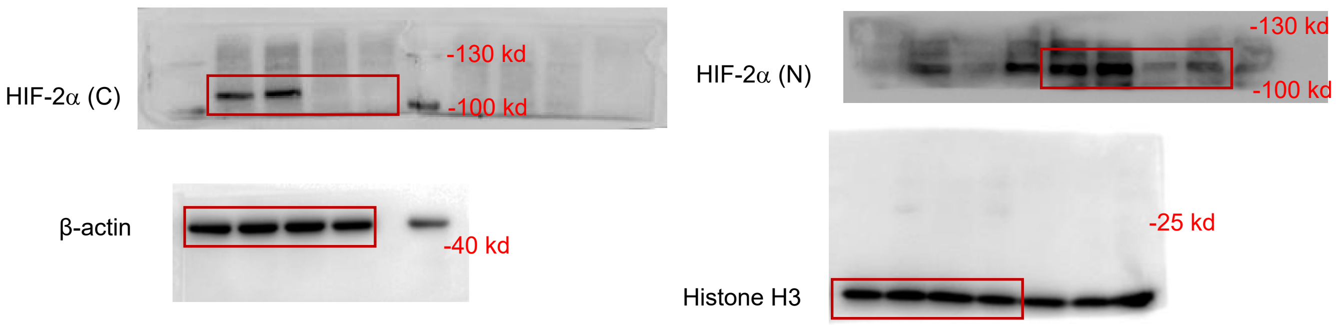

Figure 6H_MCF7 MS

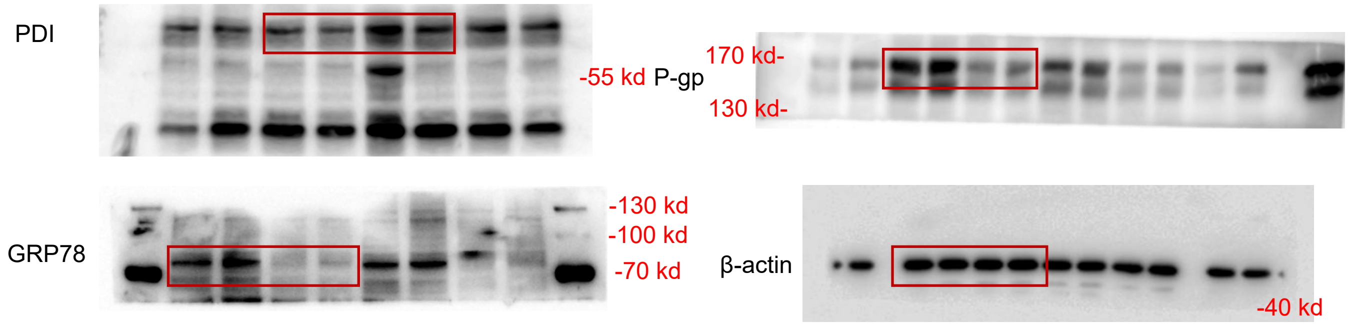

Figure 7C_tumor primary cells$^{CD44+CD24-}$

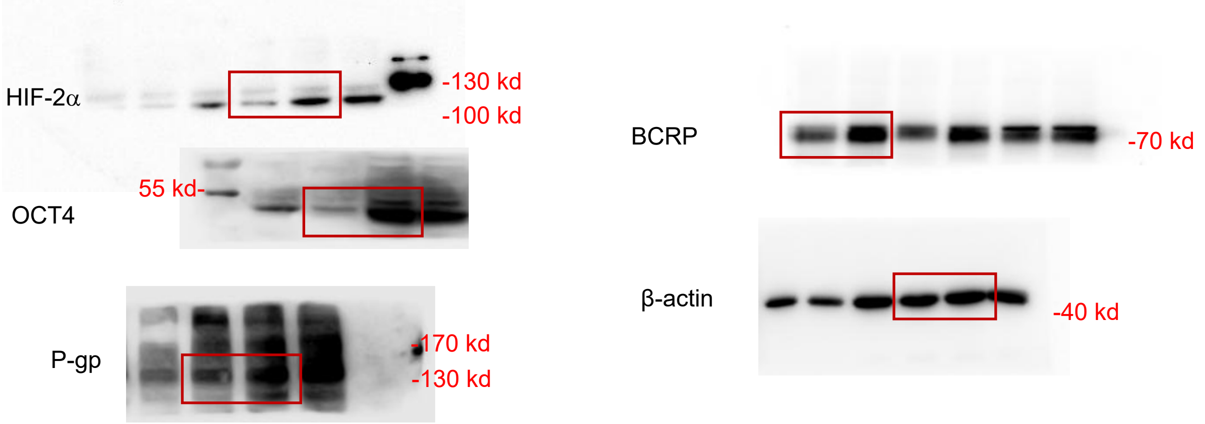

sFigure 2D_MCF7 vs MCF7 MS

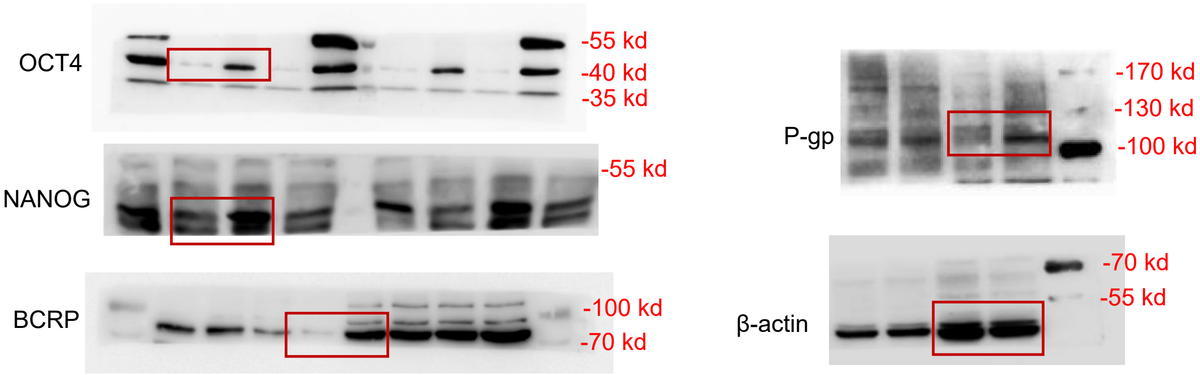

sFigure 2D_T47D vs T47D MS

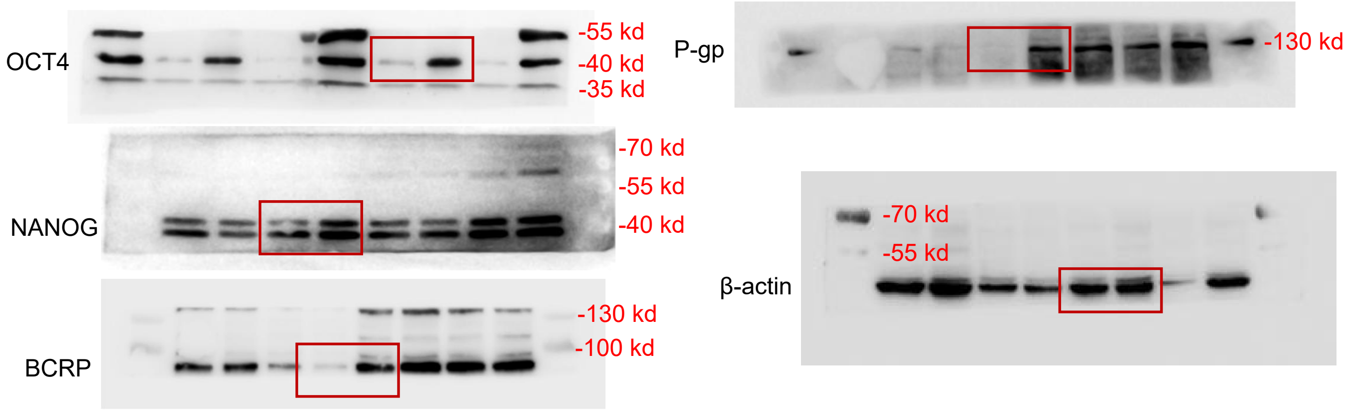

sFigure 2F_MCF7 vs MCF7 MS

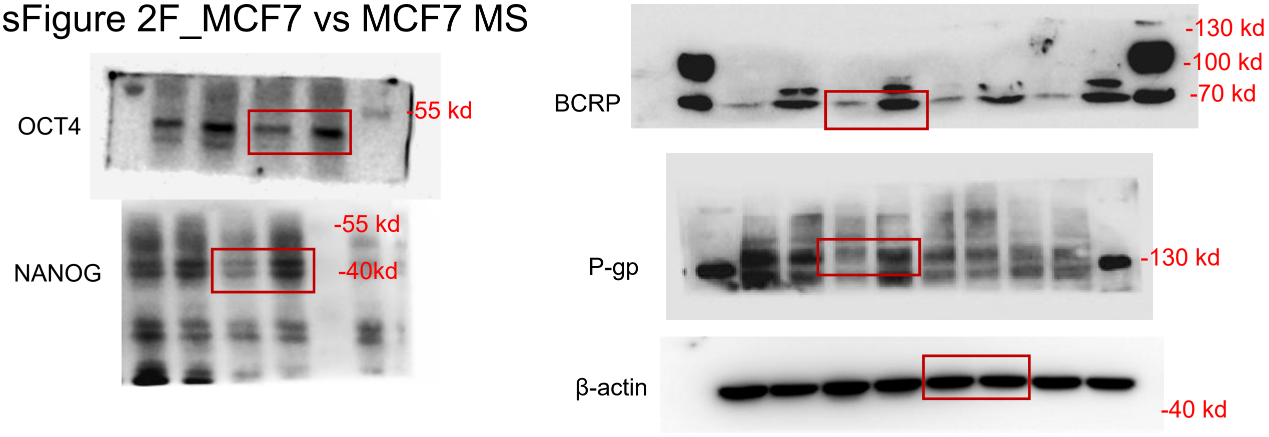

sFigure 3A_T47D VS T47D MS

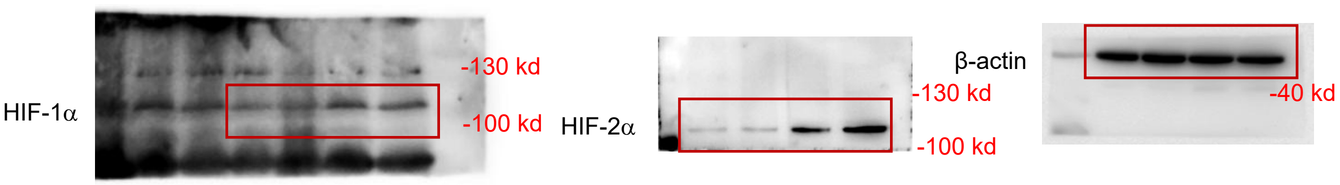

sFigure 3D_MCF7

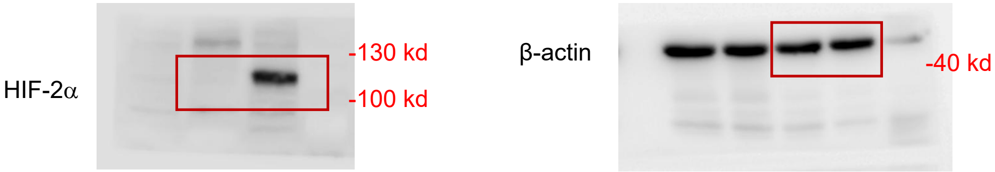

sFigure 3D_T47D

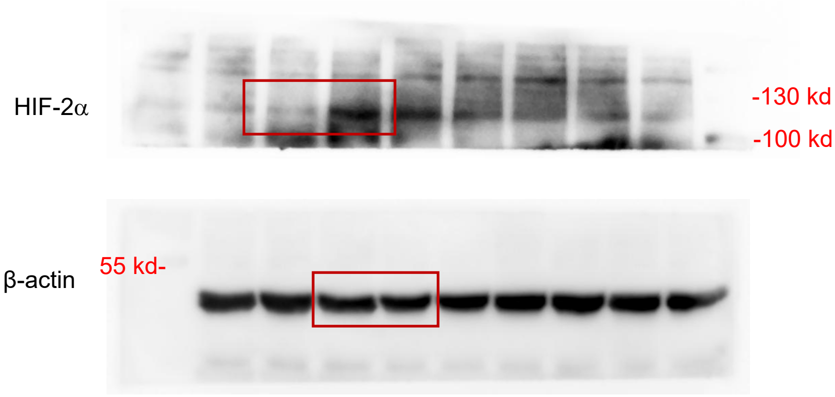

sFigure 3G_MCF7 MS

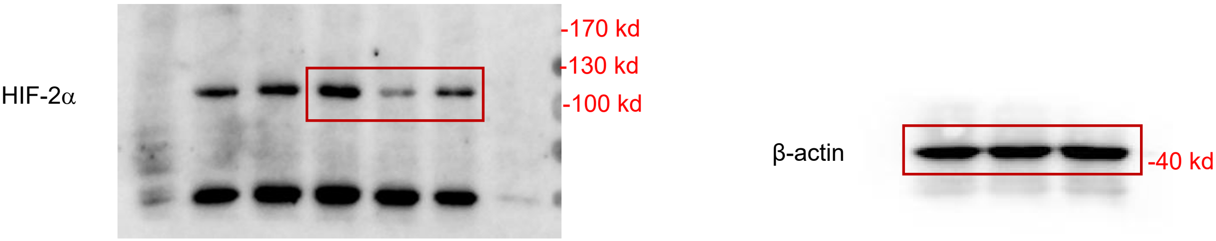

sFigure 3G_T47D MS

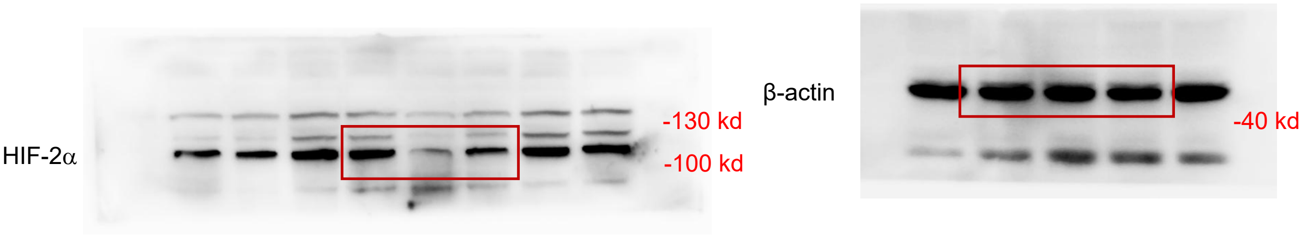

sFigure 3I_T47D

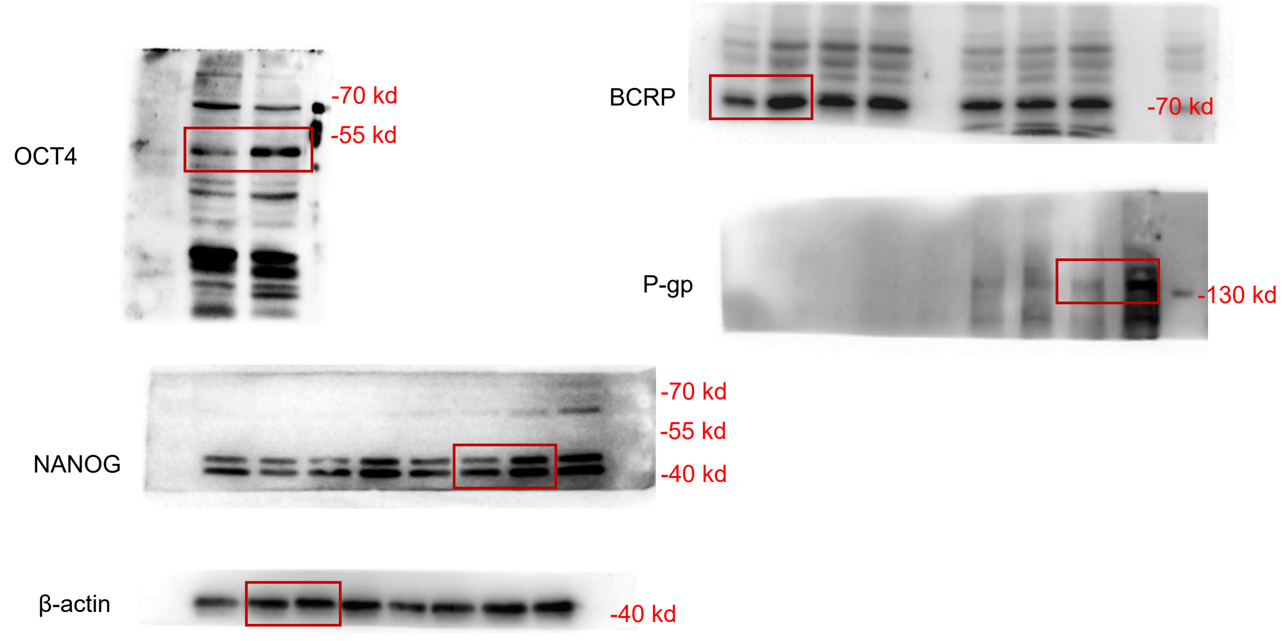

sFigure 3I_T47D MS

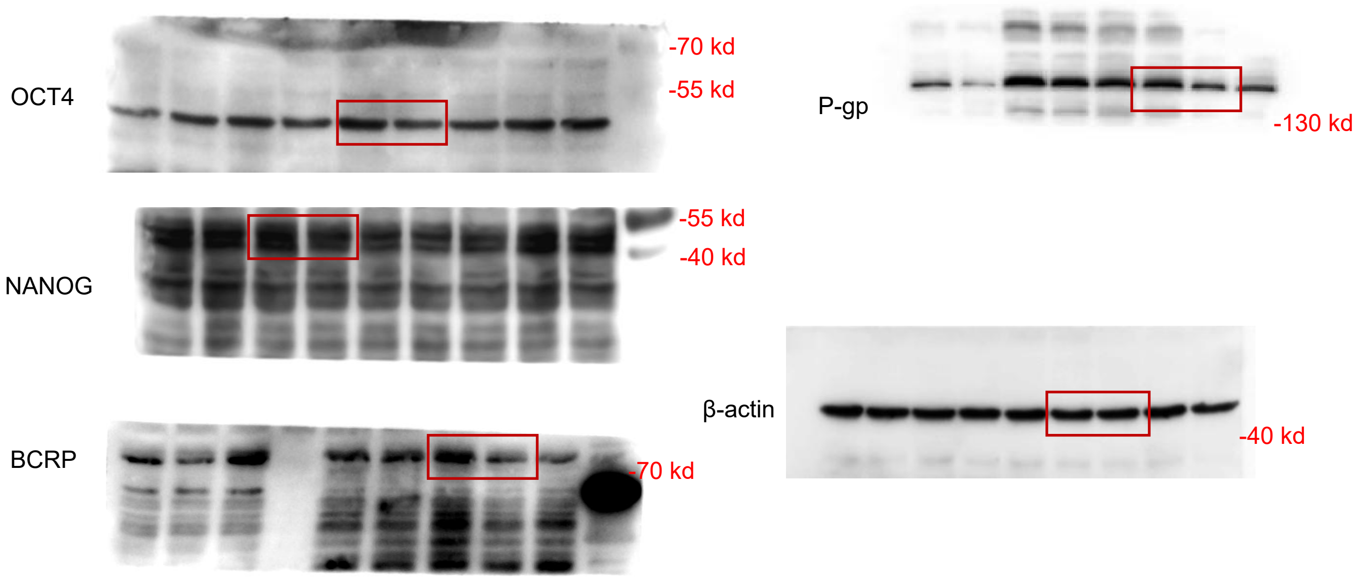

sFigure 5C_MCF7 MS

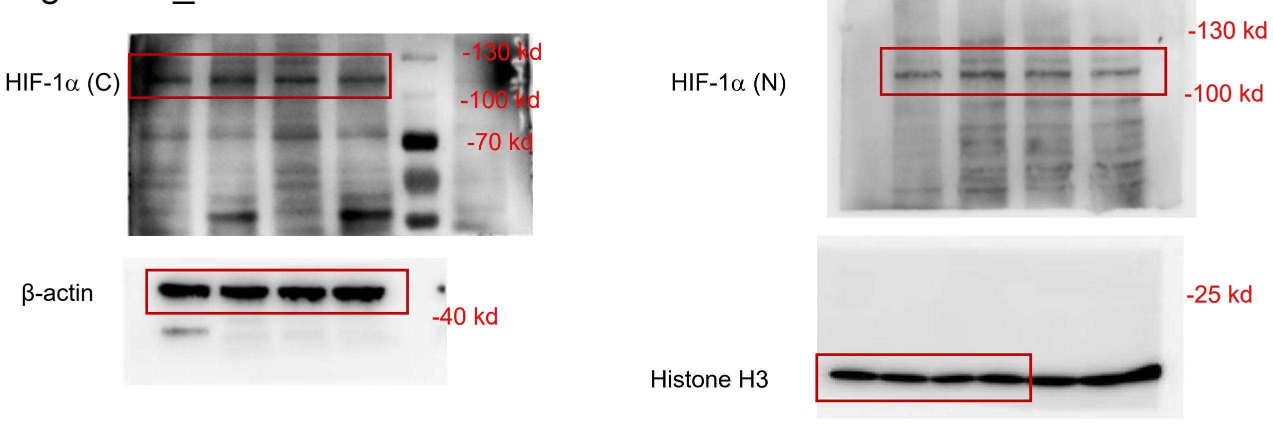

Supplement: Supplementary file 2 — Original data [file 41418_2022_963_MOESM2_ESM.pdf]
