# Supplementary material for: A novel HIF-2α targeted inhibitor suppresses hypoxia-induced breast cancer stemness via SOD2-mtROS-PDI/GPR78-UPRER axis
Source: Cell Death Differ. 2022 Mar 17;29(9):1769–89. doi: 10.1038/s41418-022-00963-8 (PMC9433403; doi:10.1038/s41418-022-00963-8)
Supplement: Supplementary file 3 — Pre-authorship [file 41418_2022_963_MOESM3_ESM.pdf]

SPRINGER NATURE

# Change of authorship request form - Journals (pre-acceptance)

## Important information. Please read.

- This form should be used by authors to request any change in authorship (adding/deleting authors) including changes in corresponding authors. This form should not be used for name changes. Please fully complete all sections. Use black ink and block capitals and provide each author's full name with the given name first followed by the family name.

- By signing this declaration, all authors guarantee that the order of the authors are in accordance with their scientific contribution, if applicable as different conventions apply per discipline, and that only authors have been added who made a meaningful contribution to the work.

- Please note, in author collaborations where there is formal agreement for representing the collaboration, it is sufficient for the representative or legal guarantor (usually the corresponding author) to complete and sign the Authorship Change Form on behalf of all authors, **next to the added/removed author(s)**. **(Complete Section 3, followed by Section 6.)**
  In author collaborations where there is no formal agreement for representing the collaboration and **there are more than 10 authors**, one may sign for all, provided the signer appends correspondence that attests that each of the authors have agreed to the change **and the added/removed authors sign the form**. **(Complete Section 3, followed by Section 6.)**

- Please note, we cannot investigate or mediate any authorship disputes. If you are unable to obtain agreement from all authors (including those who you wish to be removed) you must refer the matter to your institution(s) for investigation. Please inform us if you need to do this.

- If you are not able to return a fully completed form within **30 days** of the date that it was sent to the author requesting the change, we may have to withdraw your manuscript. We cannot publish manuscripts where authorship has not been agreed by all authors (including those who have been removed).

- Incomplete forms will be rejected.

- Please return/upload this form, fully completed, to the Journals Editorial Office. The Journal and/or Publisher will consider the information you have provided to decide whether to approve the proposed change in authorship. We may decide to contact your institution for more information or undertake a further investigation, if appropriate, before making a final decision.

SPRINGER NATURE

# Change of authorship request form - Journals (pre-acceptance)

**Section 1: Please provide the current title of manuscript**

**Manuscript ID no.:** CDD-21-0213RRRR

Title: A novel HIF-2α targeted inhibitor suppresses hypoxia-induced breast cancer stemness via SOD2-mtROS-PDI/GPR78-UPR$^{ER}$ axis

**Section 2: Please provide the previous authorship, in the order shown on the manuscript before the changes were introduced. Please indicate the corresponding author by adding (CA) behind the name.**

| | First name(s) | Family name | ORCID or SCOPUS id, if available |
|---|---|---|---|
| 1st author | Yuanyuan | Yan | 0000-0001-9406-0896 |
| 2nd author | Miao | He | 0000-0001-7258-3207 |
| 3rd author | Lin | Zhao | 0000-0001-9264-6455 |
| 4th author | Huizhe | Wu | 0000-0001-7964-1342 |
| 5th author | Yanyun | Zhao | 0000-0003-1005-2683 |
| 6th author | Li | Han | 0000-0002-3654-171X |
| 7th author | Binbin | Wei | 0000-0001-6785-4308 |
| 8th author | Dongman | Ye | 0000-0002-2901-0283 |
| 9th author | Xuemei | Lv | 0000-0002-1017-4560 |
| 10th author | Yan | Wang | 0000-0002-4006-5859 |
| 11th author | Weifan | Yao | 0000-0001-8878-2649 |
| 12th author | Haishan | Zhao | 0000-0002-8574-7677 |
| 13th author | Bo | Chen | 0000-0002-2216-2794 |
| 14th author | Zining | Jin | 0000-0002-3806-4718 |
| 15th author | Jian | Wen | 0000-0002-2297-4346 |
| 16th author | Tao | Yu | 0000-0003-4936-8861 |
| 17th author | Feng | Jin | 0000-0002-0325-5362 |
| 18th author | Minjie | Wei (CA) | 0000-0002-0404-7098 |
| 19th author | | | |
| 20th author | | | |

Please use an additional sheet if there are more than 10 authors.

---
SPRINGER NATURE Springer Nature is one of the world's leading global research, educational and professional publishers, created in May 2015 through the combination of Nature Publishing Group, Palgrave Macmillan, Macmillan Education and Springer Science+Business Media.
---

**SPRINGER NATURE**

# Change of authorship request form - Journals (pre-acceptance)

**Section 3: Please provide a justification for change. Please use this section to explain your reasons for changing the authorship of your manuscript, e.g. what necessitated the change in authorship? Please refer to the (journal) policy pages for more information about authorship. Please explain why omitted authors were not originally included and/or why authors were removed on the submitted manuscript.**

Yan Zhu was added as one of the authors in the manuscript submitted for the final version. During the procession of revision, Yan Zhu mainly participated in the implementation of supplementary experiments, especially the COIP experiment of Figure 4I. Therefore, we did not change the ranking of other authors, just added Yan Zhu as one of the authors of this article.

**Section 4: Proposed new authorship. Please provide your new authorship list in the order you would like it to appear on the manuscript. Please indicate the corresponding author by adding (CA) behind the name. <u>If the Corresponding Author has changed, please indicate the reason under section 3</u>.**

| | First name(s) | Family name (this name will appear in full on the final publication and will be searchable in various abstract and indexing databases) | Affiliated institute | E-mail address |
|---|---|---|---|---|
| 1st author | Yuanyuan | Yan | Department of Pharmacology, CMU | yanyuanyuan1987@163.com |
| 2nd author | Miao | He | Department of Pharmacology, CMU | hemiao_cmu@126.com |
| 3rd author | Lin | Zhao | Department of Pharmacology, CMU | zl_cmu@163.com |
| 4th author | Huizhe | Wu | Department of Pharmacology, CMU | wuhz@cmu.edu.cn |
| 5th author | Yanyun | Zhao | Department of Pharmacology, CMU | zhaoyanyun8@163.com |
| 6th author | Li | Han | Department of Pharmacology, CMU | han_cmu@163.com |
| 7th author | Binbin | Wei | Department of Pharmacology, CMU | bbwei@cmu.edu.cn |
| 8th author | Dongman | Ye | Department of Medical Imaging, Cancer Hospital of CMU | cmu_ydm@163.com |
| 9th author | Xuemei | Lv | Department of Pharmacology, CMU | xmlv@cmu.edu.cn |
| 10th author | Yan | Wang | Department of Pharmacology, CMU | cmuwy@126.com |

---
**SPRINGER NATURE** Springer Nature is one of the world's leading global research, educational and professional publishers, created in May 2015 through the combination of Nature Publishing Group, Palgrave Macmillan, Macmillan Education and Springer Science+Business Media.
---

SPRINGER NATURE

# Change of authorship request form - Journals (pre-acceptance)

| | First name(s) | Family name (this name will appear in full on the final publication and will be searchable in various abstract and indexing databases) | Affiliated institute | E-mail address |
|---|---|---|---|---|
| 11th author | Weifan | Yao | Department of Pharmacology, CMU | ywf3209@163.com |
| 12th author | Haishan | Zhao | Department of Pharmacology, CMU | haishan_zhao@126.com |
| 13th author | Bo | Chen | Department of Breast Surgery, The First Affiliated Hospital of CMU | bochen@cmu.edu.cn |
| 14th author | Zining | Jin | Department of Breast Surgery, The First Affiliated Hospital of CMU | m13889353565@163.com |
| 15th author | Jian | Wen | Department of Breast Surgery, The Fourth Affiliated Hospital of CMU | wenjian_163@hotmail.com |
| 16th author | Yan | Zhu | Department of Pharmacology, CMU | zhuyan@cmu.edu.cn |
| 17th author | Tao | Yu | Department of Medical Imaging, Cancer Hospital of CMU | yutao@cancerhosp-ln-cmu.com |
| 18th author | Feng | Jin | Department of Breast Surgery, The First Affiliated Hospital of CMU | jinfeng@cmu.edu.cn |
| 19th author | Minjie | Wei (CA) | Department of Pharmacology, CMU | weiminjiecmu@163.com |
| 20th author | | | | |

Please use an additional sheet if there are more than 10 authors.

SPRINGER NATURE

Springer Nature is one of the world's leading global research, educational and professional publishers, created in May 2015 through the combination of Nature Publishing Group, Palgrave Macmillan, Macmillan Education and Springer Science+Business Media.

**SPRINGER NATURE** 

# Change of authorship request form - Journals (pre-acceptance)

**Section 5: Author contribution, Acknowledgement and Disclosures. Please use this section to provide a new disclosure statement and, if appropriate, acknowledge any contributors who have been removed as authors and ensure you state what contribution any new authors made (if applicable per the journal or book (series) policy). Please ensure these are updated in your manuscript - after approval of the change(s) - as our production department will not transfer the information in this form to your manuscript.**

**New acknowledgements:**

The authors would like to acknowledge the Key Laboratory of Precision Diagnosis and Treatment of Gastrointestinal Tumors, Ministry of Education (China Medical University, Shenyang, China) for providing the space and equipment for conducting the experiments.

**New Disclosures (financial and non-financial interests, funding):**

This work was supported by the National Natural Science Foundation of China (NSFC, No. 81373427, 81902708, 81972794, 81702616), NSFC-Liaoning joint fund key program (No. U20A20413), Key R&D Guidance Plan Projects in Liaoning Province (2019JH8/10300011), and Shenyang S&T Projects (19-109-4-09, 20-204-4-22), Program for Shenyang High Level Talent Innovation and Entrepreneurship Team (2019-SYRCCY-B-01), Major Special S&T Projects in Liaoning Province (2019JH1/10300005)， Liaoning provincial department of education scientific research project (QN2019034), Science and Technology Program of Liaoning Province (2017225036).

**New Author Contributions statement (if applicable per the journal policy):**

YYY, MJW, FJ, TY and MH conceived the study design. YYY, YYZ, LH, BBW, DMY, XML, YW, WFY, HSZ, BC, ZNJ, JW, and YZ performed experiments and collected all data. YYY, MJW, TY, FJ, MH, LZ, HZW, and collected and analyzed data and drafted the manuscript. All authors read and approved the final manuscript.

State 'Not applicable' if there are no new authors.

---

**SPRINGER NATURE** Springer Nature is one of the world's leading global research, educational and professional publishers, created in May 2015 through the combination of Nature Publishing Group, Palgrave Macmillan, Macmillan Education and Springer Science+Business Media.

---

SPRINGER NATURE

# Change of authorship request form - Journals (pre-acceptance)

**Section 6: Declaration of agreement. *All* authors, unchanged, new and removed *must* sign this declaration.**
(NB: Please print the form, (docu)-sign and return/upload a scanned copy. Please note that signatures that have been inserted as an image file are acceptable as long as it is handwritten. Typed names in the signature box are unacceptable.) *** Please delete as appropriate. Delete all of the bold if you were on the original authorship list and are remaining as an author.**

| | First name | Family name | | Signature | Date |
|---|---|---|---|---|---|
| 1st author | Yuanyuan | Yan | I agree to the proposed new authorship shown in section 4 and the proposed change in corresponding author | Yuanyuan Yan | 2/11/2022 |
| 2nd author | Miao | He | I agree to the proposed new authorship shown in section 4 and the proposed change in corresponding author | Miao He | 2/11/2022 |
| 3rd author | Lin | Zhao | I agree to the proposed new authorship shown in section 4 and the proposed change in corresponding author | Lin Zhao | 2/11/2022 |
| 4th authors | Huizhe | Wu | I agree to the proposed new authorship shown in section 4 and the proposed change in corresponding author | Huizhe Wu | 2/11/2022 |
| 5th author | Yanyun | Zhao | I agree to the proposed new authorship shown in section 4 and the proposed change in corresponding author | Yanyun Zhao | 2/11/2022 |
| 6th author | Li | Han | I agree to the proposed new authorship shown in section 4 and the proposed change in corresponding author | Li Han | 2/12/2022 |
| 7th author | Binbin | Wei | I agree to the proposed new authorship shown in section 4 and the proposed change in corresponding author | Binbin Wei | 2/11/2022 |

---
SPRINGER NATURE Springer Nature is one of the world's leading global research, educational and professional publishers, created in May 2015 through the combination of Nature Publishing Group, Palgrave Macmillan, Macmillan Education and Springer Science+Business Media.
---

SPRINGER NATURE

# Change of authorship request form - Journals (pre-acceptance)

| | First name | Family name | | Signature | Date |
|---|---|---|---|---|---|
| 8th author | Dongman | Ye | I agree to the proposed new authorship shown in section 4 and the proposed change in corresponding author | Dongman Ye | 2/12/2022 |
| 9th author | Xuemei | Lv | I agree to the proposed new authorship shown in section 4 and the proposed change in corresponding author | Xuemei Lv | 2/12/2022 |
| 10th author | Yan | Wang | I agree to the proposed new authorship shown in section 4 and the proposed change in corresponding author | Yan Wang | 2/11/2022 |
| 11th author | Weifan | Yao | I agree to the proposed new authorship shown in section 4 and the proposed change in corresponding author | Weifan Yao | 2/12/2022 |
| 12th author | Haishan | Zhao | I agree to the proposed new authorship shown in section 4 and the proposed change in corresponding author | Haishan Zhao | 2/11/2022 |
| 13th author | Bo | Chen | I agree to the proposed new authorship shown in section 4 and the proposed change in corresponding author | Bo Chen | 2/11/2022 |
| 14th author | Zining | Jin | I agree to the proposed new authorship shown in section 4 and the proposed change in corresponding author | Zining Jin | 2/12/2022 |
| 15th author | Jian | Wen | I agree to the proposed new authorship shown in section 4 and the proposed change in corresponding author | Jian Wen | 2/12/2022 |
| 16th author | Yan | Zhu | I agree to the proposed new authorship shown in section 4 **and the addition** and the proposed change in corresponding author | Yan Zhu | 2/12/2022 |

Please use an additional sheet if there are more than 10 authors.

**SPRINGER NATURE**

# Change of authorship request form - Journals (pre-acceptance)

| | First name | Family name | | Signature | Date |
|---|---|---|---|---|---|
| 17th author | Tao | Yu | I agree to the proposed new authorship shown in section 4 and the proposed change in corresponding author | Tao Yu | 2/12/2022 |
| 18th author | Feng | Jin | I agree to the proposed new authorship shown in section 4 and the proposed change in corresponding author | Feng Jin | 2/11/2022 |
| 19th author | Minjie | Wei (CA) | I agree to the proposed new authorship shown in section 4 and the proposed change in corresponding author | Minjie Wei | 2/11/2022 |
| 20th author | | | | | |

**In case of author collaborations with formal agreement:**

| | Name of consortium/consortia | First name | Family name | | Signature | Date |
|---|---|---|---|---|---|---|
| Representative/legal guarantor | | | | I agree to the proposed new authorship shown in section 4 **/and the addition/removal*of my name to the authorship list** /and the proposed change in corresponding author | | |

**Both added/removed authors should complete the information in the first table under Section 6.**

---- End of form ----

---
**SPRINGER NATURE** Springer Nature is one of the world's leading global research, educational and professional publishers, created in May 2015 through the combination of Nature Publishing Group, Palgrave Macmillan, Macmillan Education and Springer Science+Business Media.
---
